# Supplementary material for: Genomic diversity landscape of the honey bee gut microbiota
Source: Nat Commun. 2019 Jan 25;10:446. doi: 10.1038/s41467-019-08303-0 (PMC6347622; doi:10.1038/s41467-019-08303-0)
Supplement: Supplementary file 1 — Supplementary Information [file 41467_2019_8303_MOESM1_ESM.pdf]

Genomic diversity landscape of the honey bee gut microbiota

Kirsten M Ellegaard, Philipp Engel

Supplementary information:

Supplementary Figures 1-18

Supplementary Tables 1-2

Supplementary References

Supplementary Figures

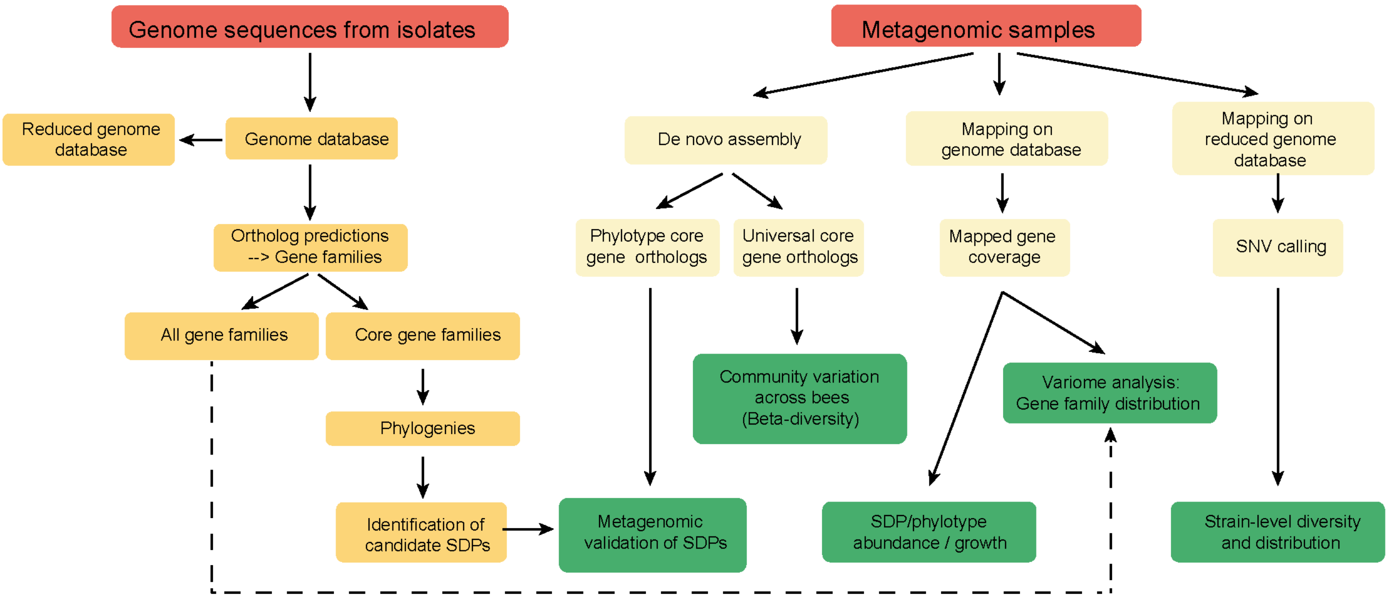

Supplementary Figure 1. Flowchart of the overall bioinformatic analysis. Two types of input data were used: (i) genome sequences from bacterial isolates, previously published or sequenced for this study, (ii) Illumina reads from 54 metagenomes (shown in red). The five major outputs of the overall approach are shown in green. All intermediate steps are shown in two different tones of yellow (darker color, steps involving genomic data; lighter color, steps involving metagenomic data). Flowcharts depicting the details of each analysis leading to the five major outputs are illustrated in Supplementary Figures 3, 7, 11, 13, and 17.

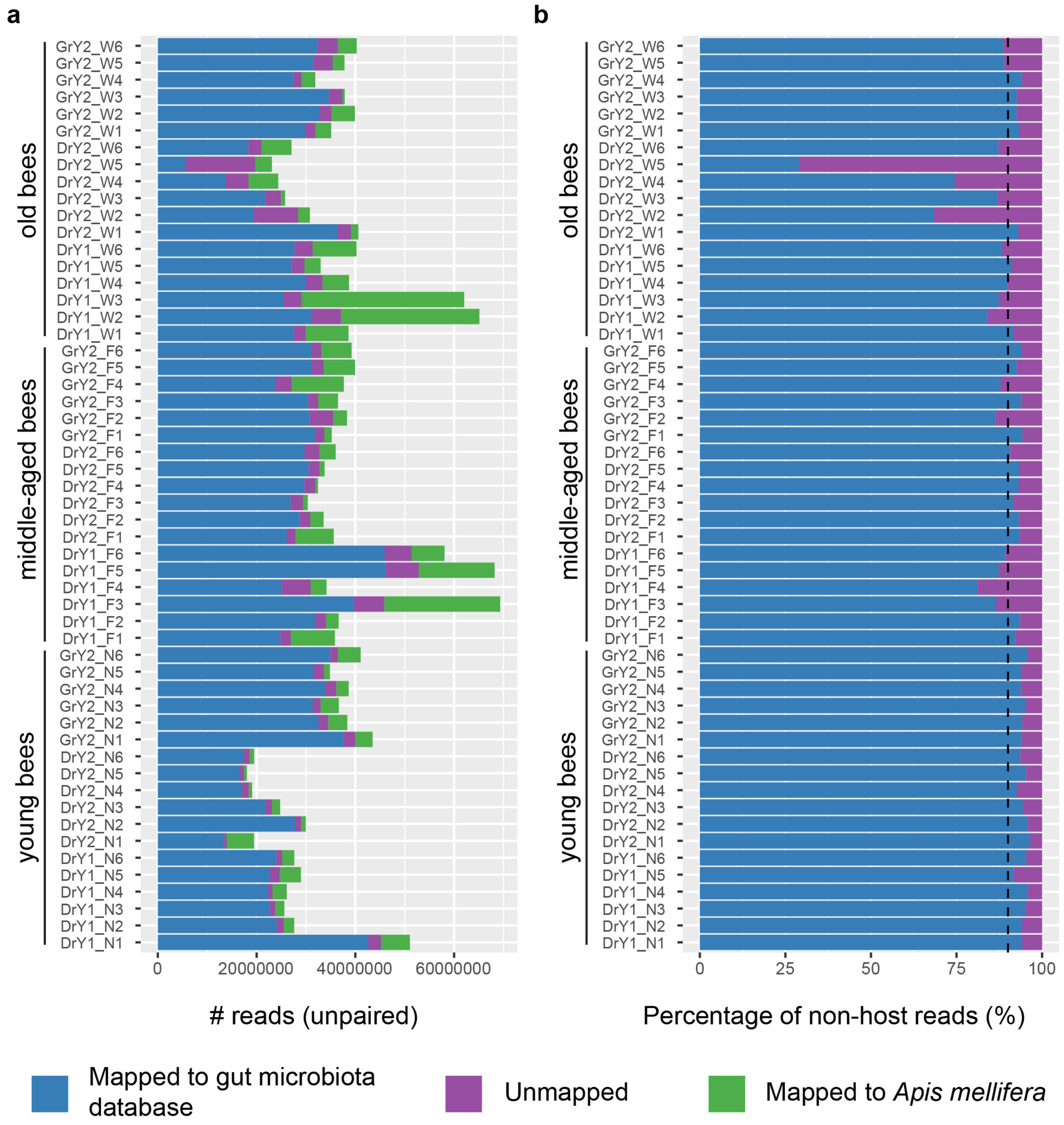

Supplementary Figure 2. Number and origin of the metagenomic reads of each sample. In blue, reads mapped to the current gut microbiota database; in magenta, reads neither mapped to the gut microbiota database nor the honey bee genome; in green, reads mapped to the honey bee genome. **a,** the number of mapped and unmapped reads, and **b,** the percentage of non-host reads mapped to the bee gut microbiota database (blue) or unmapped (magenta). Samples are ordered according to honey bee age group. DrY1 and DrY2 stand for colony 1 ("Les Droites) in year 1 and year 2, respectively. GrY2 ("Grammont") stands for colony 2 in year 2.

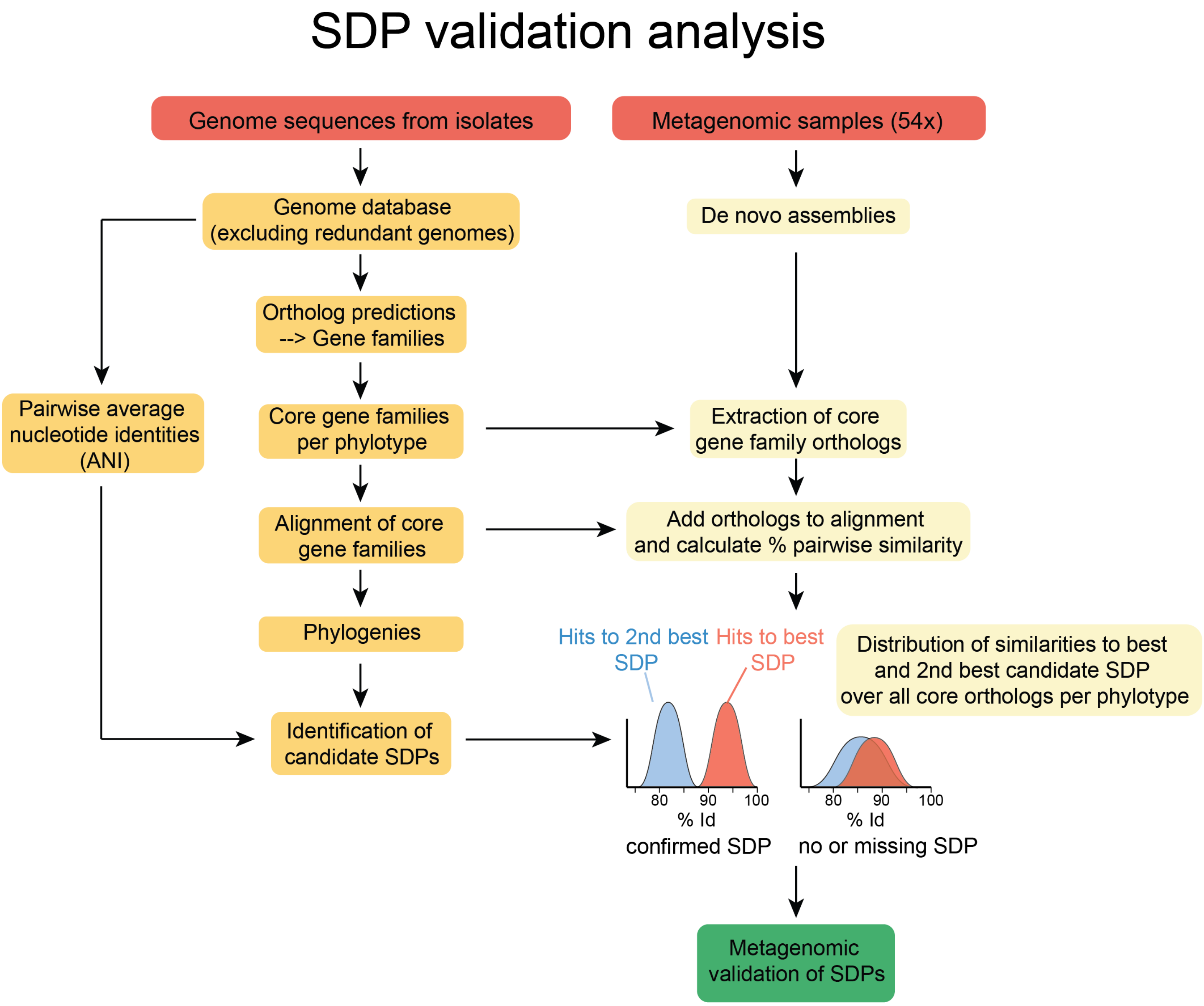

Supplementary Figure 3. Flowchart of the analysis to identify and validate SDPs within phylotypes of the bee gut microbiota. To identify candidate SDPs, a genome database from all available genomes was constructed and orthologs determined for each phylotype separately. Single-copy core gene families were then extracted, aligned, and concatenated, and a genome-wide phylogeny was inferred for each phylotype. Based on these phylogenies and pairwise average nucleotide identities (ANI) between genomes, candidate SDP were identified, i.e. genomes that cluster together. These candidate SDPs were then validated with the metagenomic data. To this end, each metagenome was *de novo* assembled. Then, metagenomic orthologs of the core gene families from the genomic database were extracted for each phylotype and added to the alignment of the corresponding gene family. For each metagenomic ortholog,

the maximum pairwise percentage identity in the alignment to the highest and second-highest scoring SDP was recorded and the distributions plotted over all core genes and metagenomes for a given phylotype. Well-separated distributions indicate that all metagenomic orthologs unambiguously cluster with one candidate SDP (left distribution plot). If large amounts of metagenomic orthologs exhibit similar pairwise identities to genomes from two different SDPs, then the distributions will overlap. In this case, we can conclude that either the candidate SDPs are not well separated or that there are additional SDPs that are equally divergent from the identified candidate SDPs. The flowchart colors are the same as for Supplementary Figure 1.

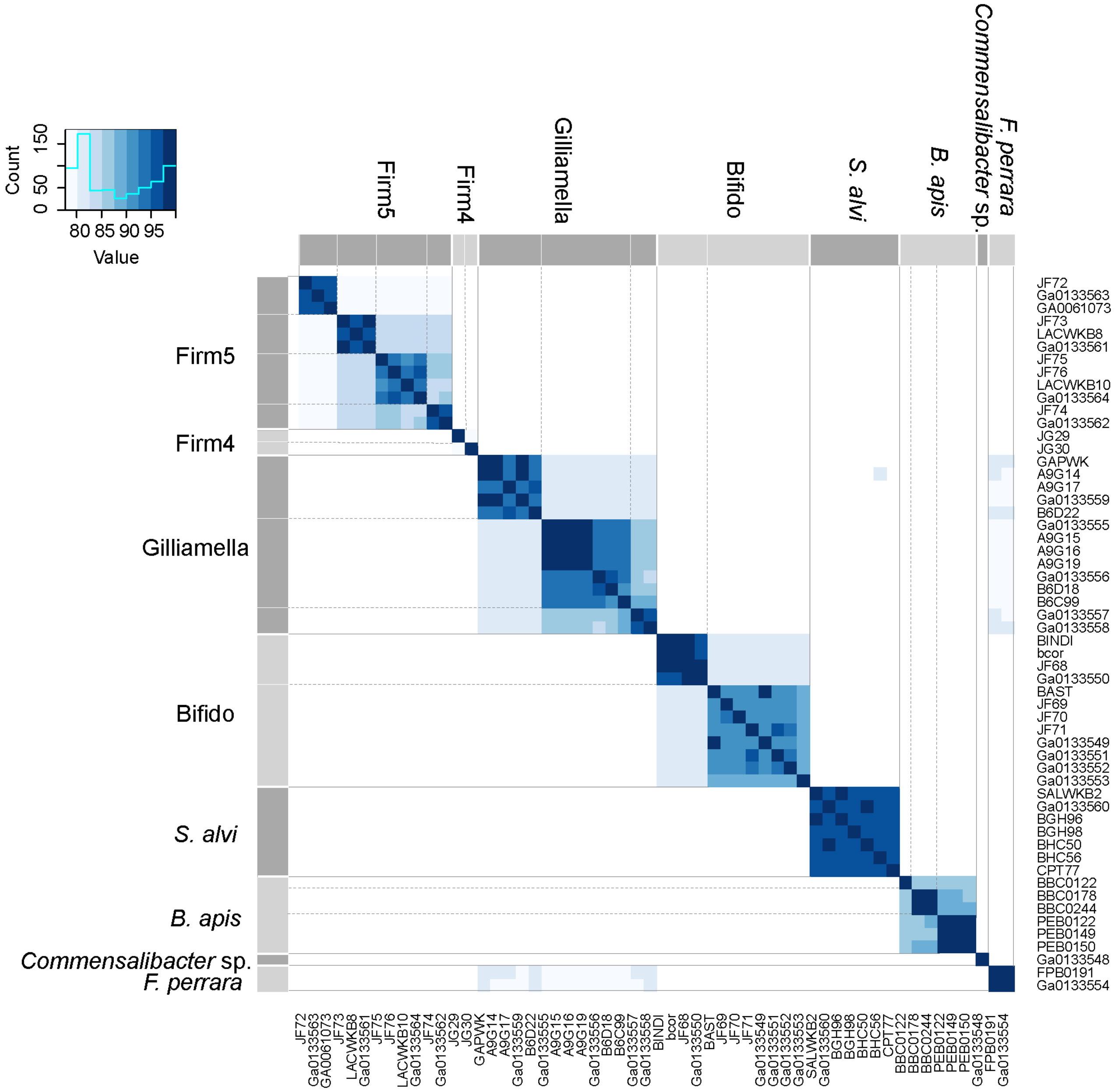

Supplementary Figure 4. Heatmap of pairwise ANI (average nucleotide identity). Intensity of heatmap indicates pairwise ANI. White areas correspond to genomes, which were too divergent for ANI calculation. Phylotypes and candidate SDPs (as based on ANI values and phylogenies) are indicated by grey bars and dotted lines, respectively. Phylotype and strain names of each genome included in the analysis are given next to the plot area (see also Supplementary Data 1).

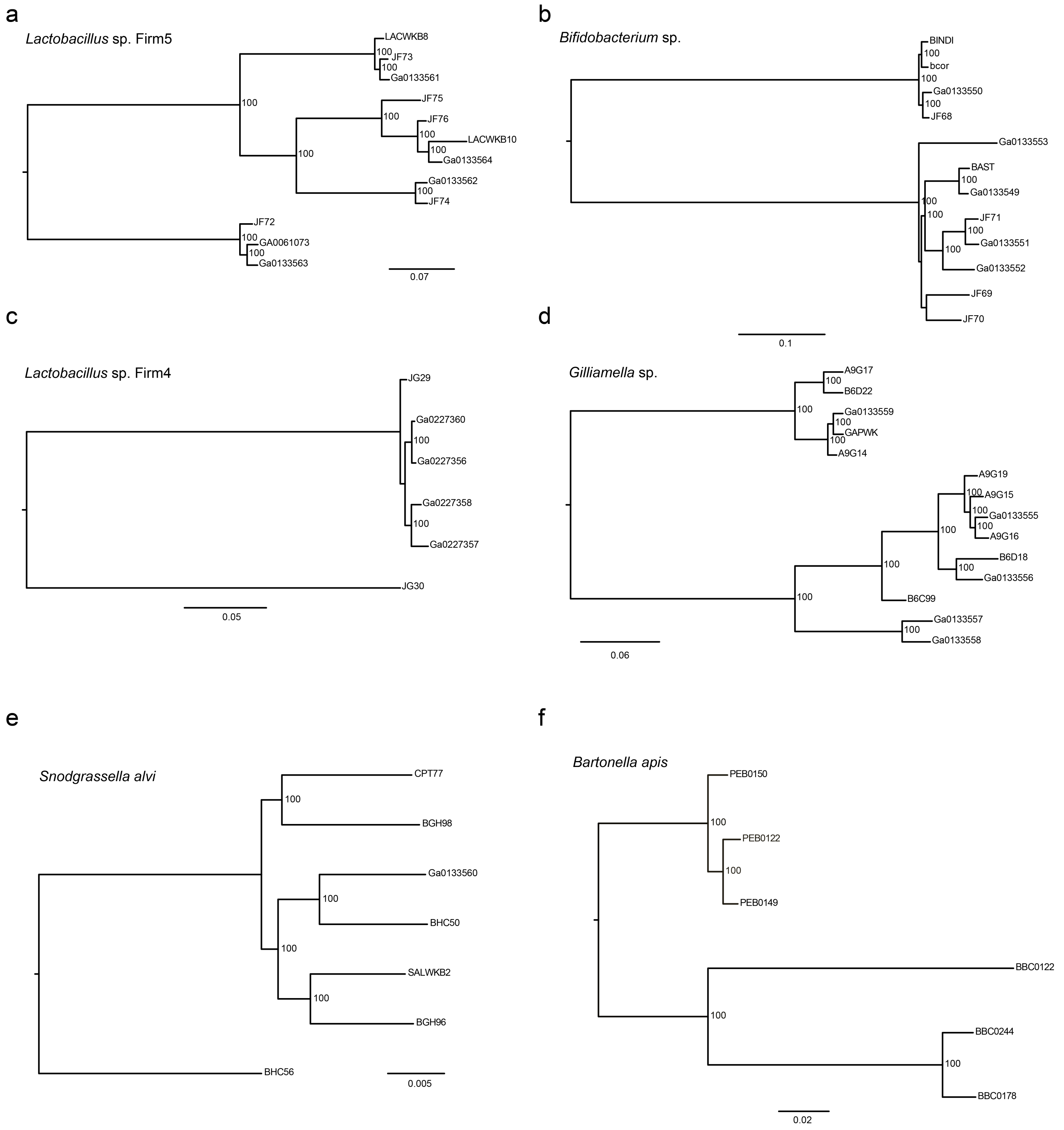

Supplementary Figure 5. Core genome phylogenies of bee gut microbiota members. a-f, Fully annotated versions of the trees as shown in Figure 2. Phylogenetic analyses were based on nucleotide alignments, except in the case of Firm4. Bars correspond to substitutions per site. Labels on leaves correspond to locus-tags/strain-names, as given in Supplementary Data 1.

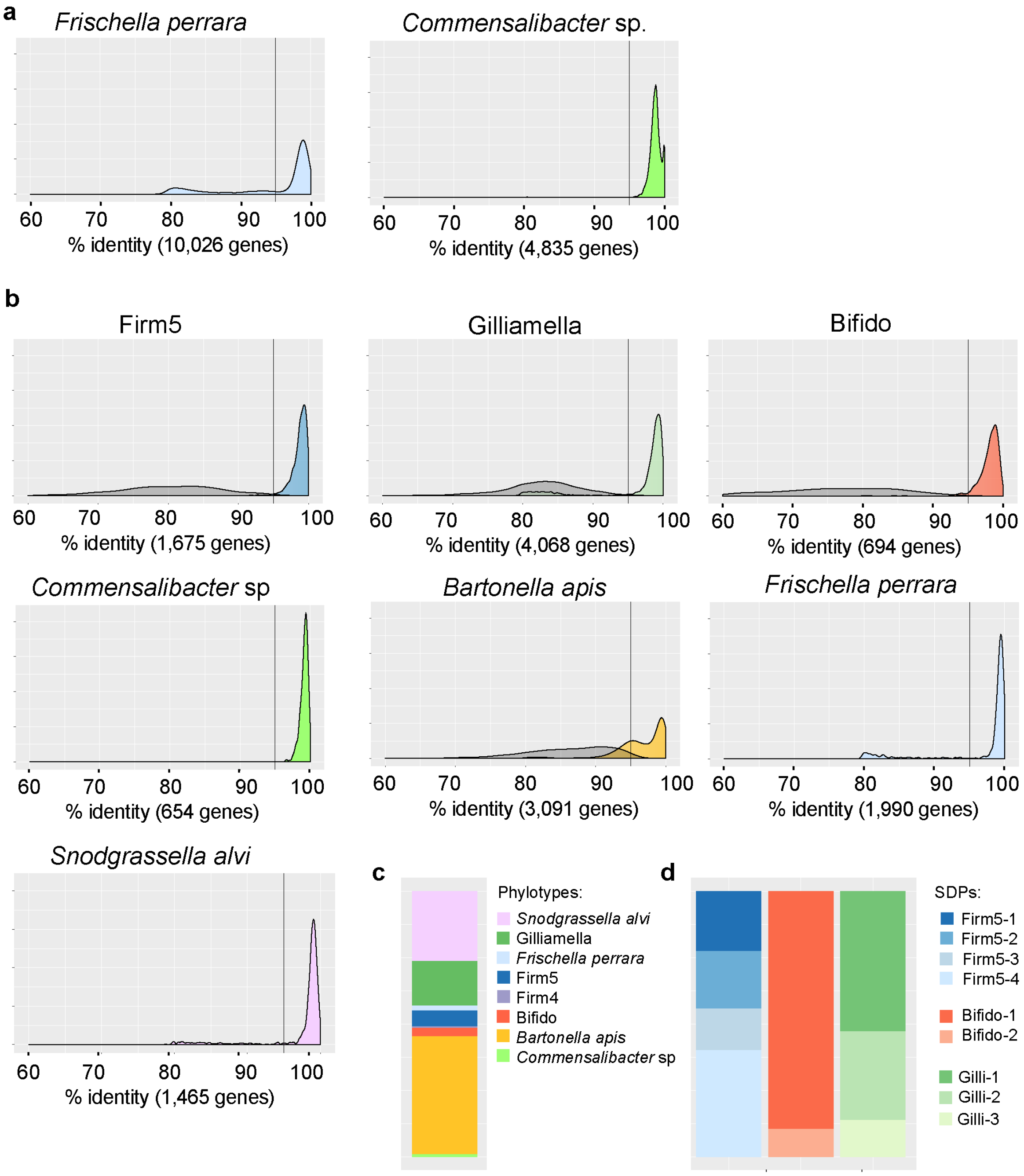

Supplementary Figure 6. Density plots for two non-core phylotypes in the current dataset and complete phylotype/SDP analysis of a previously published metagenomic dataset. Density plots for, **a,** the two remaining phylotypes of the current study that are not shown in Figure 2, and **b**, for seven phylotypes in the previously published metagenomic dataset (derived from 150 pooled honey bee guts from a single colony in the U.S.)[1]. Pairwise identity distributions of metagenomic orthologs are shown for the highest and the second-highest scoring SDP as in Figure 3. No results are shown for the core phylotype Firm4, since only 6 putative orthologous sequences were extracted from the assembly, likely

because the abundance was too low for genome assembly for this member. Based on mapping to the genomic database, both Firm4-1 and Firm4-2 were found to be present (more than 80% of the core genes had a mean coverage above 1). **c**, Composition of the gut microbiota at the phylotype level for the previously published metagenomic sample. **d,** Composition of the gut microbiota at the SDP level for the previously published metagenomic sample. SDP level composition is shown for phylotypes Firm5, Bifido and Gilliamella, but not for Firm4 due to the low abundance of this member in the sample.

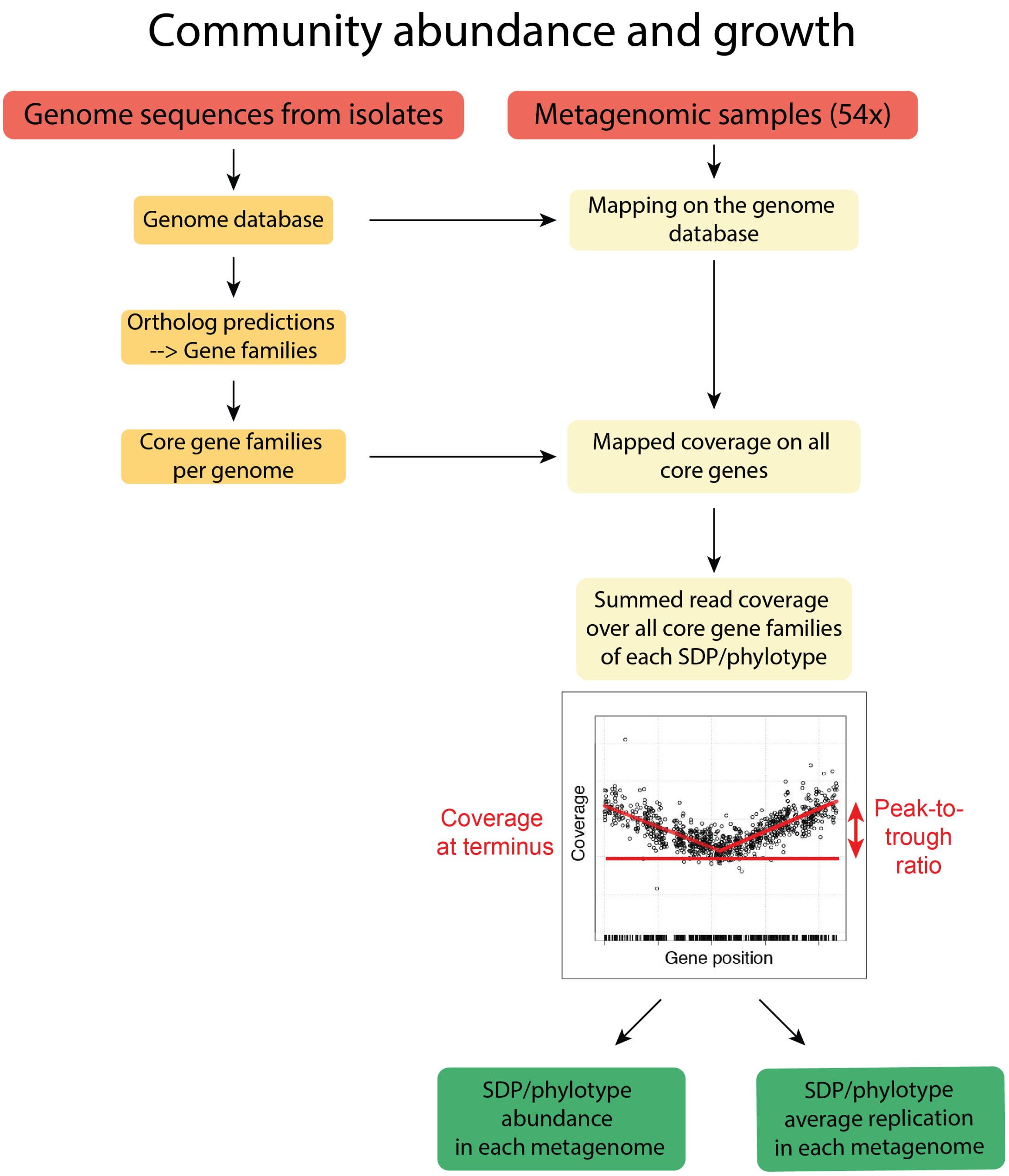

Supplementary Figure 7. Flowchart of the analysis to determine phylotype and SDP abundance and average population replication. Metagenomic reads of each sample were mapped on the reference genome database and the coverage of all core genes in the reference database was calculated. The read coverage was then summed over all members of a given core gene family for each phlyotype or SDP, and plotted on a single reference genome according to the gene position on the chromosome. For most samples, higher overage was found at the origin as compared to at the terminus, indicative of actively replicating populations. In these cases, the core gene family coverage at the terminus was used to determine phylotype/SDP abundance. Otherwise the median core gene family coverage was used. For

actively growing populations, we also inferred the average population replication by calculating the peak-trough ratio (PTR) (the coverage at origin relative to the coverage at terminus of replication). The flowchart colors are the same as for Supplementary Figure 1.

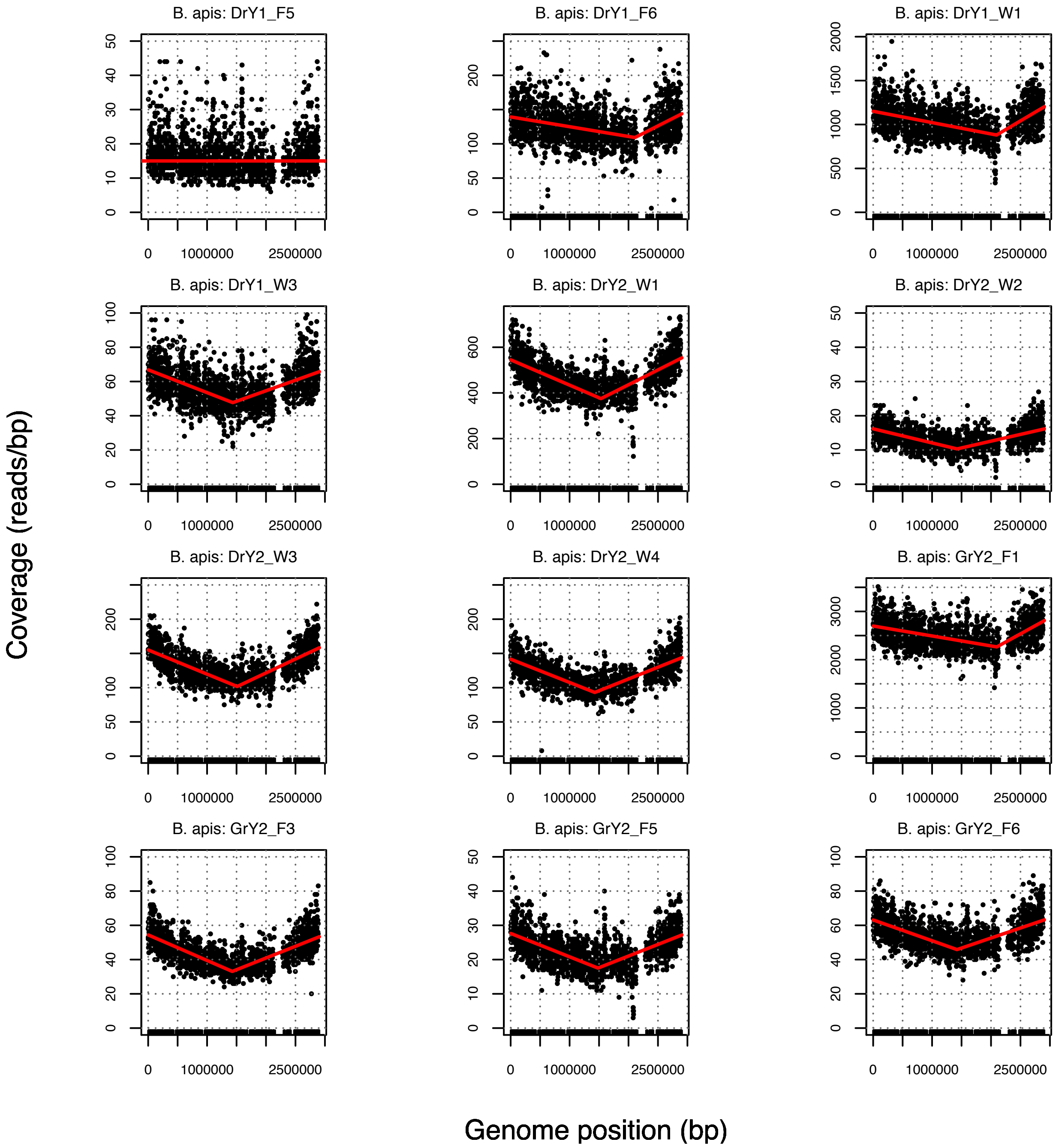
B. apis: DrY1_F5
B. apis: DrY1_F6
B. apis: DrY1_W1
B. apis: DrY1_W3
B. apis: DrY2_W1
B. apis: DrY2_W2
B. apis: DrY2_W3
B. apis: DrY2_W4
B. apis: GrY2_F1
B. apis: GrY2_F3
B. apis: GrY2_F5
B. apis: GrY2_F6
Coverage (reads/bp)
Genome position (bp)

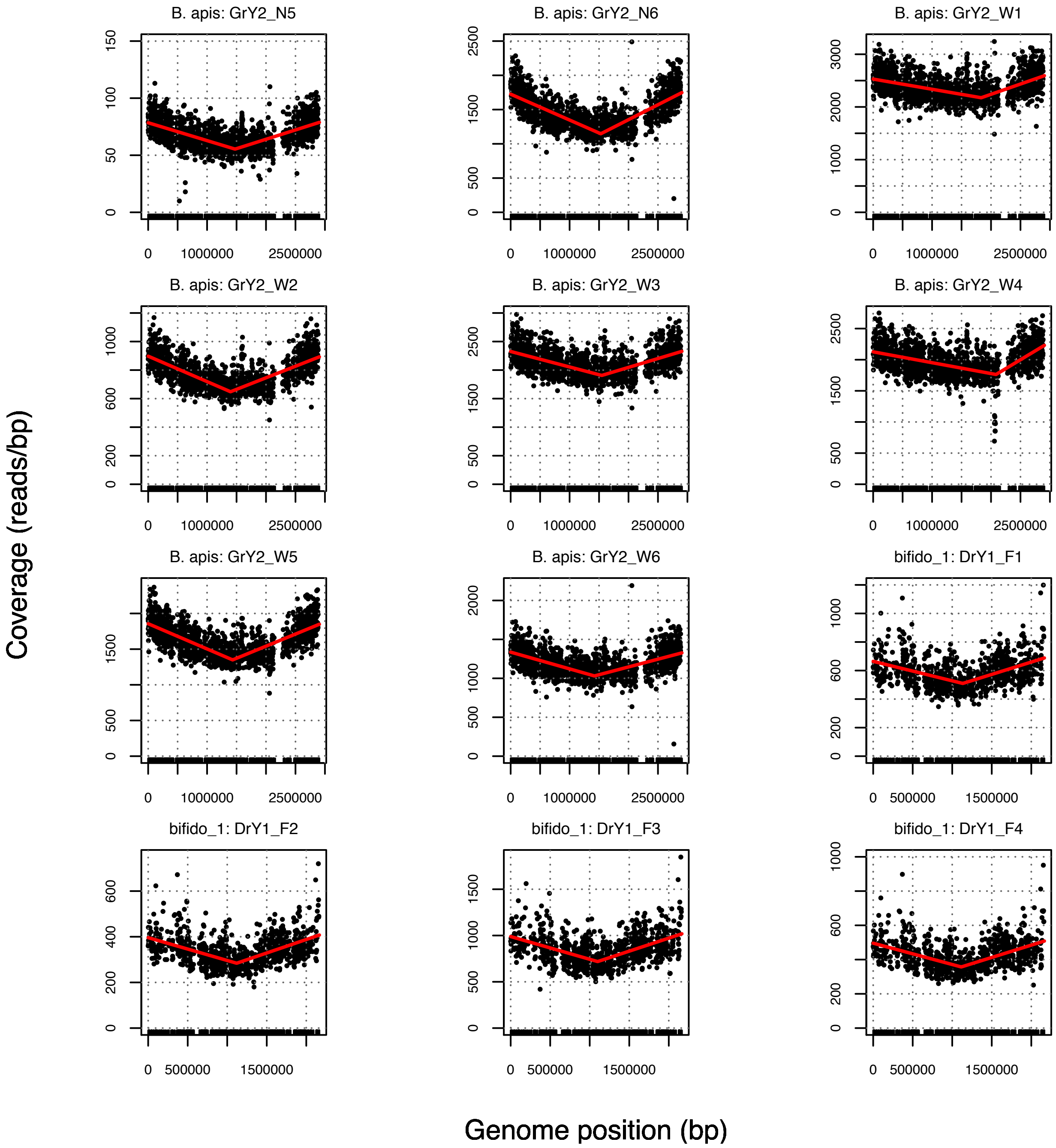
Coverage (reads/bp)
Genome position (bp)
B. apis: GrY2_N5
B. apis: GrY2_N6
B. apis: GrY2_W1
B. apis: GrY2_W2
B. apis: GrY2_W3
B. apis: GrY2_W4
B. apis: GrY2_W5
B. apis: GrY2_W6
bifido_1: DrY1_F1
bifido_1: DrY1_F2
bifido_1: DrY1_F3
bifido_1: DrY1_F4

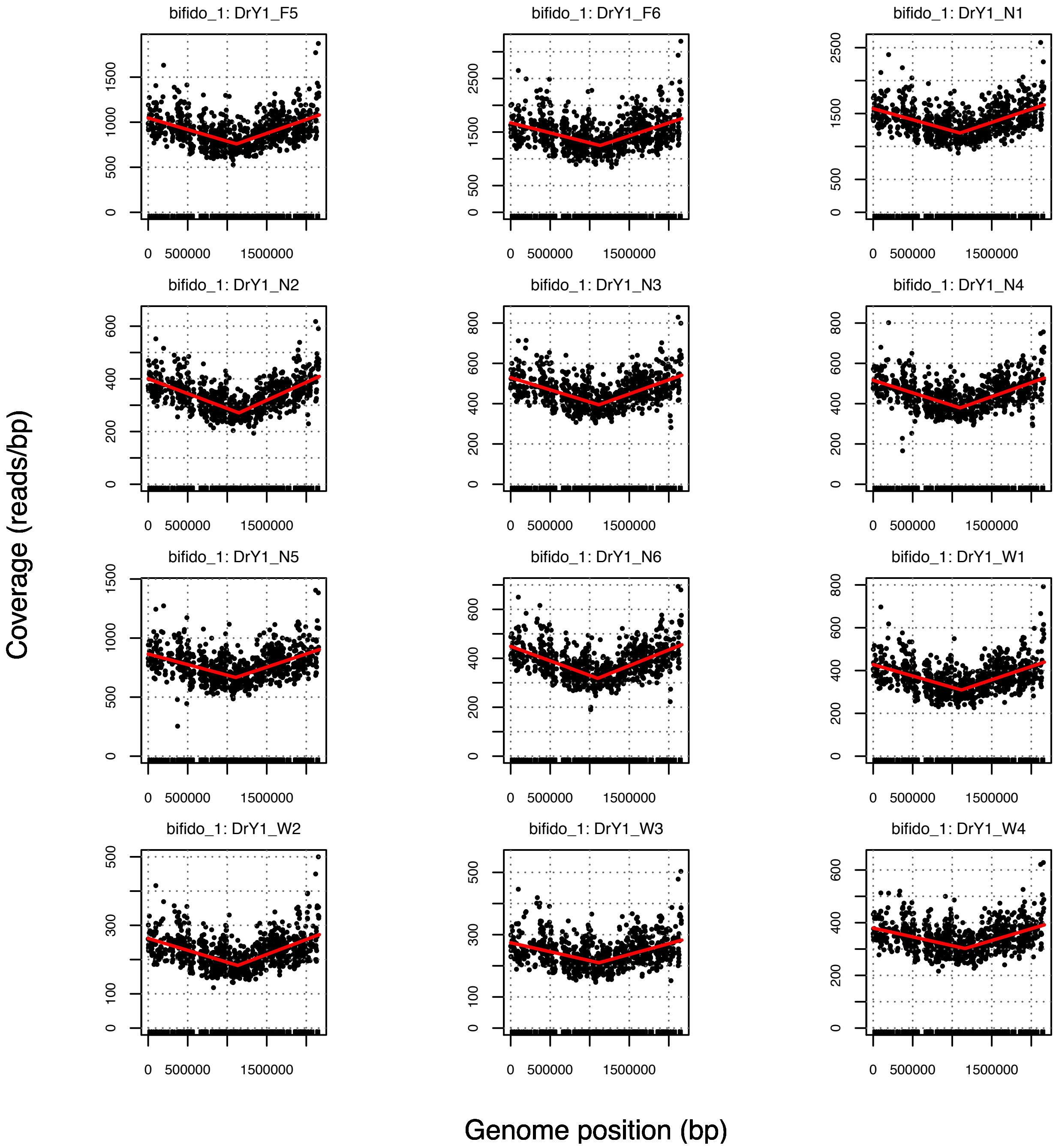
bifido_1: DrY1_F5
bifido_1: DrY1_F6
bifido_1: DrY1_N1
bifido_1: DrY1_N2
bifido_1: DrY1_N3
bifido_1: DrY1_N4
bifido_1: DrY1_N5
bifido_1: DrY1_N6
bifido_1: DrY1_W1
bifido_1: DrY1_W2
bifido_1: DrY1_W3
bifido_1: DrY1_W4
Coverage (reads/bp)
Genome position (bp)

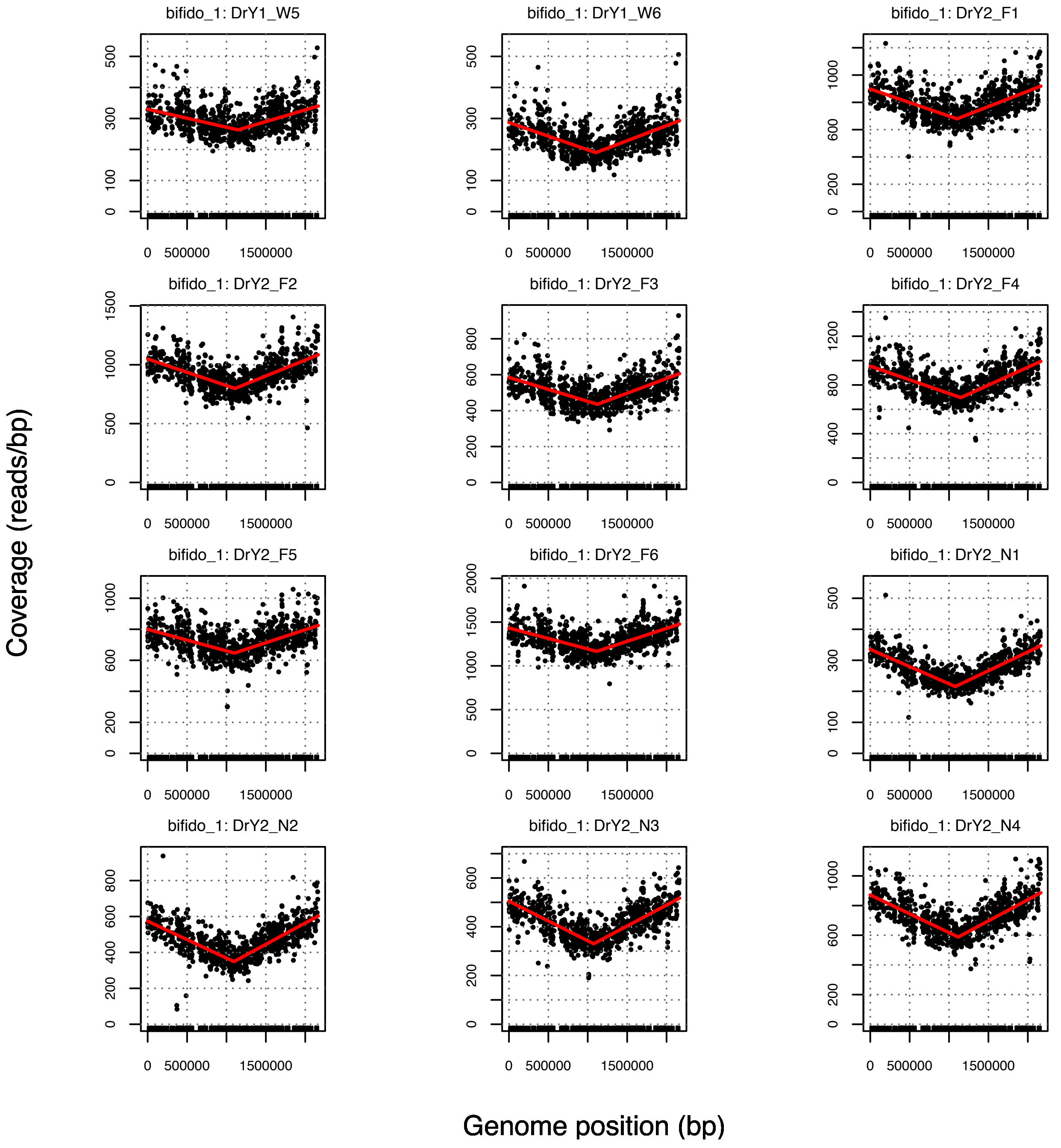
bifido_1: DrY1_W5
bifido_1: DrY1_W6
bifido_1: DrY2_F1
bifido_1: DrY2_F2
bifido_1: DrY2_F3
bifido_1: DrY2_F4
bifido_1: DrY2_F5
bifido_1: DrY2_F6
bifido_1: DrY2_N1
bifido_1: DrY2_N2
bifido_1: DrY2_N3
bifido_1: DrY2_N4
Coverage (reads/bp)
Genome position (bp)
0 500000 1500000

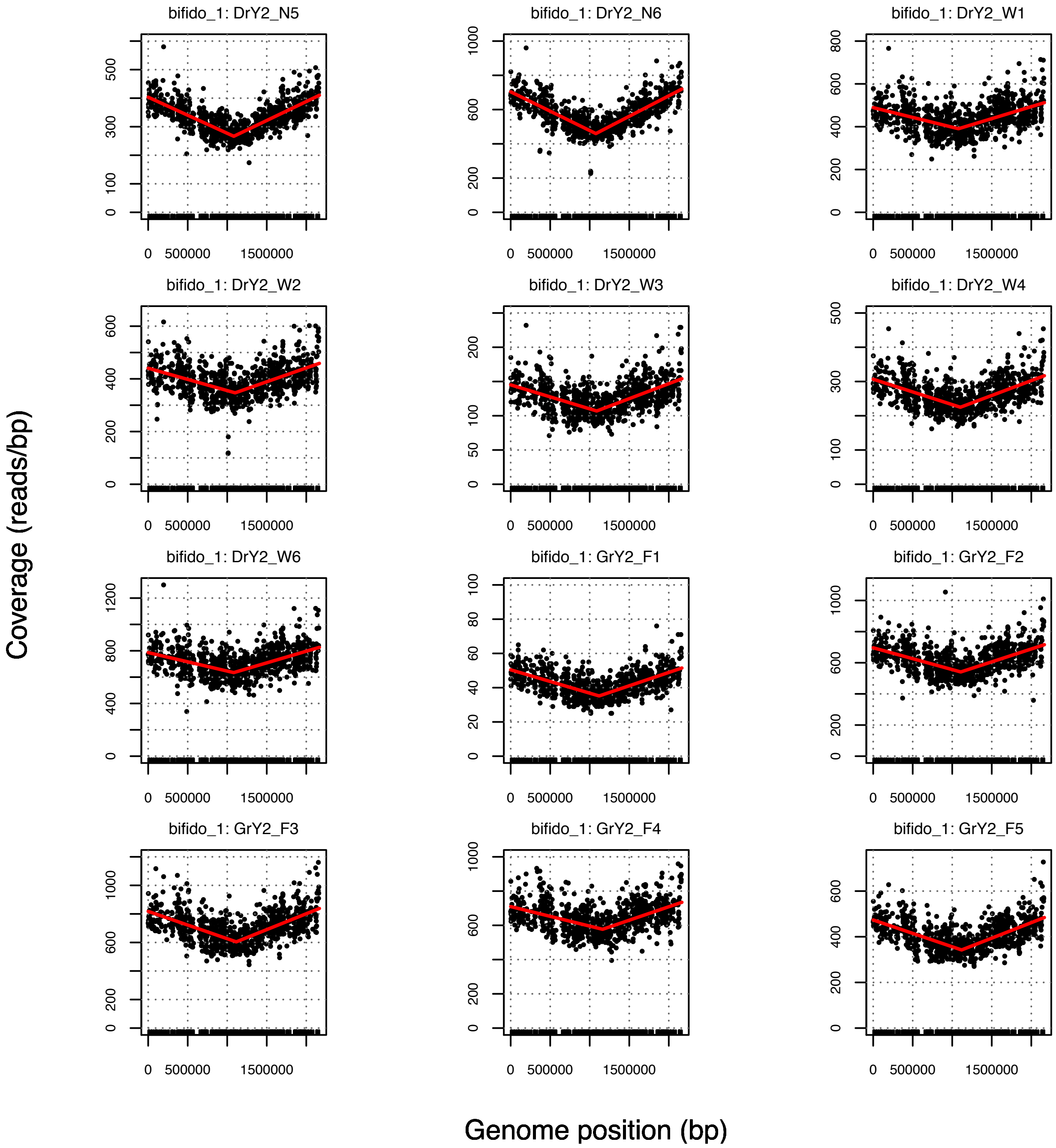
bifido_1: DrY2_N5
bifido_1: DrY2_N6
bifido_1: DrY2_W1
bifido_1: DrY2_W2
bifido_1: DrY2_W3
bifido_1: DrY2_W4
bifido_1: DrY2_W6
bifido_1: GrY2_F1
bifido_1: GrY2_F2
bifido_1: GrY2_F3
bifido_1: GrY2_F4
bifido_1: GrY2_F5
Coverage (reads/bp)
Genome position (bp)
0
500000
1500000

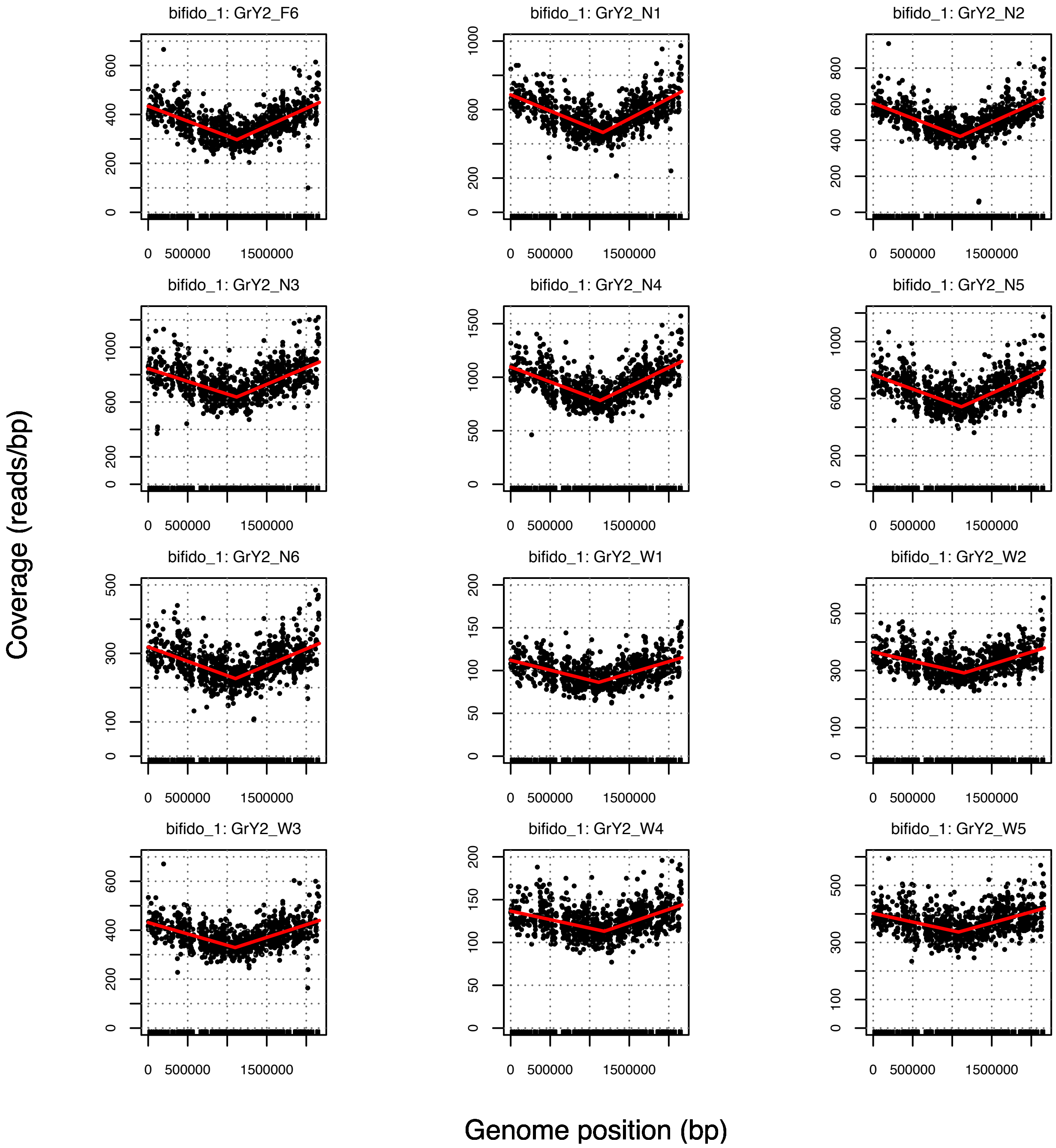
bifido_1: GrY2_F6
bifido_1: GrY2_N1
bifido_1: GrY2_N2
bifido_1: GrY2_N3
bifido_1: GrY2_N4
bifido_1: GrY2_N5
bifido_1: GrY2_N6
bifido_1: GrY2_W1
bifido_1: GrY2_W2
bifido_1: GrY2_W3
bifido_1: GrY2_W4
bifido_1: GrY2_W5
0 500000 1500000
Coverage (reads/bp)
Genome position (bp)

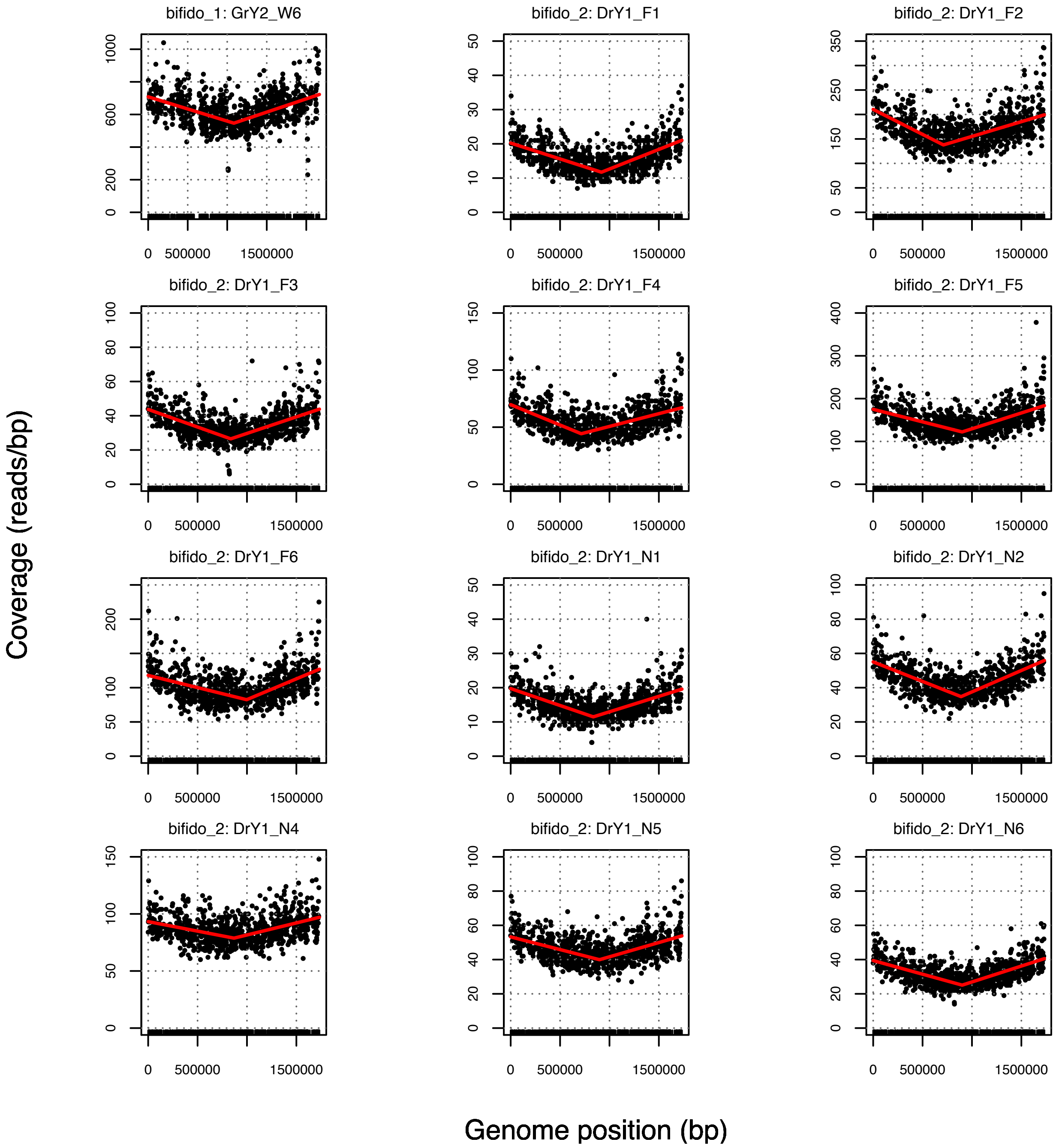
bifido_1: GrY2_W6
bifido_2: DrY1_F1
bifido_2: DrY1_F2
bifido_2: DrY1_F3
bifido_2: DrY1_F4
bifido_2: DrY1_F5
bifido_2: DrY1_F6
bifido_2: DrY1_N1
bifido_2: DrY1_N2
bifido_2: DrY1_N4
bifido_2: DrY1_N5
bifido_2: DrY1_N6
Coverage (reads/bp)
Genome position (bp)

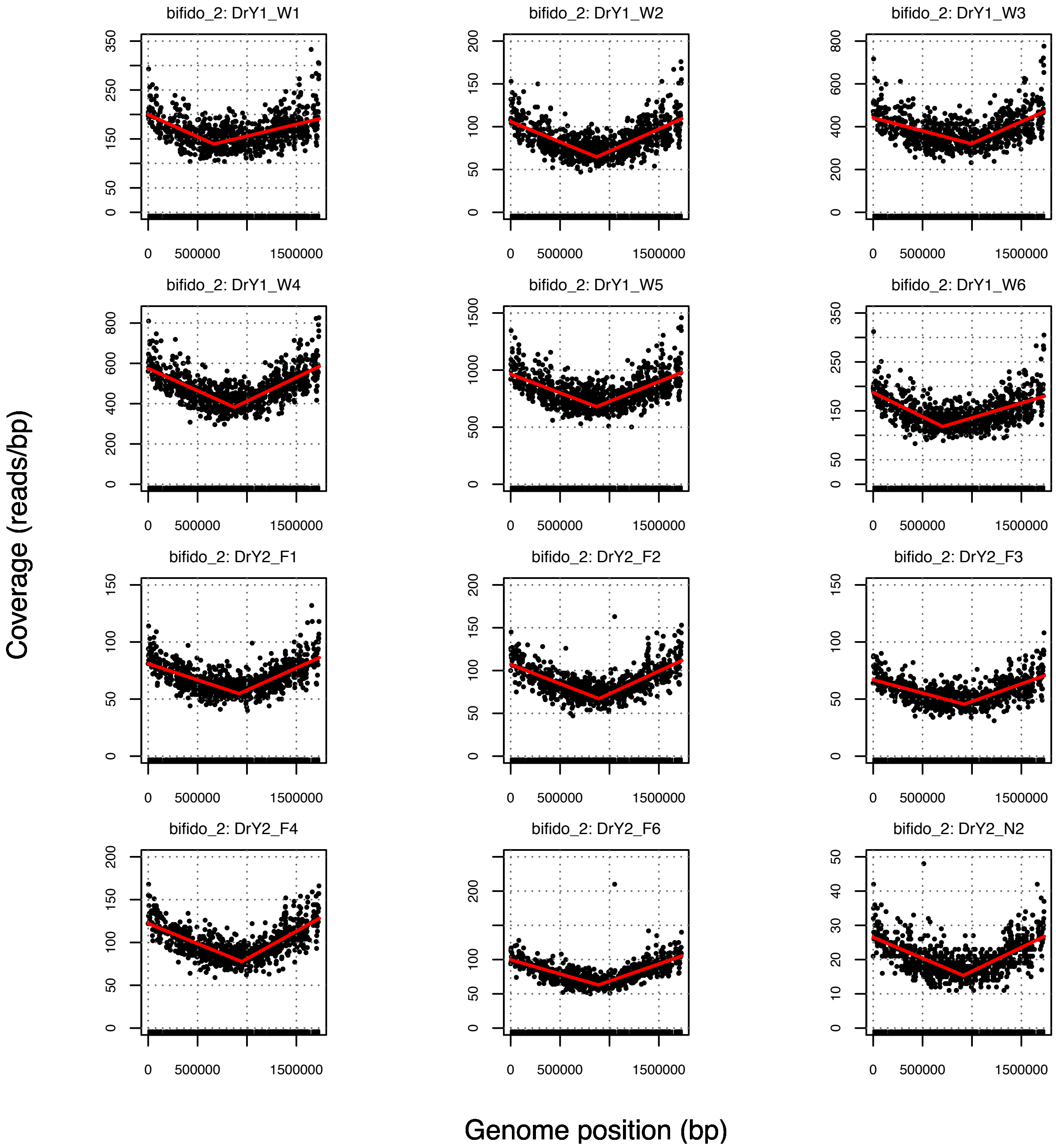
bifido_2: DrY1_W1
bifido_2: DrY1_W2
bifido_2: DrY1_W3
bifido_2: DrY1_W4
bifido_2: DrY1_W5
bifido_2: DrY1_W6
bifido_2: DrY2_F1
bifido_2: DrY2_F2
bifido_2: DrY2_F3
bifido_2: DrY2_F4
bifido_2: DrY2_F6
bifido_2: DrY2_N2
Coverage (reads/bp)
Genome position (bp)

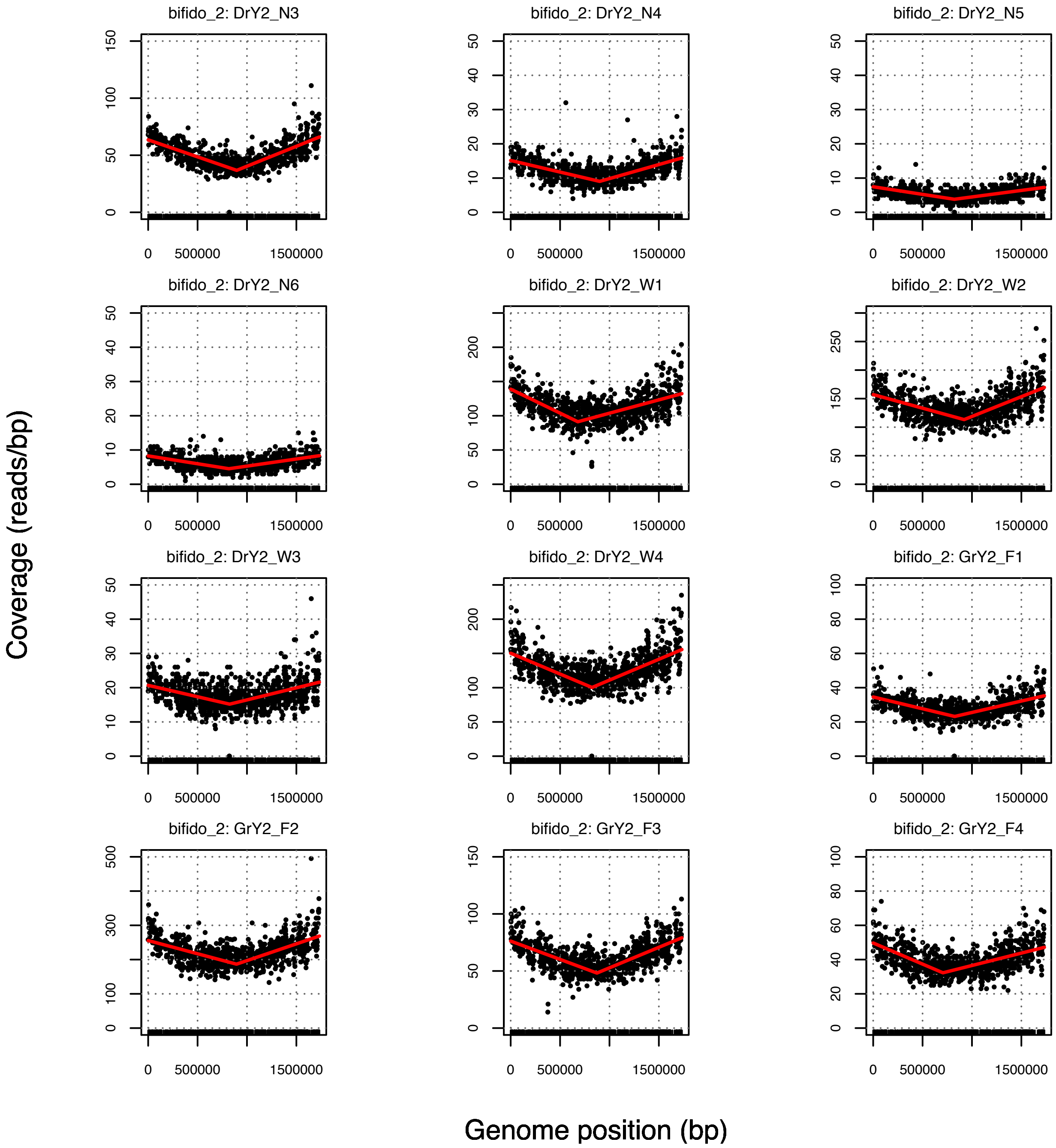
bifido_2: DrY2_N3
bifido_2: DrY2_N4
bifido_2: DrY2_N5
bifido_2: DrY2_N6
bifido_2: DrY2_W1
bifido_2: DrY2_W2
bifido_2: DrY2_W3
bifido_2: DrY2_W4
bifido_2: GrY2_F1
bifido_2: GrY2_F2
bifido_2: GrY2_F3
bifido_2: GrY2_F4
Coverage (reads/bp)
Genome position (bp)

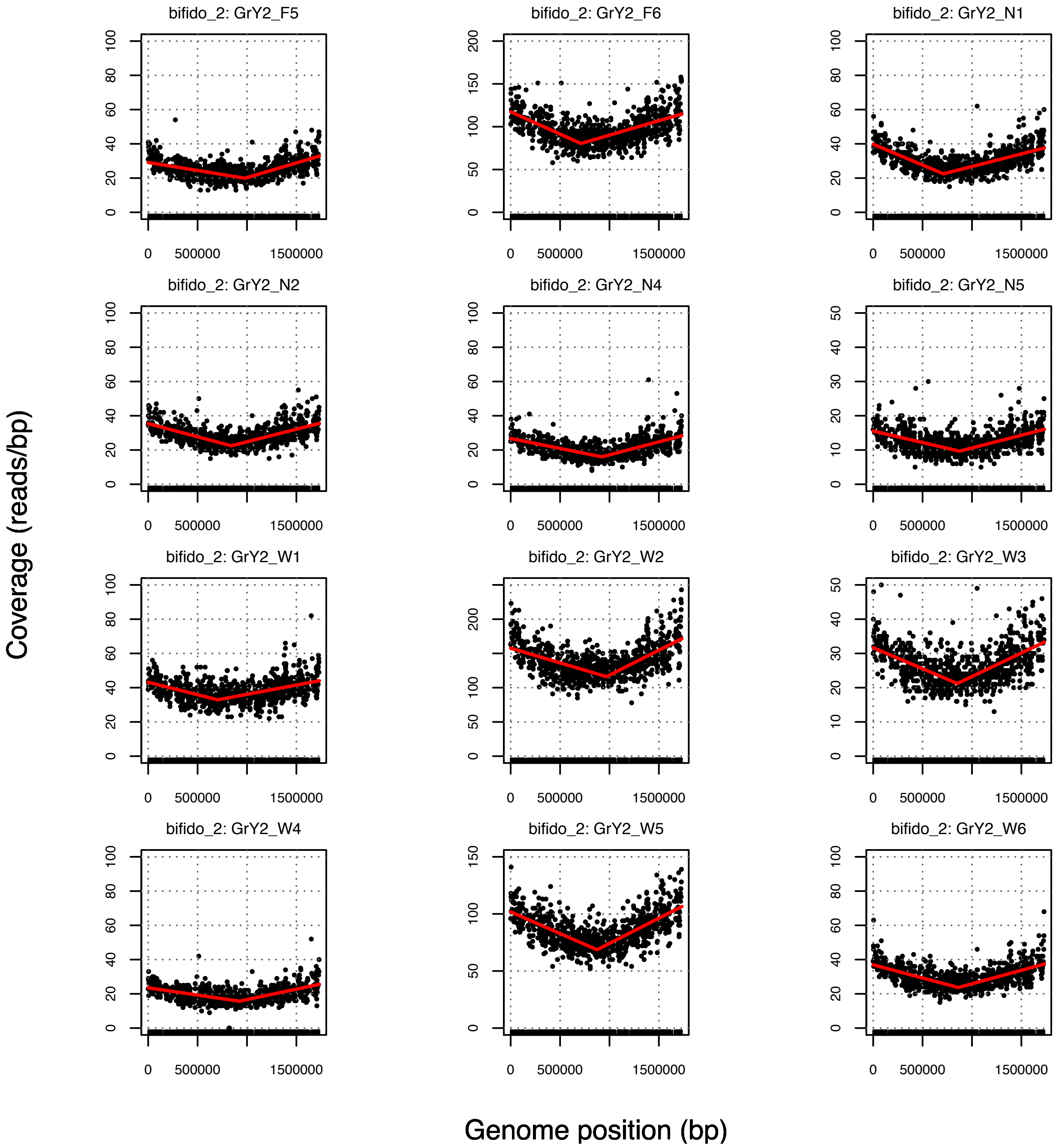
bifido_2: GrY2_F5
bifido_2: GrY2_F6
bifido_2: GrY2_N1
bifido_2: GrY2_N2
bifido_2: GrY2_N4
bifido_2: GrY2_N5
bifido_2: GrY2_W1
bifido_2: GrY2_W2
bifido_2: GrY2_W3
bifido_2: GrY2_W4
bifido_2: GrY2_W5
bifido_2: GrY2_W6
Coverage (reads/bp)
Genome position (bp)

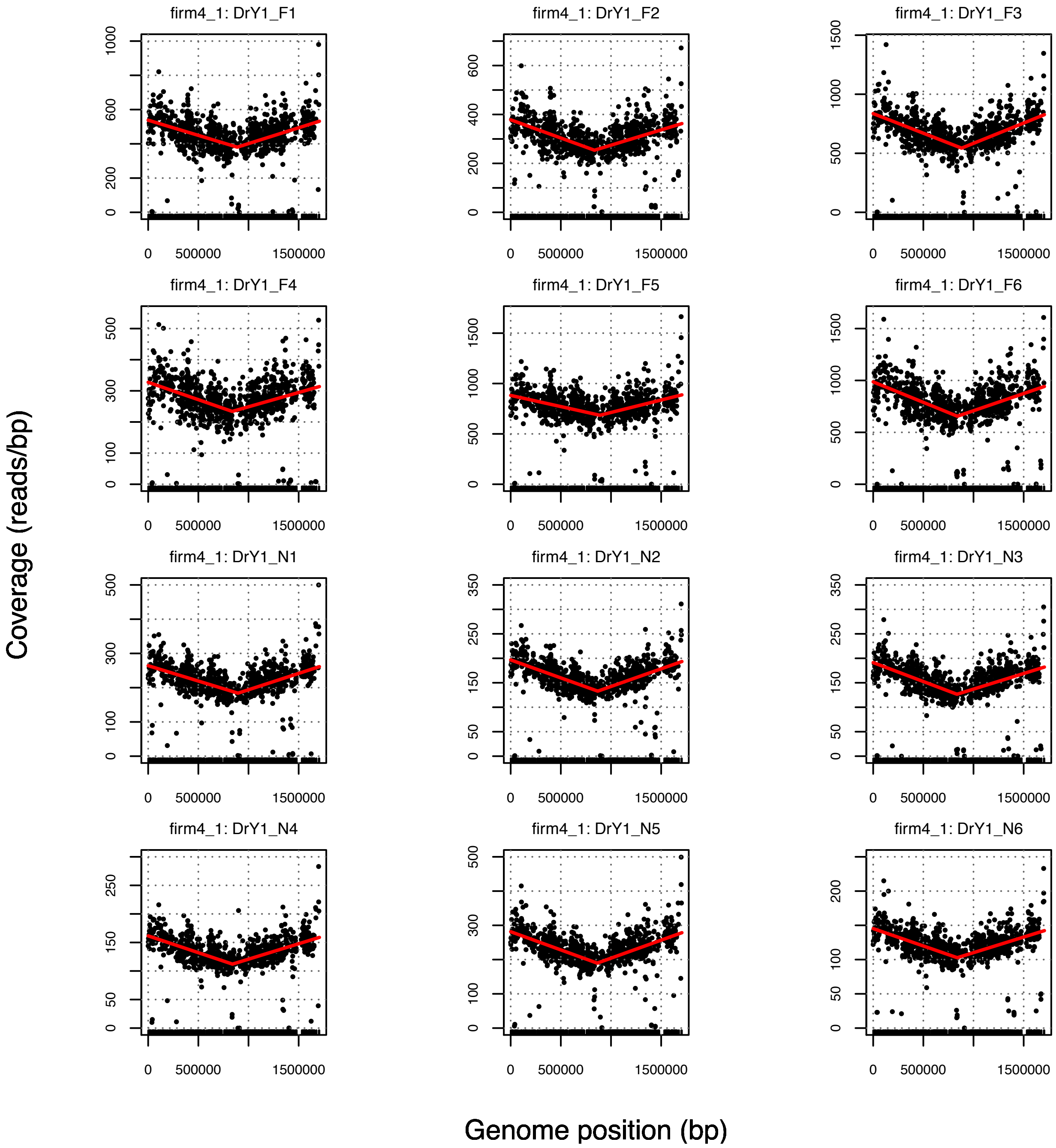
firm4_1: DrY1_F1
firm4_1: DrY1_F2
firm4_1: DrY1_F3
firm4_1: DrY1_F4
firm4_1: DrY1_F5
firm4_1: DrY1_F6
firm4_1: DrY1_N1
firm4_1: DrY1_N2
firm4_1: DrY1_N3
firm4_1: DrY1_N4
firm4_1: DrY1_N5
firm4_1: DrY1_N6
Coverage (reads/bp)
Genome position (bp)
0
500000
1500000

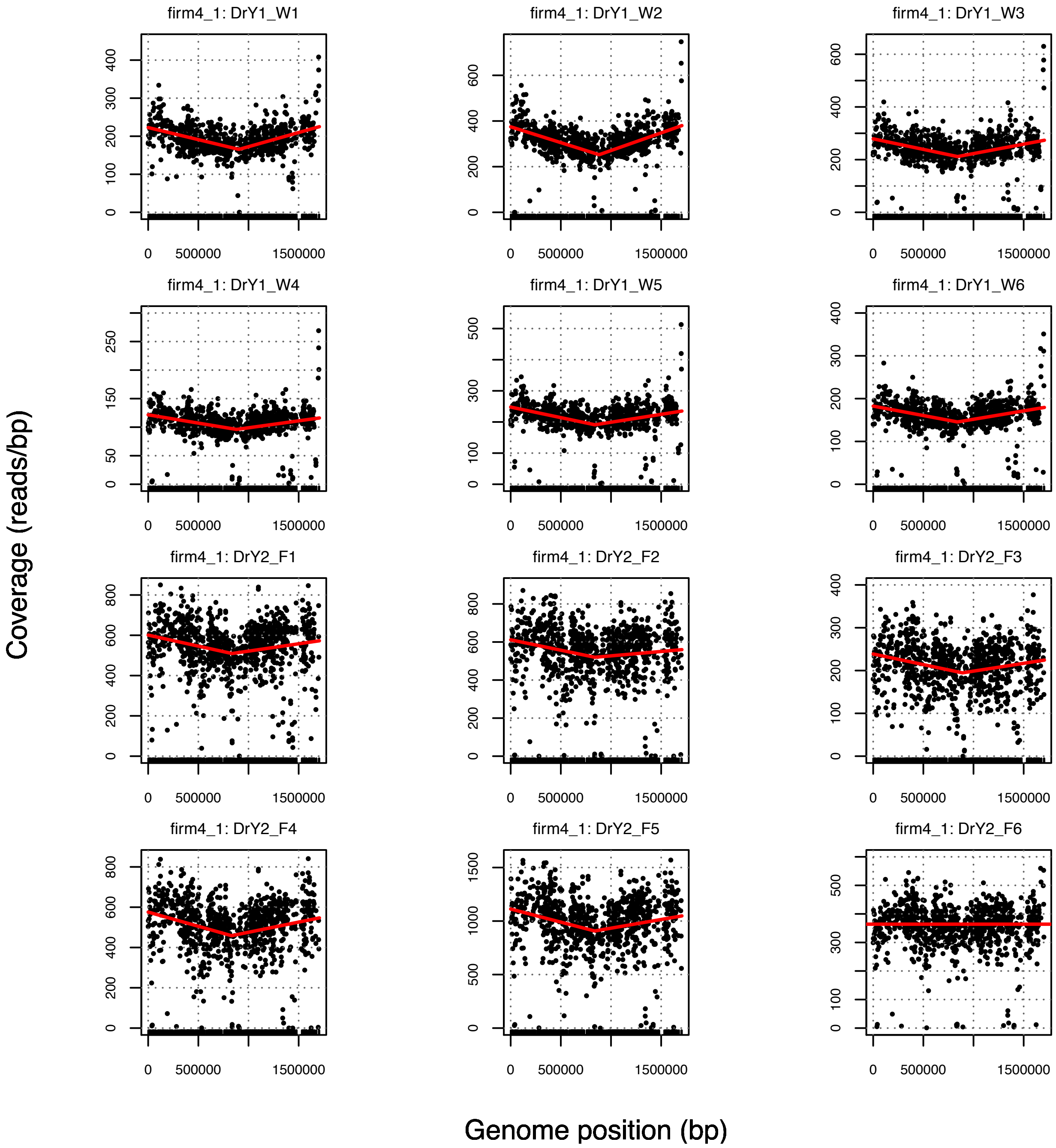
firm4_1: DrY1_W1
firm4_1: DrY1_W2
firm4_1: DrY1_W3
firm4_1: DrY1_W4
firm4_1: DrY1_W5
firm4_1: DrY1_W6
firm4_1: DrY2_F1
firm4_1: DrY2_F2
firm4_1: DrY2_F3
firm4_1: DrY2_F4
firm4_1: DrY2_F5
firm4_1: DrY2_F6
Coverage (reads/bp)
Genome position (bp)
0
500000
1500000

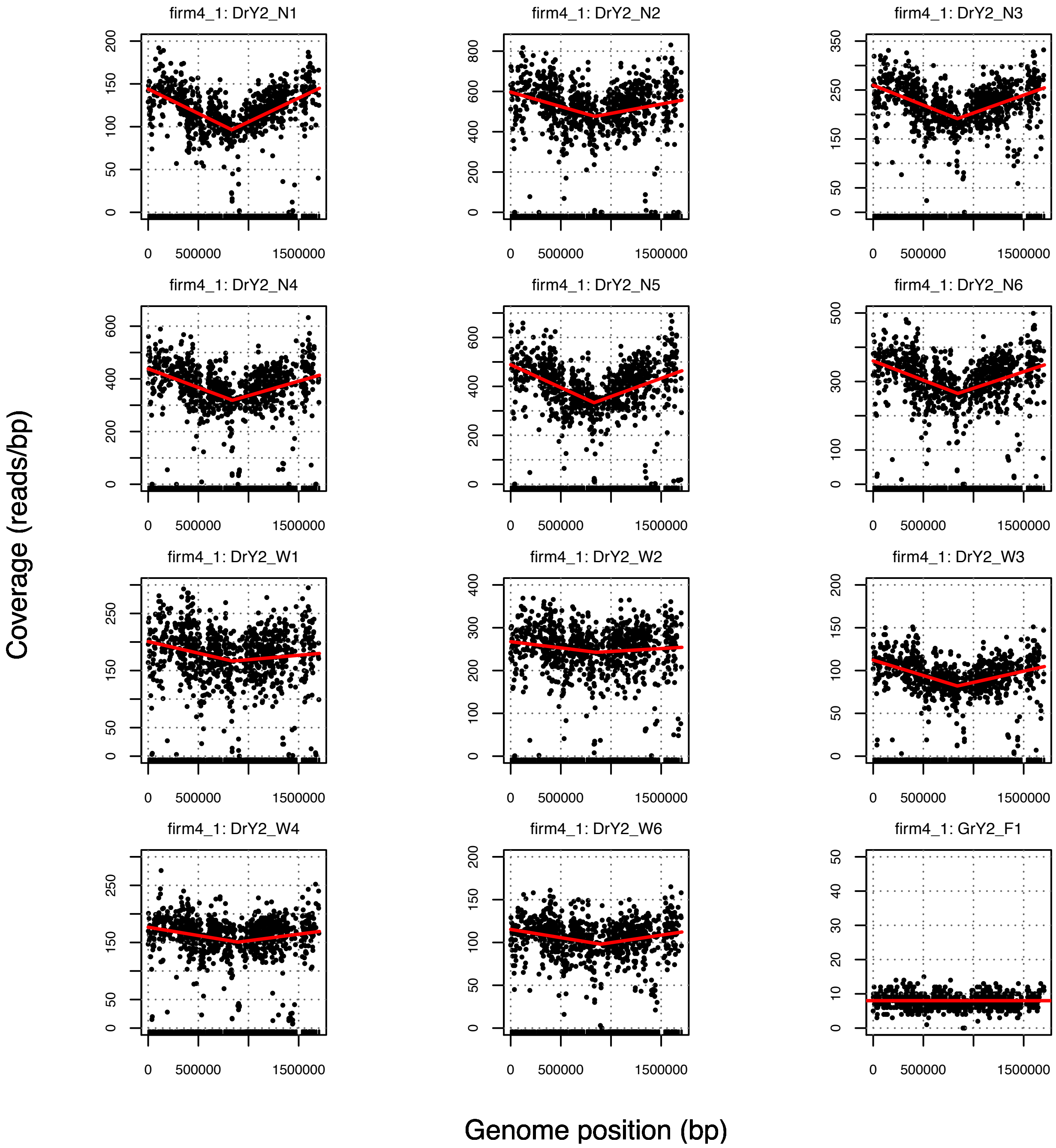
firm4_1: DrY2_N1
firm4_1: DrY2_N2
firm4_1: DrY2_N3
firm4_1: DrY2_N4
firm4_1: DrY2_N5
firm4_1: DrY2_N6
firm4_1: DrY2_W1
firm4_1: DrY2_W2
firm4_1: DrY2_W3
firm4_1: DrY2_W4
firm4_1: DrY2_W6
firm4_1: GrY2_F1
0
500000
1500000
Coverage (reads/bp)
Genome position (bp)

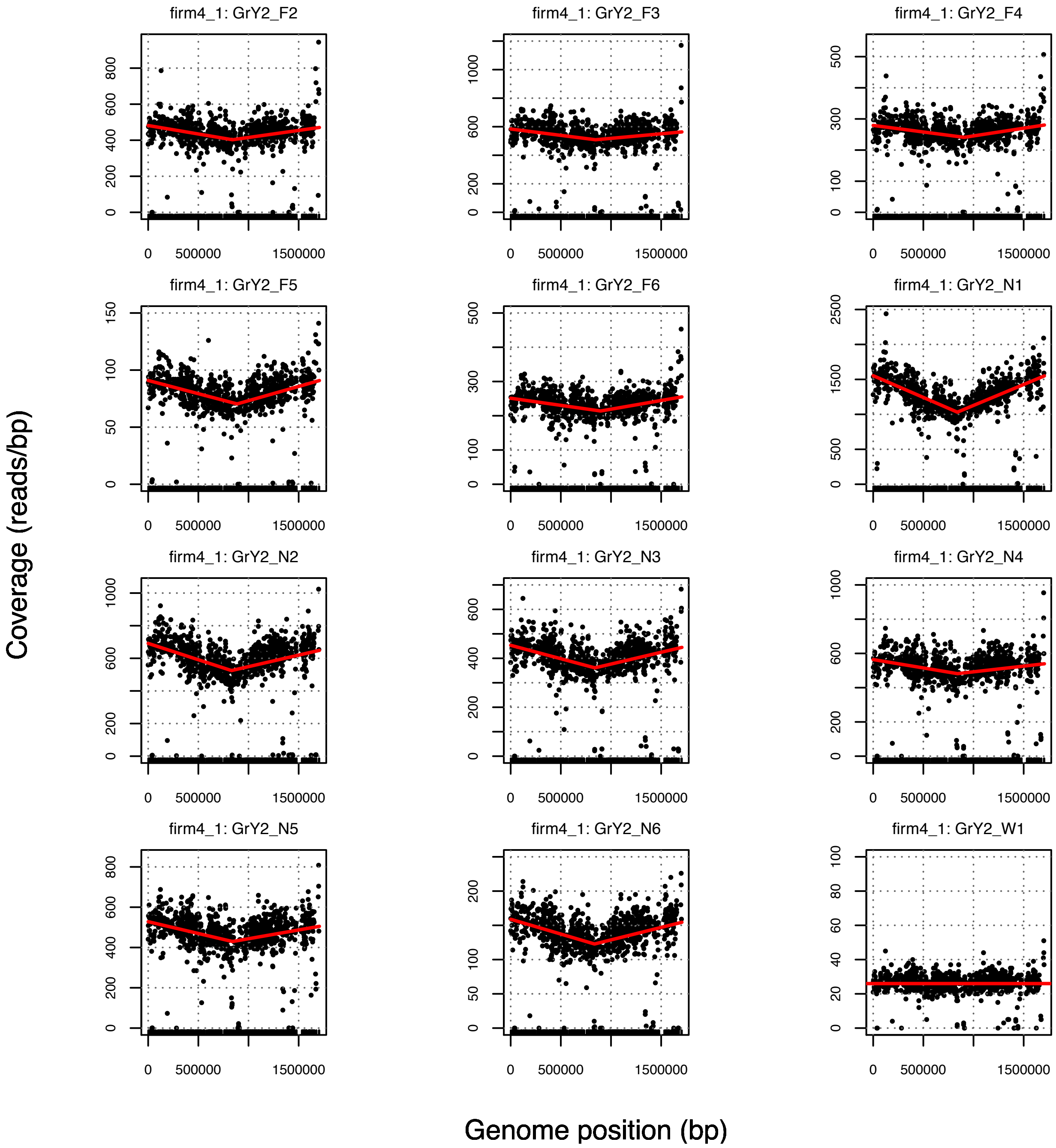
firm4_1: GrY2_F2
firm4_1: GrY2_F3
firm4_1: GrY2_F4
firm4_1: GrY2_F5
firm4_1: GrY2_F6
firm4_1: GrY2_N1
firm4_1: GrY2_N2
firm4_1: GrY2_N3
firm4_1: GrY2_N4
firm4_1: GrY2_N5
firm4_1: GrY2_N6
firm4_1: GrY2_W1
Coverage (reads/bp)
Genome position (bp)
0
500000
1500000

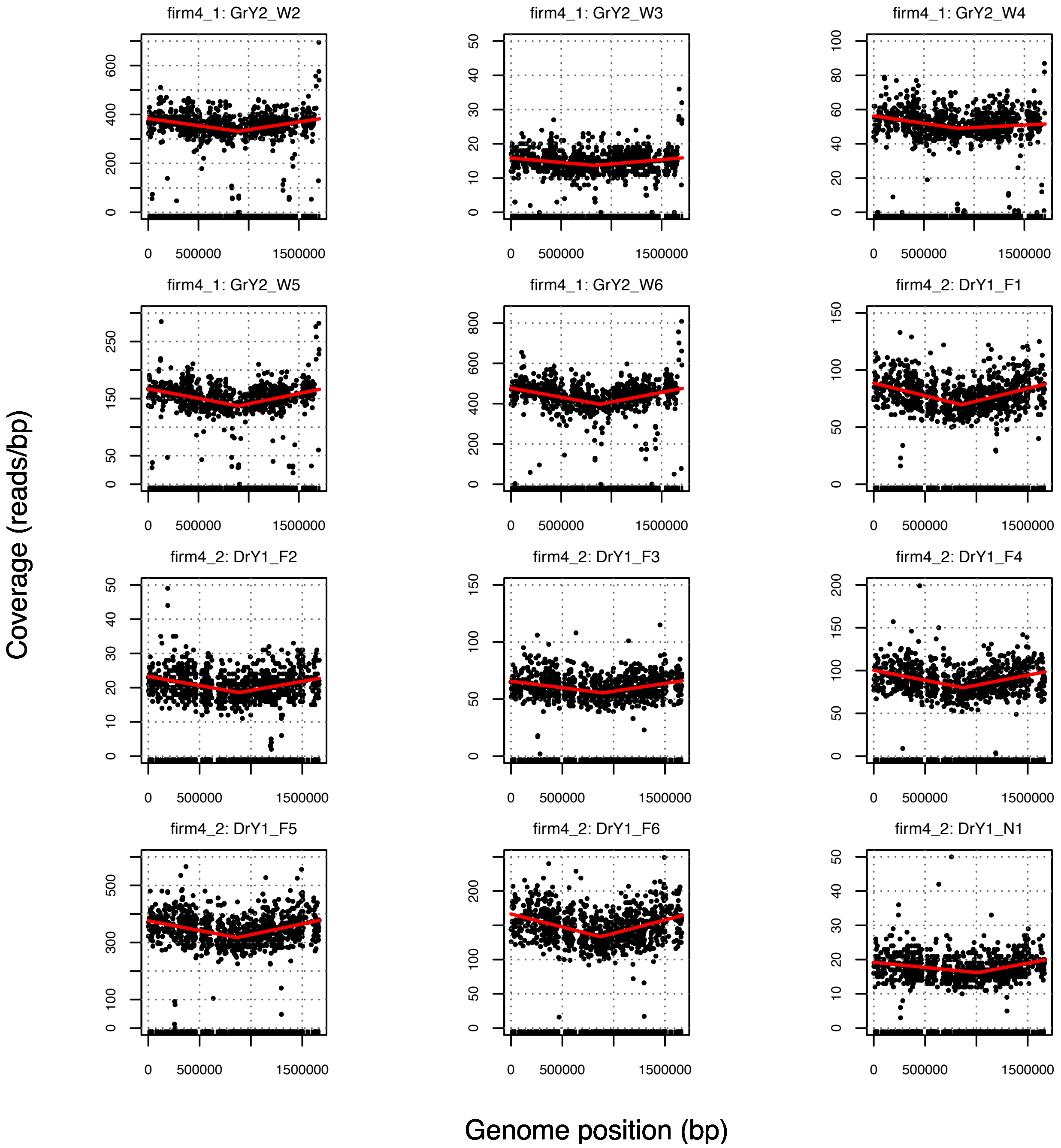
firm4_1: GrY2_W2
firm4_1: GrY2_W3
firm4_1: GrY2_W4
firm4_1: GrY2_W5
firm4_1: GrY2_W6
firm4_2: DrY1_F1
firm4_2: DrY1_F2
firm4_2: DrY1_F3
firm4_2: DrY1_F4
firm4_2: DrY1_F5
firm4_2: DrY1_F6
firm4_2: DrY1_N1
Coverage (reads/bp)
Genome position (bp)

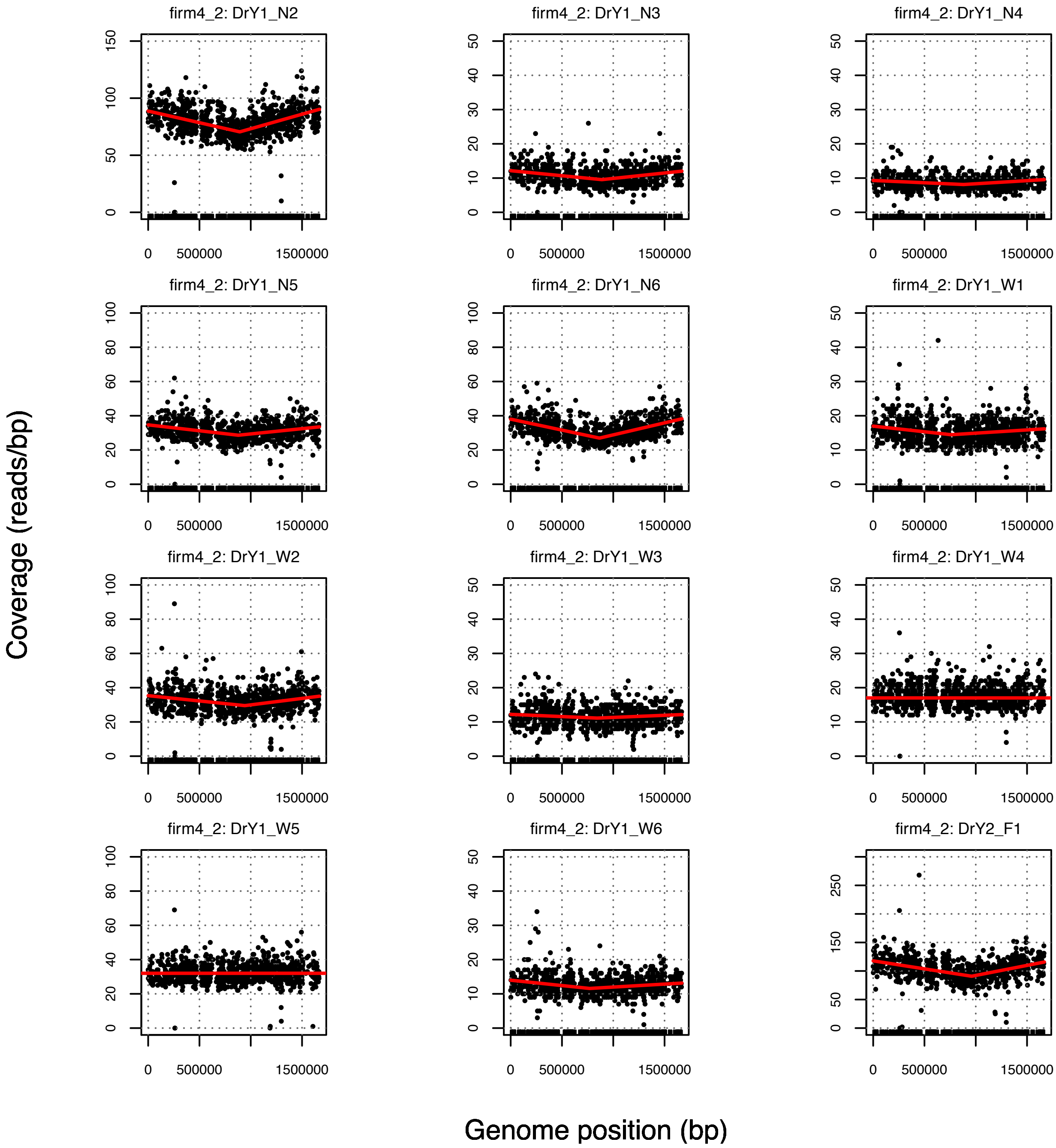
firm4_2: DrY1_N2
firm4_2: DrY1_N3
firm4_2: DrY1_N4
firm4_2: DrY1_N5
firm4_2: DrY1_N6
firm4_2: DrY1_W1
firm4_2: DrY1_W2
firm4_2: DrY1_W3
firm4_2: DrY1_W4
firm4_2: DrY1_W5
firm4_2: DrY1_W6
firm4_2: DrY2_F1
Coverage (reads/bp)
Genome position (bp)

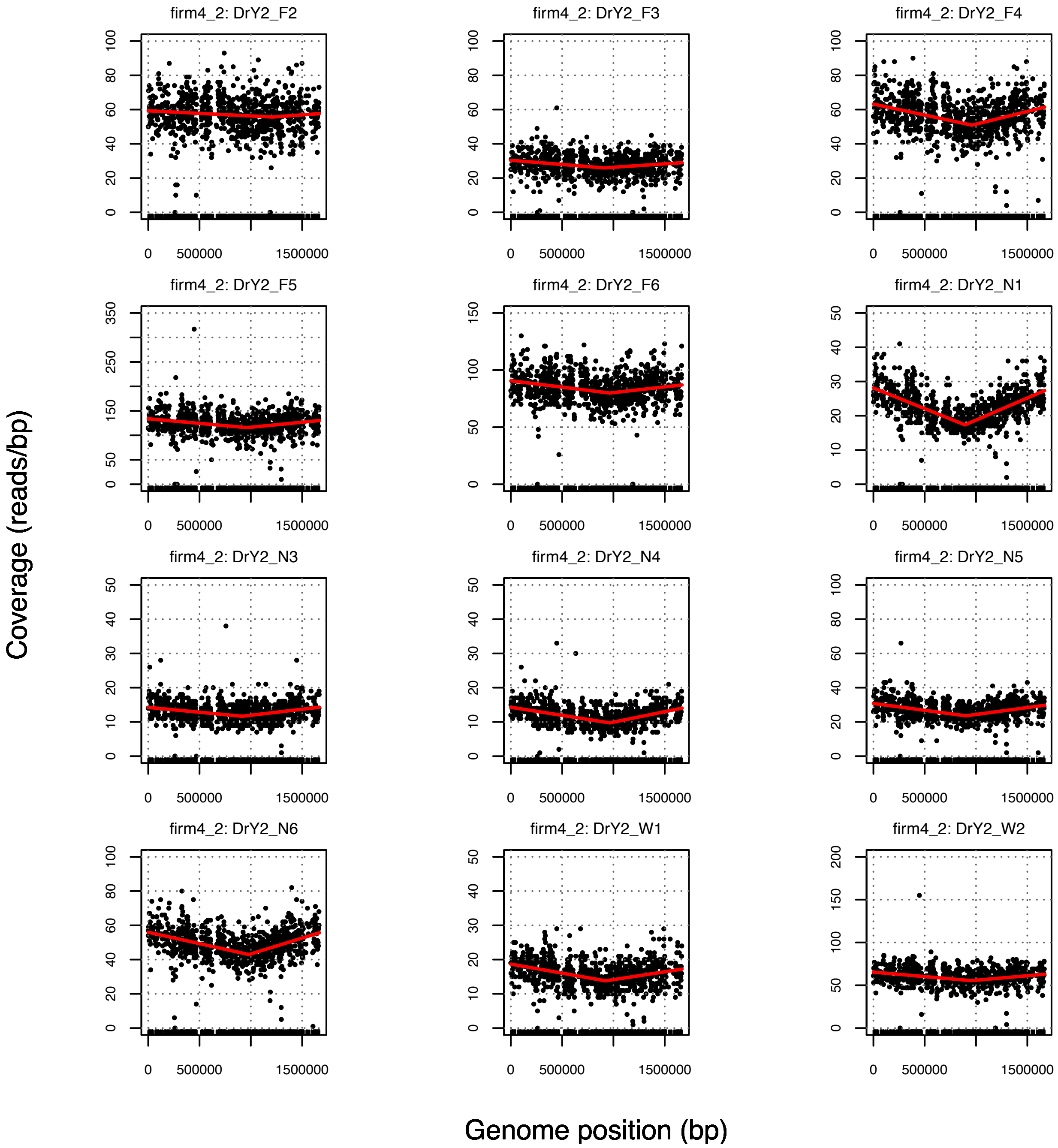
firm4_2: DrY2_F2
firm4_2: DrY2_F3
firm4_2: DrY2_F4
firm4_2: DrY2_F5
firm4_2: DrY2_F6
firm4_2: DrY2_N1
firm4_2: DrY2_N3
firm4_2: DrY2_N4
firm4_2: DrY2_N5
firm4_2: DrY2_N6
firm4_2: DrY2_W1
firm4_2: DrY2_W2
Coverage (reads/bp)
Genome position (bp)

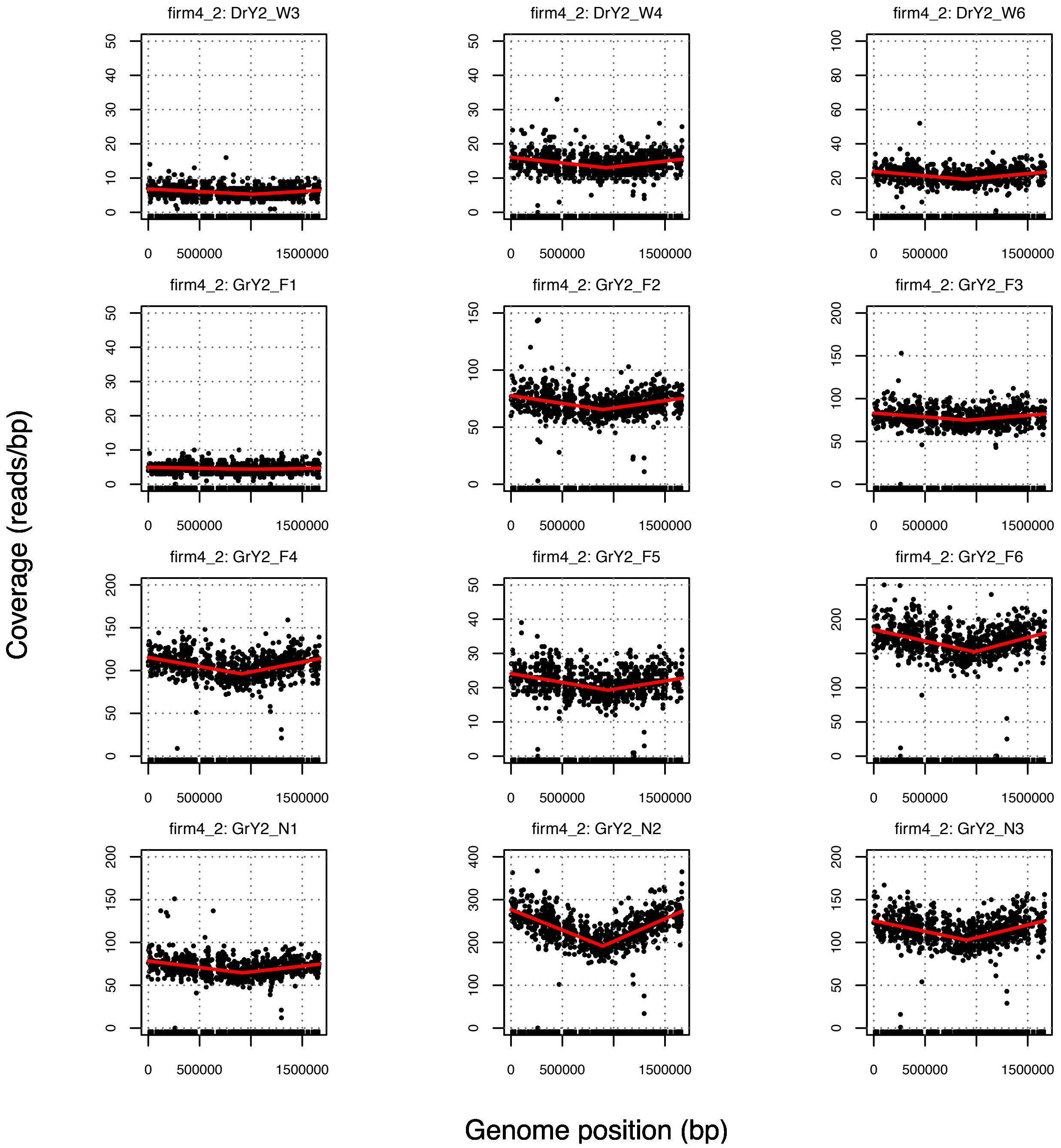

firm4_2: DrY2_W3
firm4_2: DrY2_W4
firm4_2: DrY2_W6
firm4_2: GrY2_F1
firm4_2: GrY2_F2
firm4_2: GrY2_F3
firm4_2: GrY2_F4
firm4_2: GrY2_F5
firm4_2: GrY2_F6
firm4_2: GrY2_N1
firm4_2: GrY2_N2
firm4_2: GrY2_N3
Coverage (reads/bp)
Genome position (bp)
0
500000
1500000

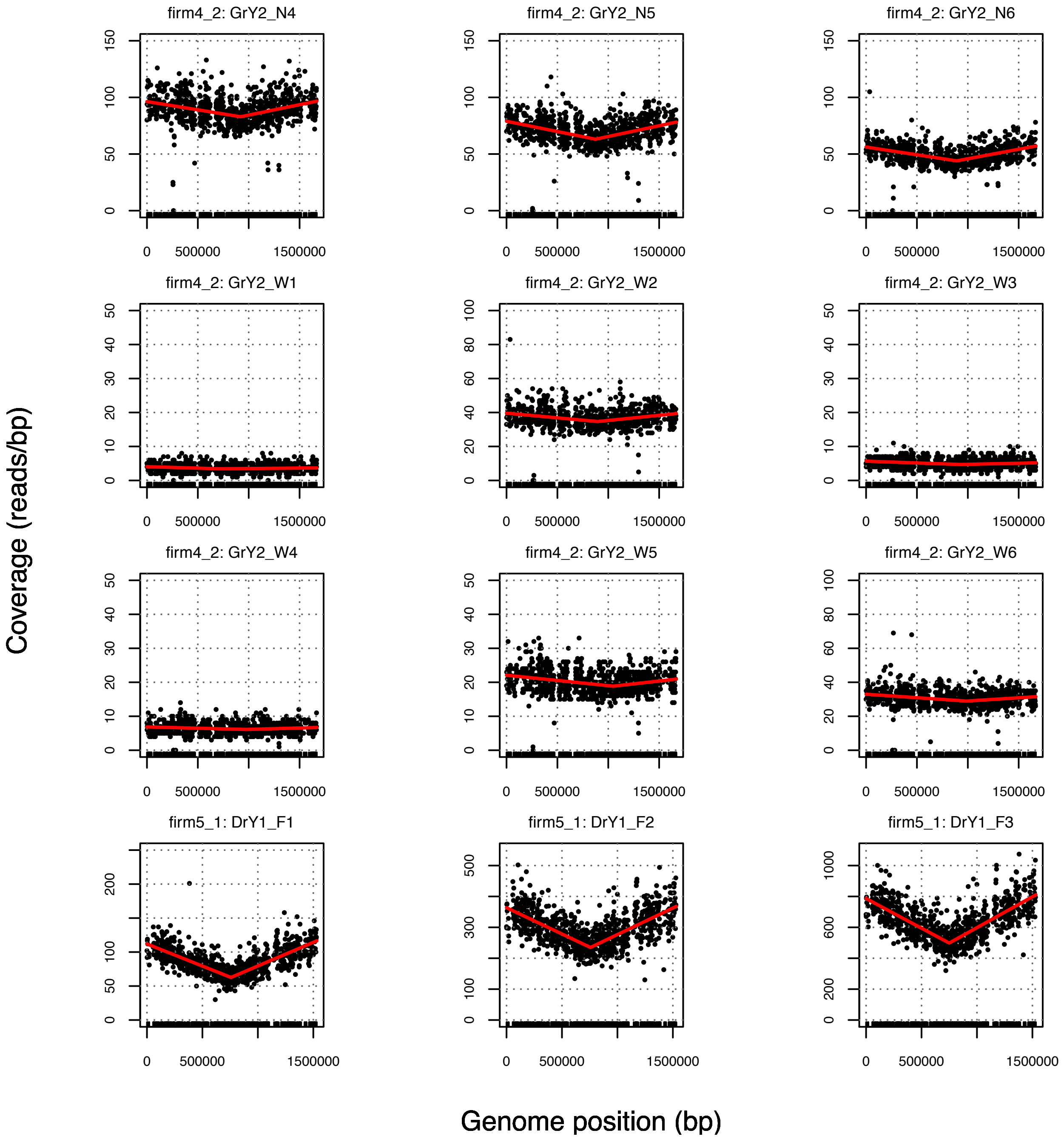
firm4_2: GrY2_N4
firm4_2: GrY2_N5
firm4_2: GrY2_N6
firm4_2: GrY2_W1
firm4_2: GrY2_W2
firm4_2: GrY2_W3
firm4_2: GrY2_W4
firm4_2: GrY2_W5
firm4_2: GrY2_W6
firm5_1: DrY1_F1
firm5_1: DrY1_F2
firm5_1: DrY1_F3
Coverage (reads/bp)
Genome position (bp)

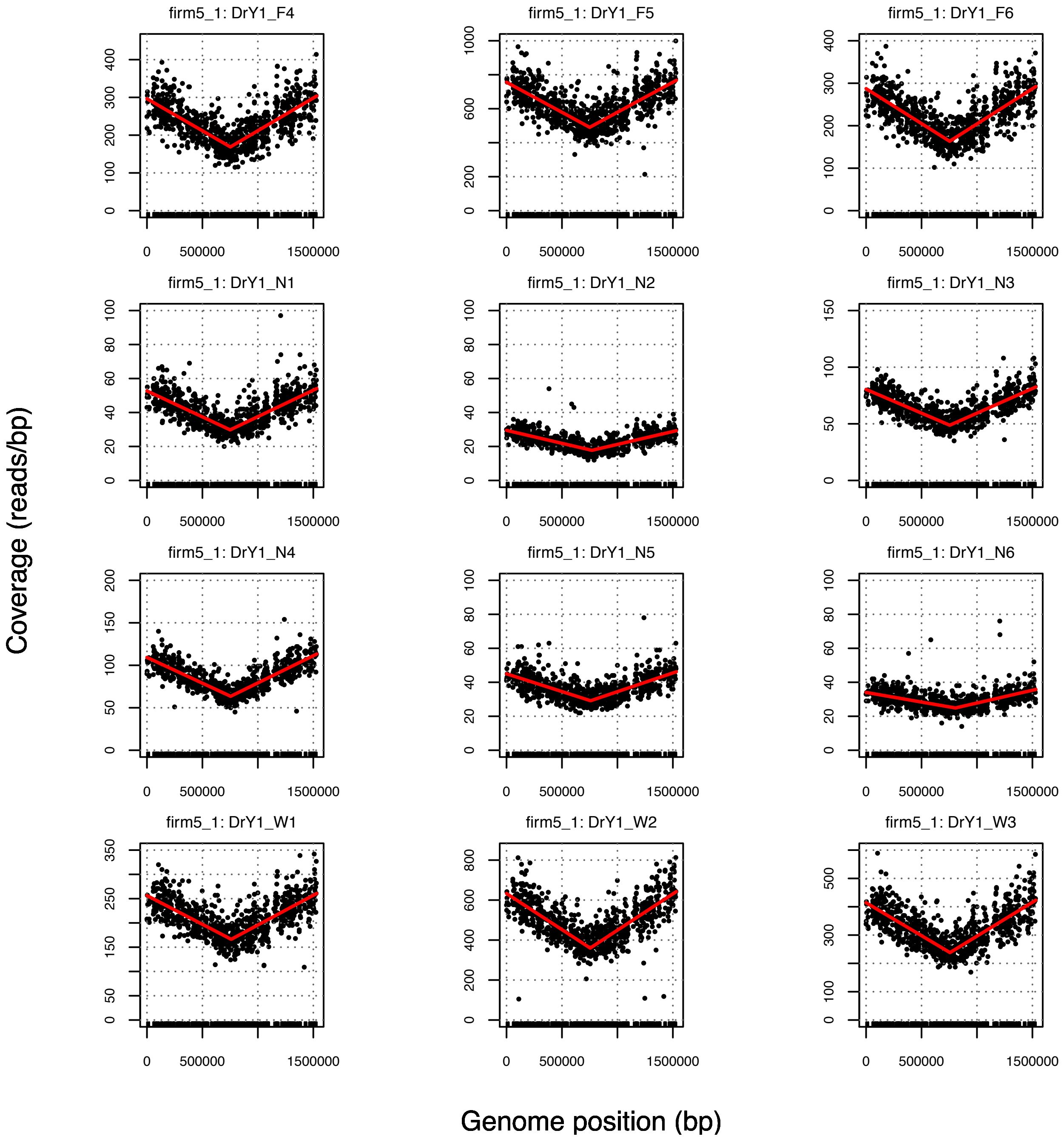

firm5_1: DrY1_F4
firm5_1: DrY1_F5
firm5_1: DrY1_F6
firm5_1: DrY1_N1
firm5_1: DrY1_N2
firm5_1: DrY1_N3
firm5_1: DrY1_N4
firm5_1: DrY1_N5
firm5_1: DrY1_N6
firm5_1: DrY1_W1
firm5_1: DrY1_W2
firm5_1: DrY1_W3
Coverage (reads/bp)
Genome position (bp)
0
500000
1500000

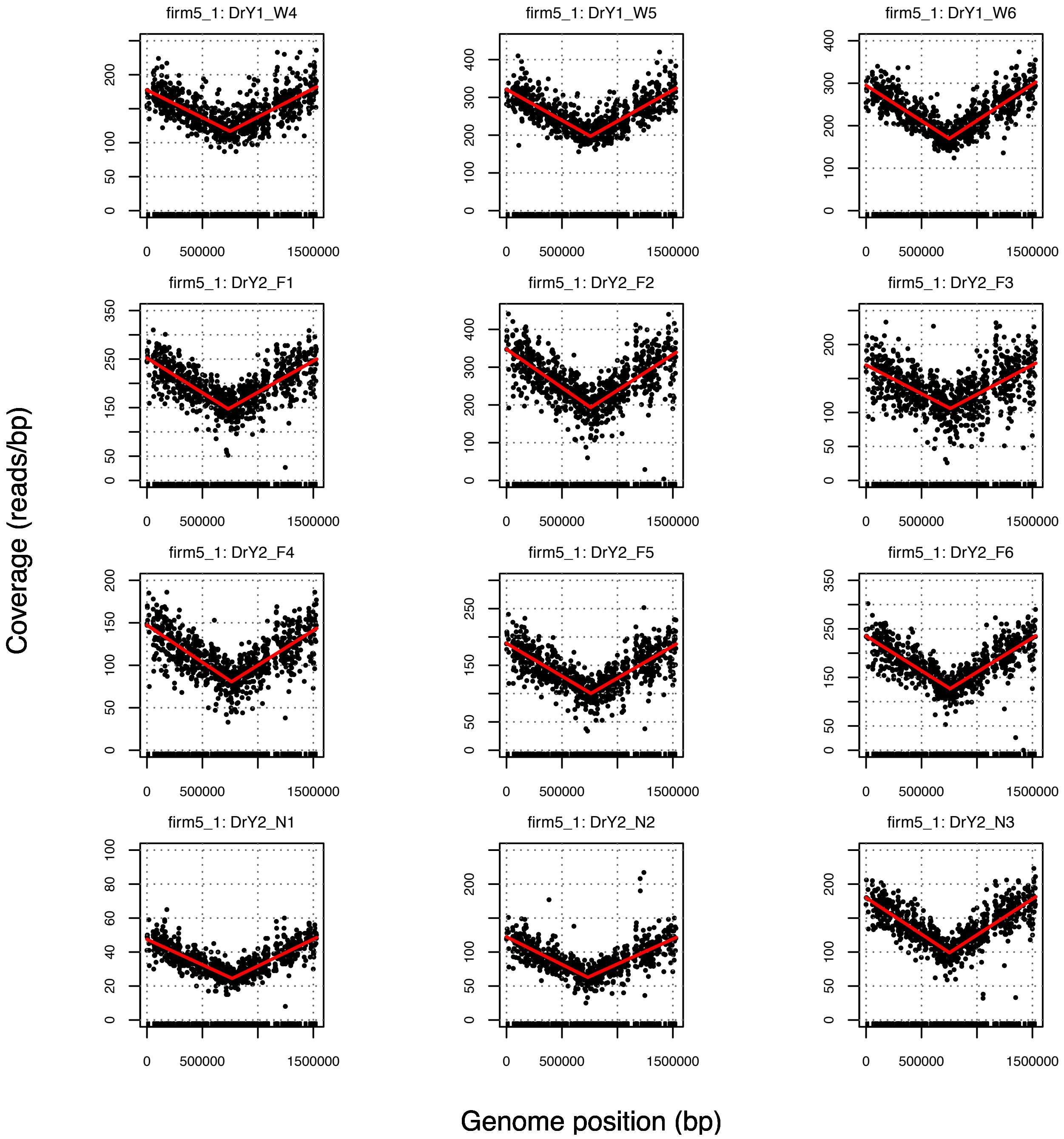
firm5_1: DrY1_W4
firm5_1: DrY1_W5
firm5_1: DrY1_W6
firm5_1: DrY2_F1
firm5_1: DrY2_F2
firm5_1: DrY2_F3
firm5_1: DrY2_F4
firm5_1: DrY2_F5
firm5_1: DrY2_F6
firm5_1: DrY2_N1
firm5_1: DrY2_N2
firm5_1: DrY2_N3
0
500000
1500000
Coverage (reads/bp)
Genome position (bp)

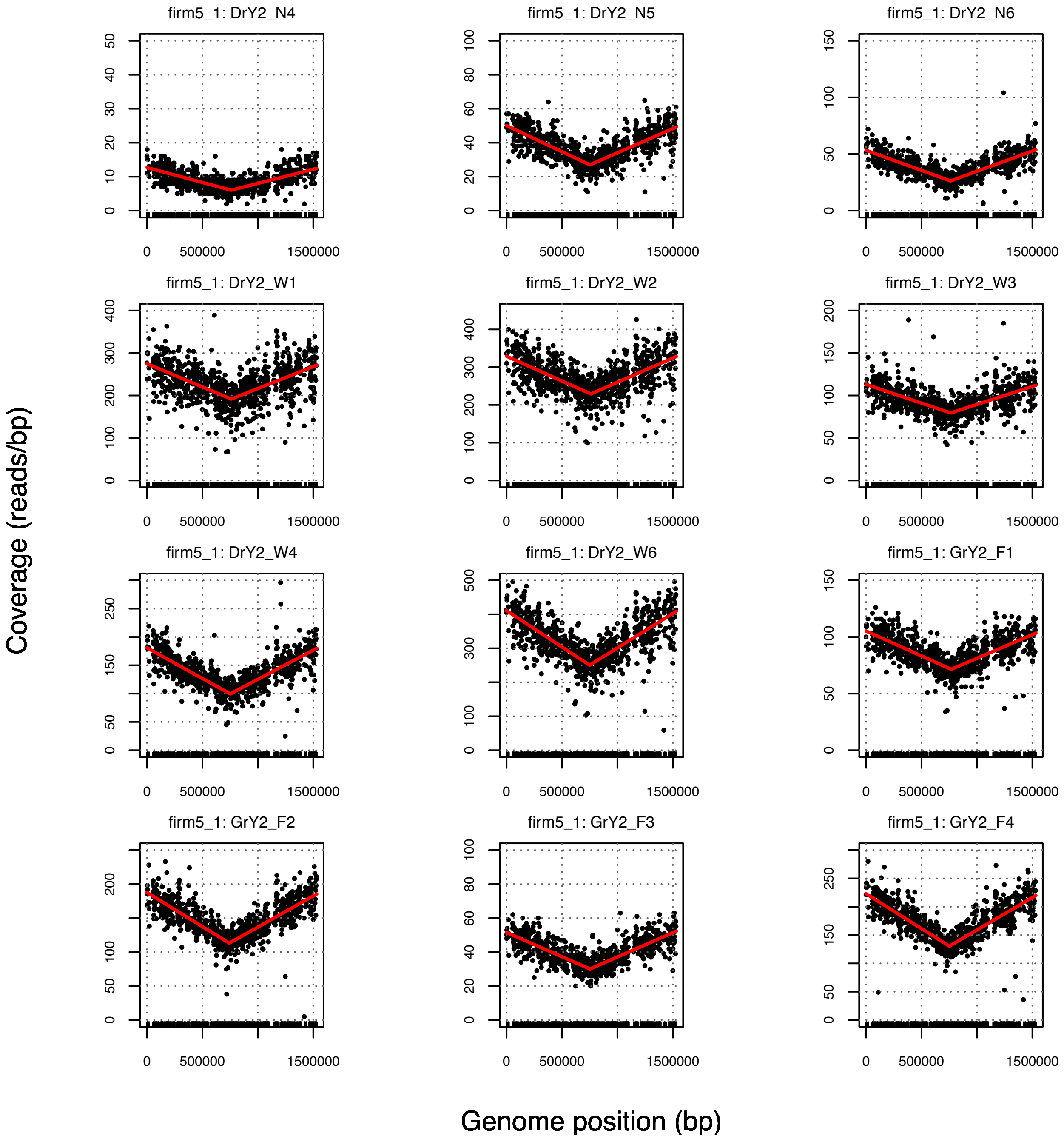
firm5_1: DrY2_N4
firm5_1: DrY2_N5
firm5_1: DrY2_N6
firm5_1: DrY2_W1
firm5_1: DrY2_W2
firm5_1: DrY2_W3
firm5_1: DrY2_W4
firm5_1: DrY2_W6
firm5_1: GrY2_F1
firm5_1: GrY2_F2
firm5_1: GrY2_F3
firm5_1: GrY2_F4
0
500000
1500000
Coverage (reads/bp)
Genome position (bp)

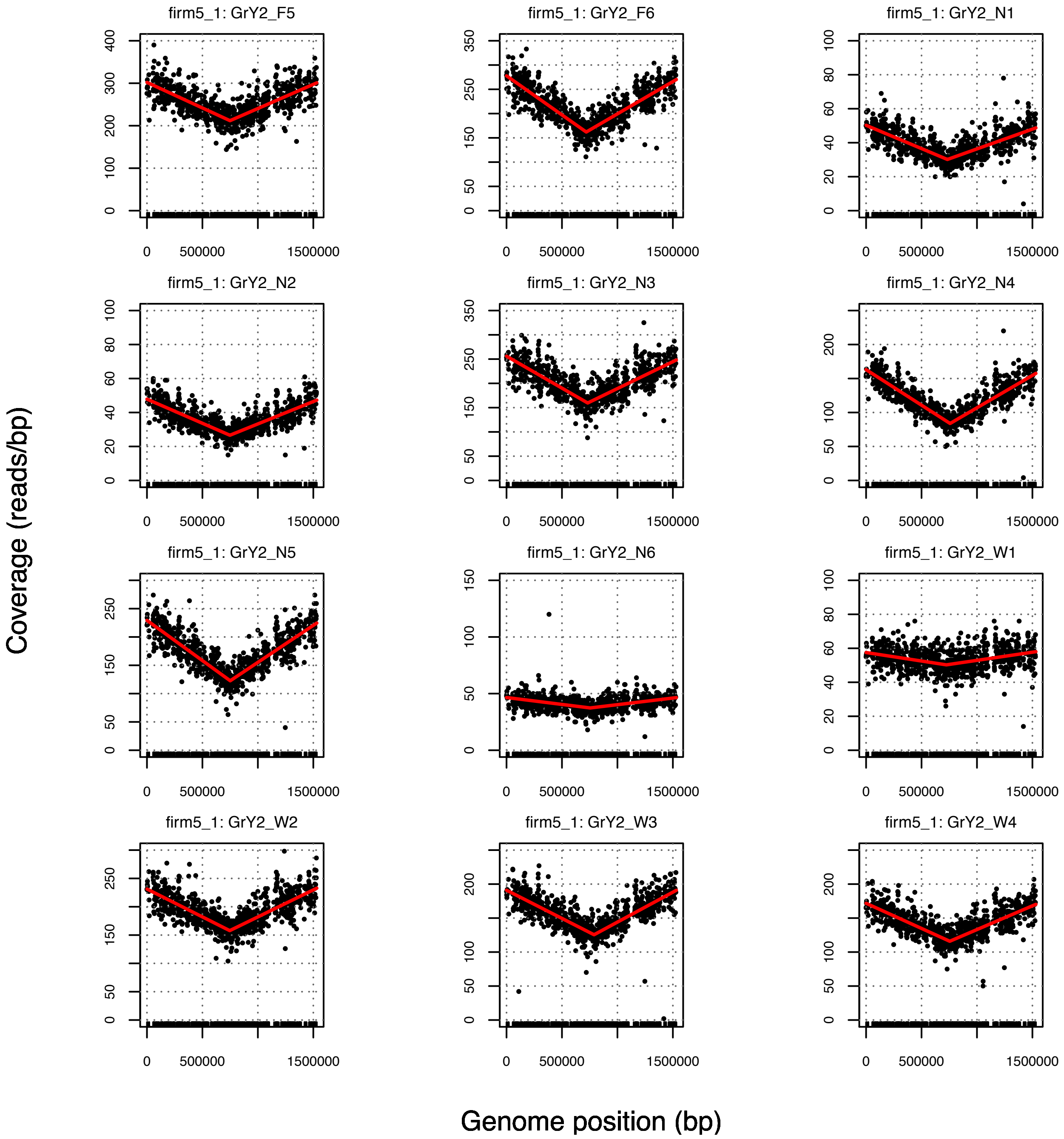
firm5_1: GrY2_F5
firm5_1: GrY2_F6
firm5_1: GrY2_N1
firm5_1: GrY2_N2
firm5_1: GrY2_N3
firm5_1: GrY2_N4
firm5_1: GrY2_N5
firm5_1: GrY2_N6
firm5_1: GrY2_W1
firm5_1: GrY2_W2
firm5_1: GrY2_W3
firm5_1: GrY2_W4
0
500000
1500000
Coverage (reads/bp)
Genome position (bp)

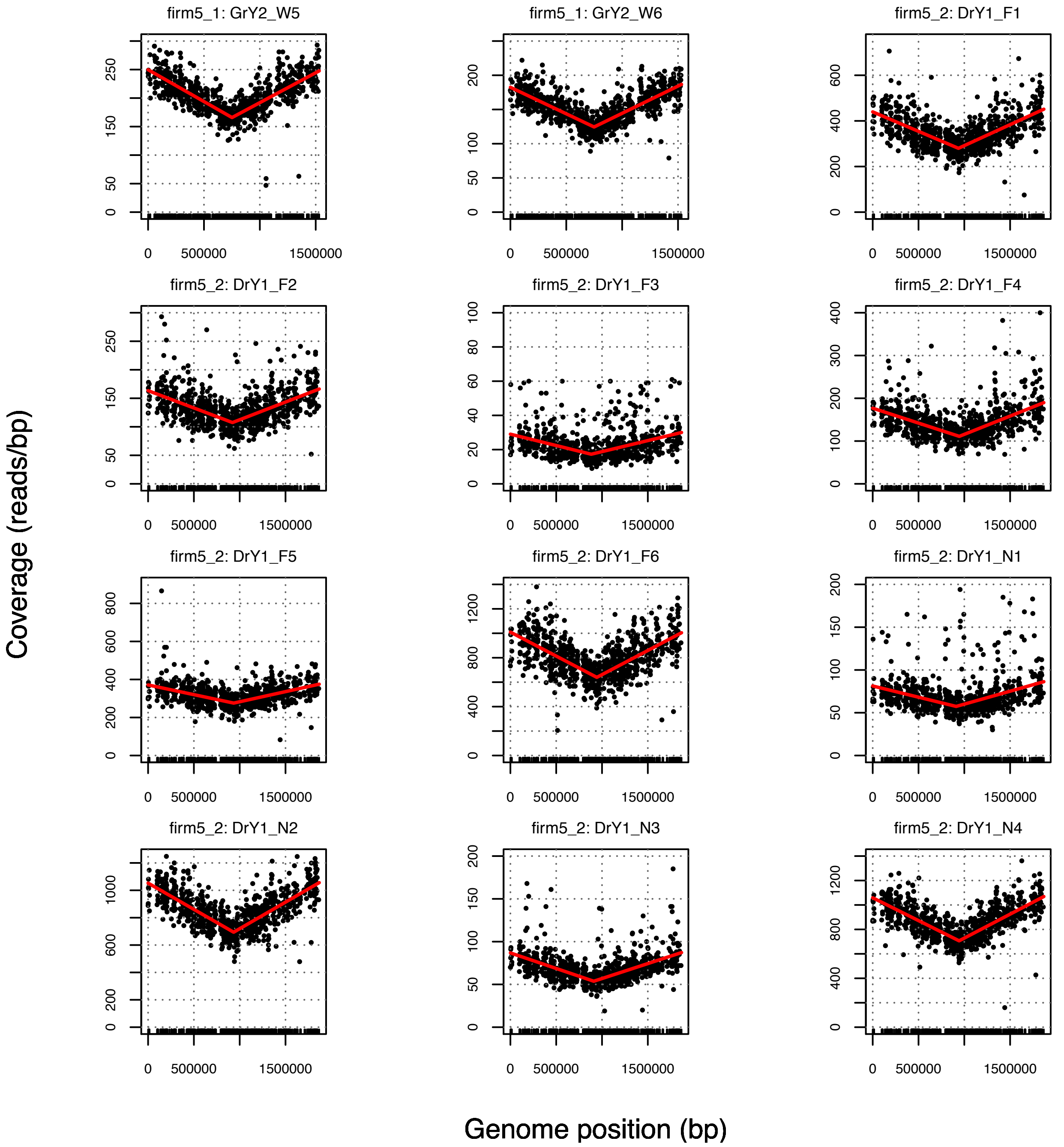
firm5_1: GrY2_W5
firm5_1: GrY2_W6
firm5_2: DrY1_F1
firm5_2: DrY1_F2
firm5_2: DrY1_F3
firm5_2: DrY1_F4
firm5_2: DrY1_F5
firm5_2: DrY1_F6
firm5_2: DrY1_N1
firm5_2: DrY1_N2
firm5_2: DrY1_N3
firm5_2: DrY1_N4
Coverage (reads/bp)
Genome position (bp)

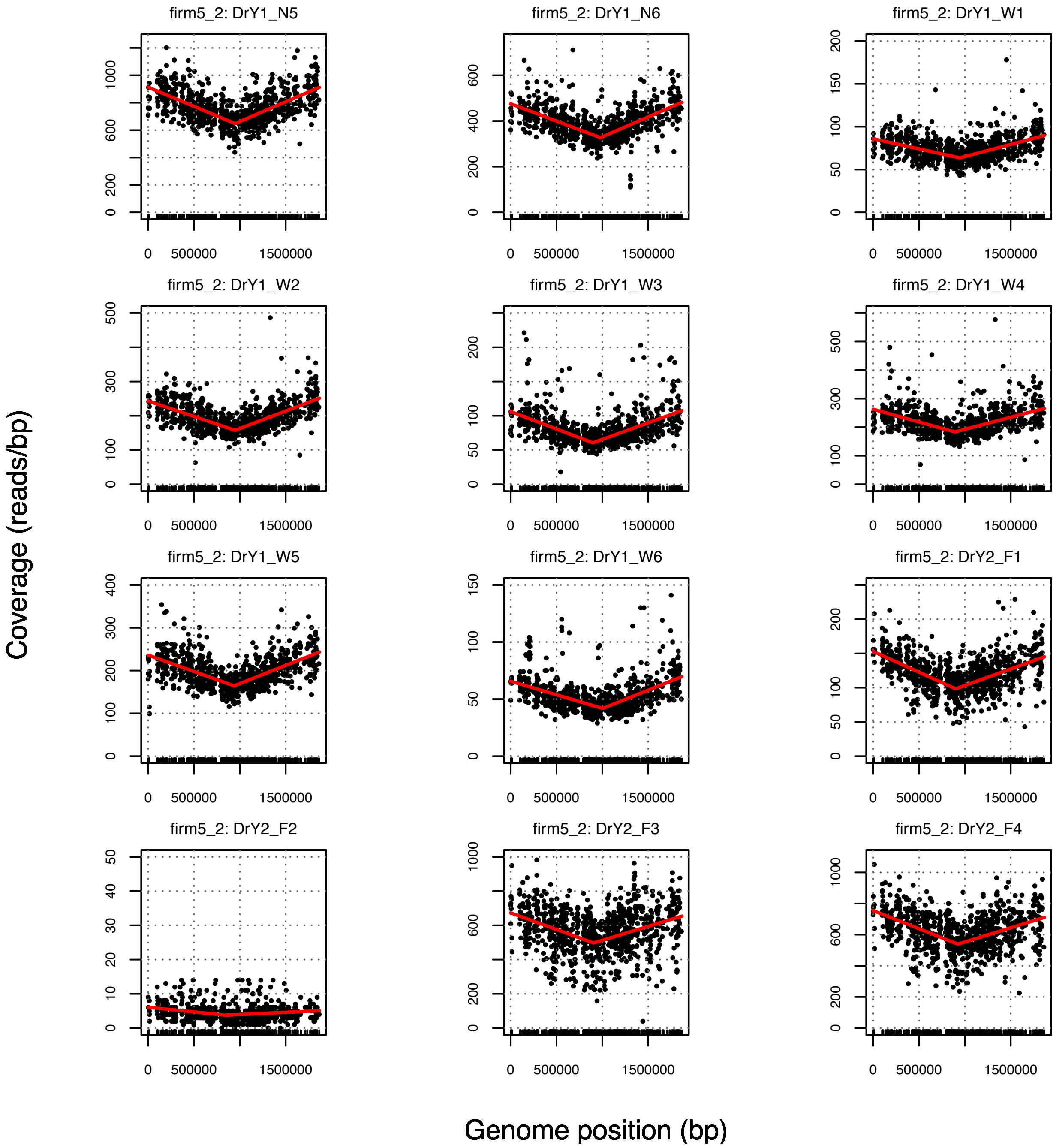
firm5_2: DrY1_N5
firm5_2: DrY1_N6
firm5_2: DrY1_W1
firm5_2: DrY1_W2
firm5_2: DrY1_W3
firm5_2: DrY1_W4
firm5_2: DrY1_W5
firm5_2: DrY1_W6
firm5_2: DrY2_F1
firm5_2: DrY2_F2
firm5_2: DrY2_F3
firm5_2: DrY2_F4
Coverage (reads/bp)
Genome position (bp)

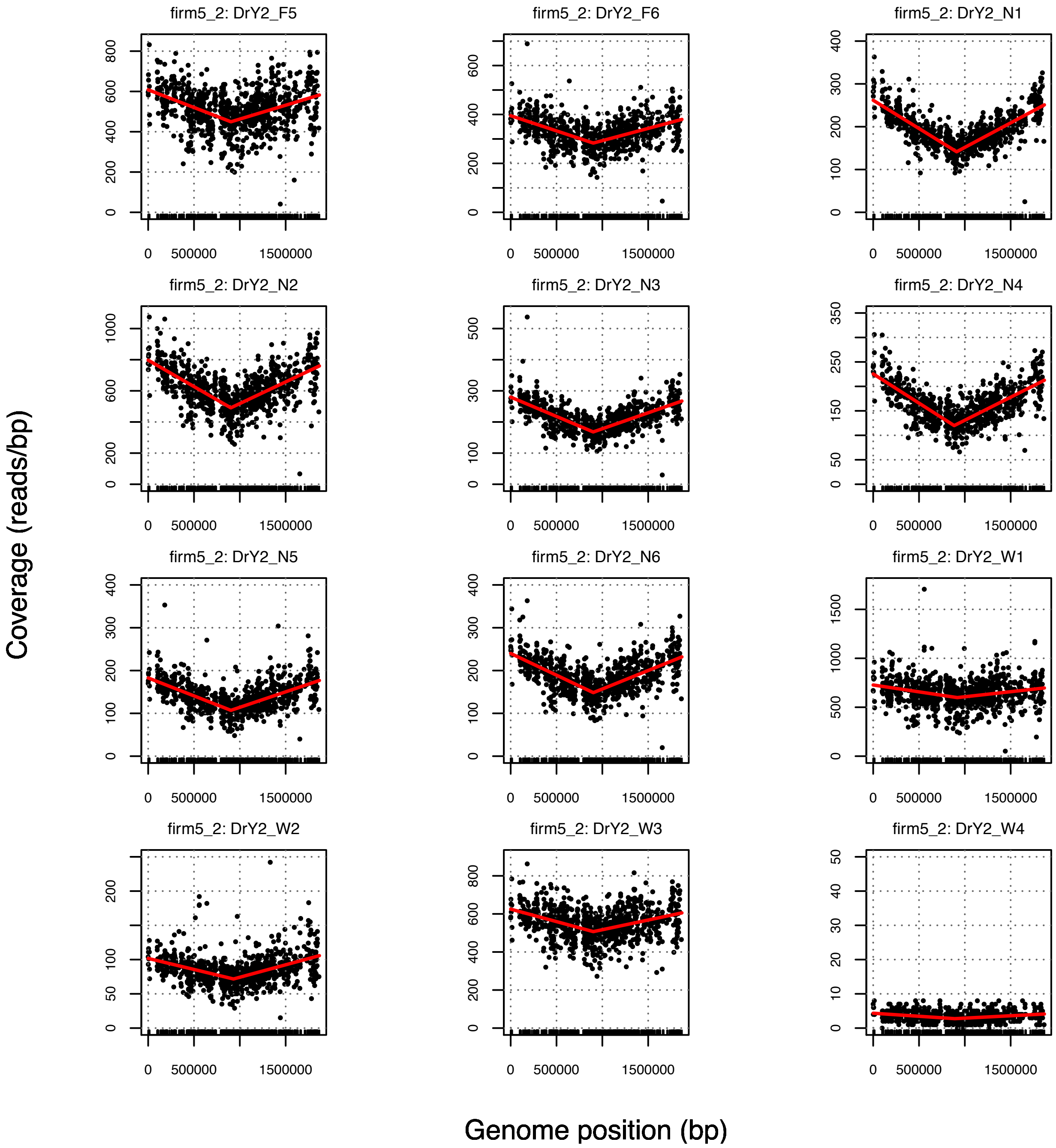
firm5_2: DrY2_F5
firm5_2: DrY2_F6
firm5_2: DrY2_N1
firm5_2: DrY2_N2
firm5_2: DrY2_N3
firm5_2: DrY2_N4
firm5_2: DrY2_N5
firm5_2: DrY2_N6
firm5_2: DrY2_W1
firm5_2: DrY2_W2
firm5_2: DrY2_W3
firm5_2: DrY2_W4
0 500000 1500000
Coverage (reads/bp)
Genome position (bp)

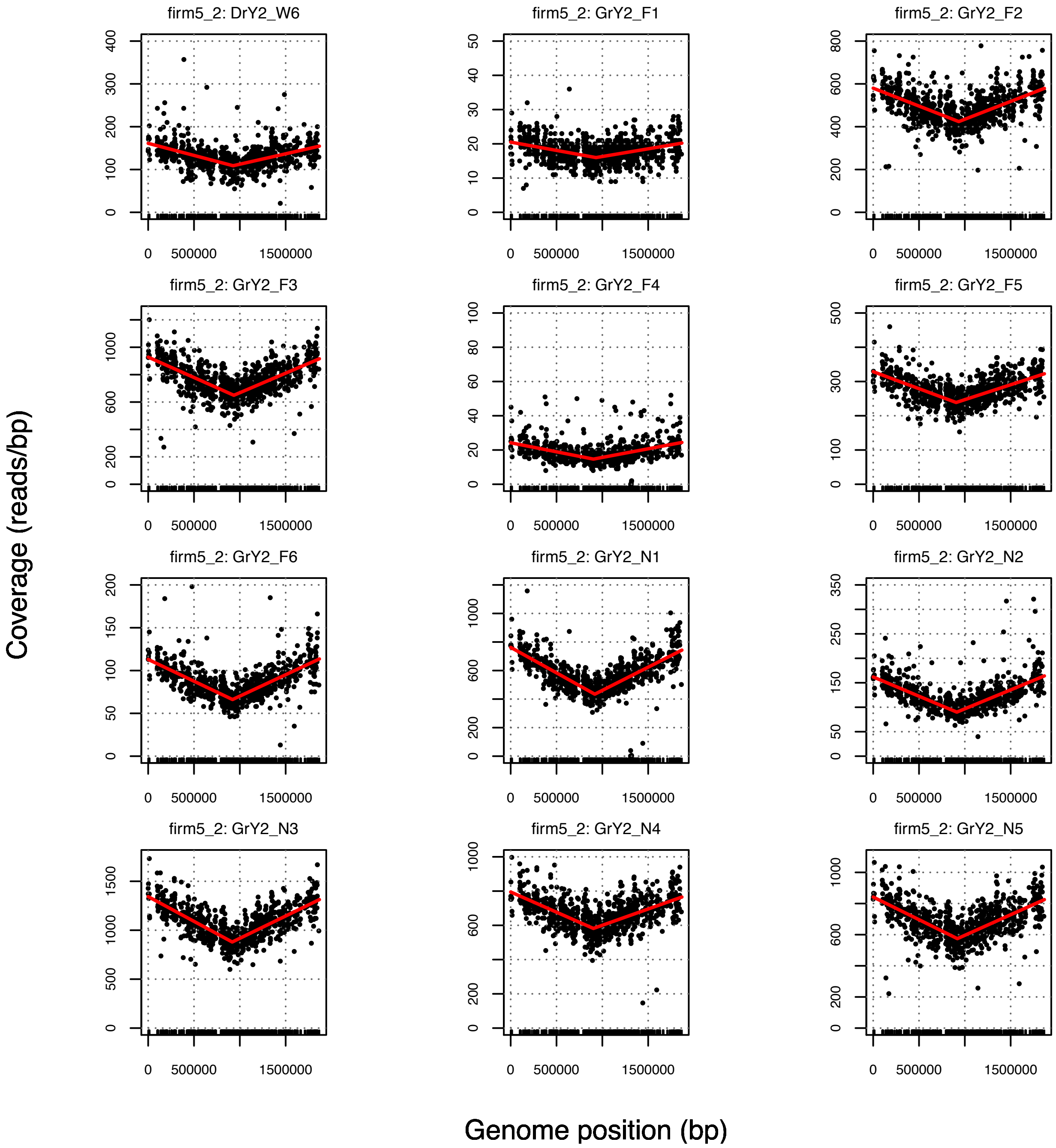
firm5_2: DrY2_W6
firm5_2: GrY2_F1
firm5_2: GrY2_F2
firm5_2: GrY2_F3
firm5_2: GrY2_F4
firm5_2: GrY2_F5
firm5_2: GrY2_F6
firm5_2: GrY2_N1
firm5_2: GrY2_N2
firm5_2: GrY2_N3
firm5_2: GrY2_N4
firm5_2: GrY2_N5
Coverage (reads/bp)
Genome position (bp)

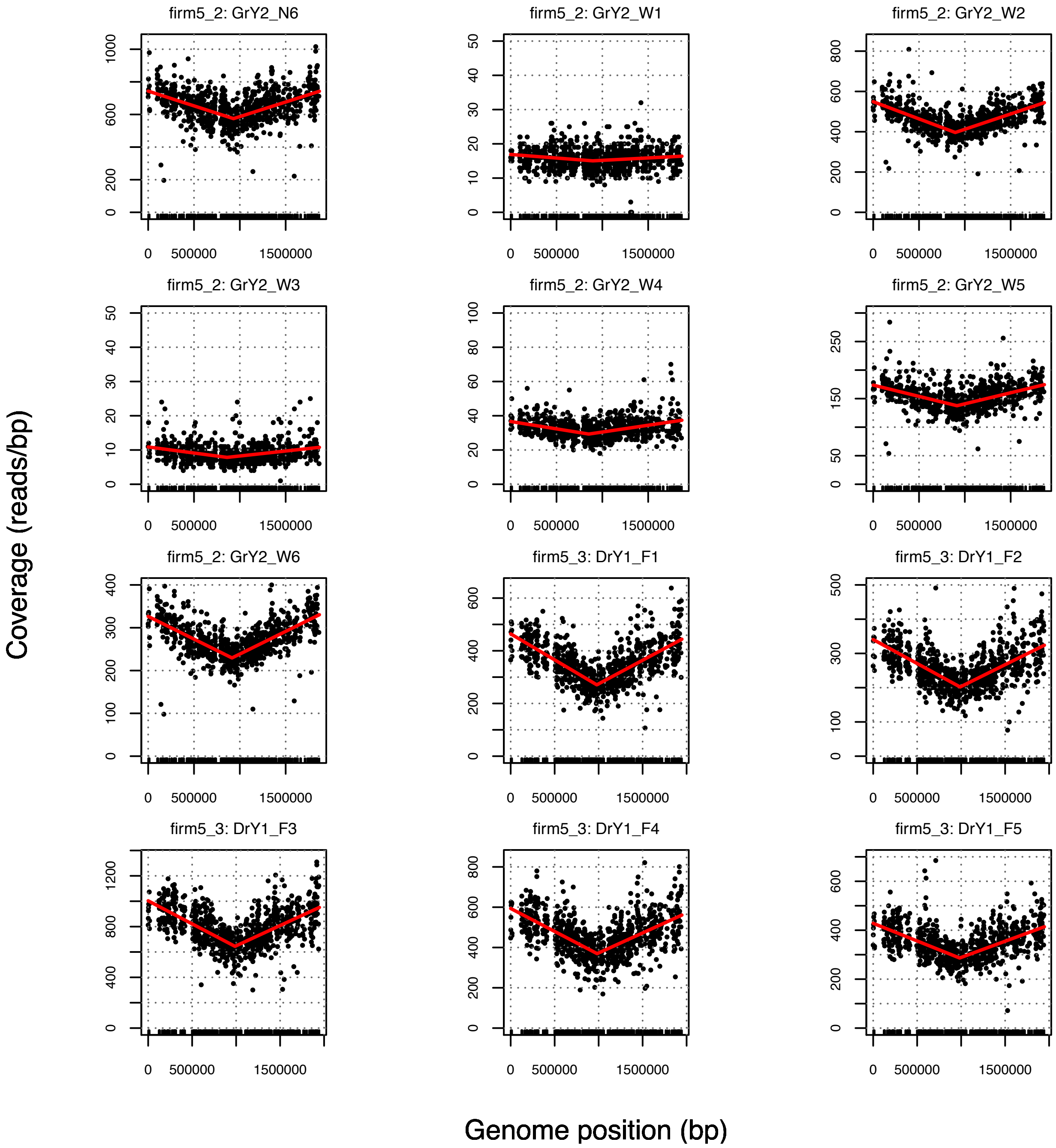
firm5_2: GrY2_N6
firm5_2: GrY2_W1
firm5_2: GrY2_W2
firm5_2: GrY2_W3
firm5_2: GrY2_W4
firm5_2: GrY2_W5
firm5_2: GrY2_W6
firm5_3: DrY1_F1
firm5_3: DrY1_F2
firm5_3: DrY1_F3
firm5_3: DrY1_F4
firm5_3: DrY1_F5
Coverage (reads/bp)
Genome position (bp)
0
500000
1500000

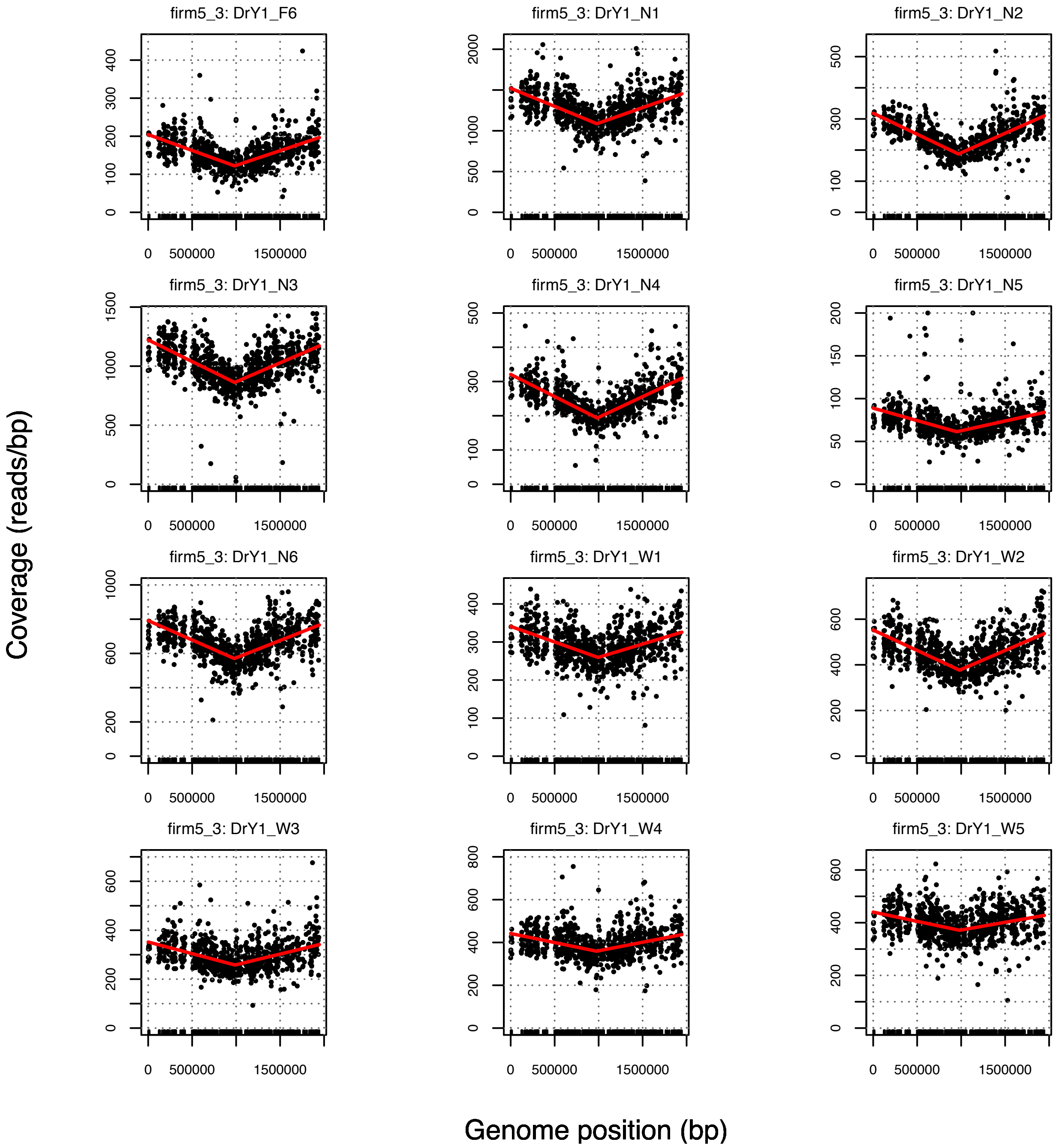
firm5_3: DrY1_F6
firm5_3: DrY1_N1
firm5_3: DrY1_N2
firm5_3: DrY1_N3
firm5_3: DrY1_N4
firm5_3: DrY1_N5
firm5_3: DrY1_N6
firm5_3: DrY1_W1
firm5_3: DrY1_W2
firm5_3: DrY1_W3
firm5_3: DrY1_W4
firm5_3: DrY1_W5
Coverage (reads/bp)
Genome position (bp)

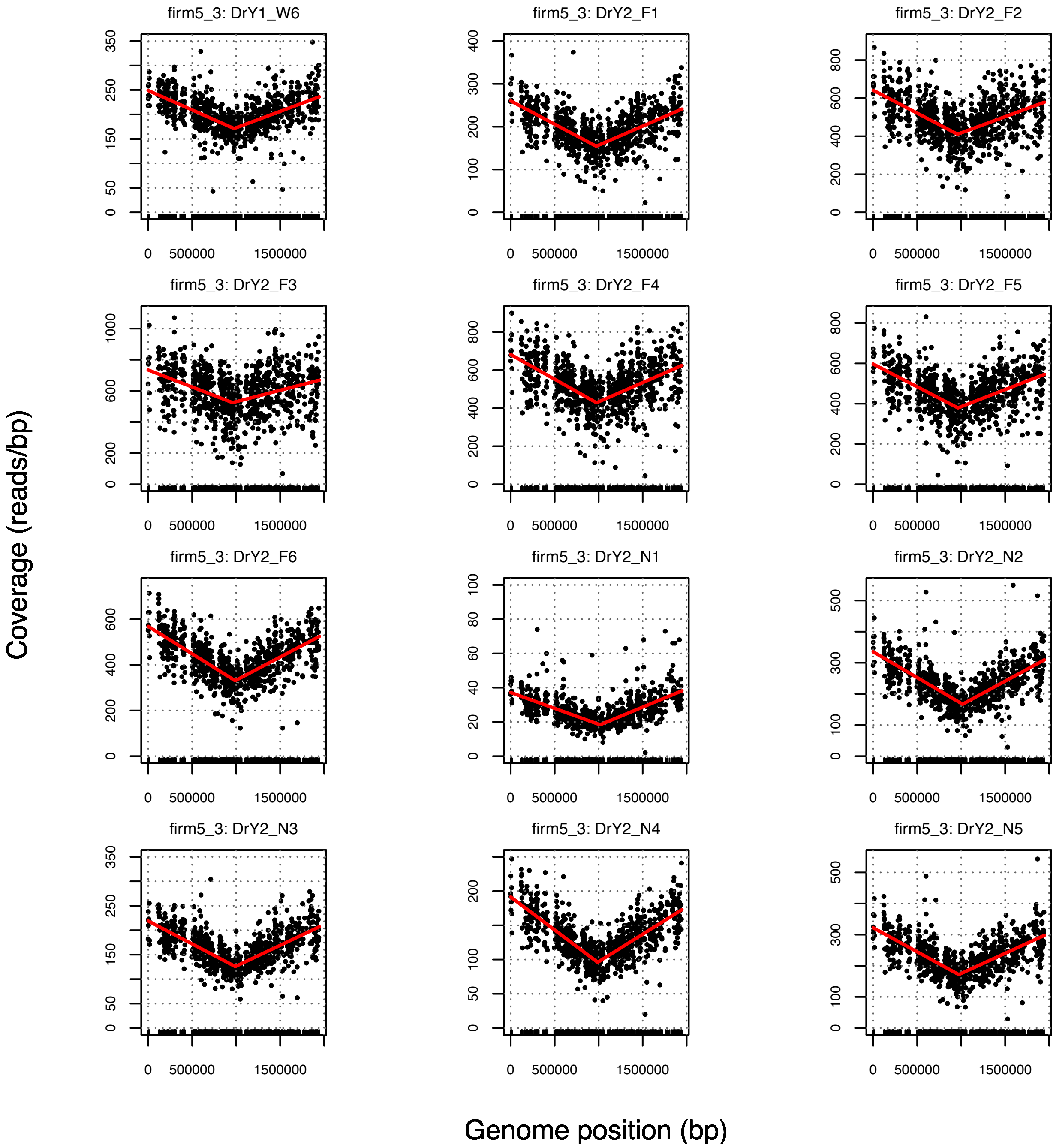

firm5_3: DrY1_W6
firm5_3: DrY2_F1
firm5_3: DrY2_F2
firm5_3: DrY2_F3
firm5_3: DrY2_F4
firm5_3: DrY2_F5
firm5_3: DrY2_F6
firm5_3: DrY2_N1
firm5_3: DrY2_N2
firm5_3: DrY2_N3
firm5_3: DrY2_N4
firm5_3: DrY2_N5
0
500000
1500000
Coverage (reads/bp)
Genome position (bp)

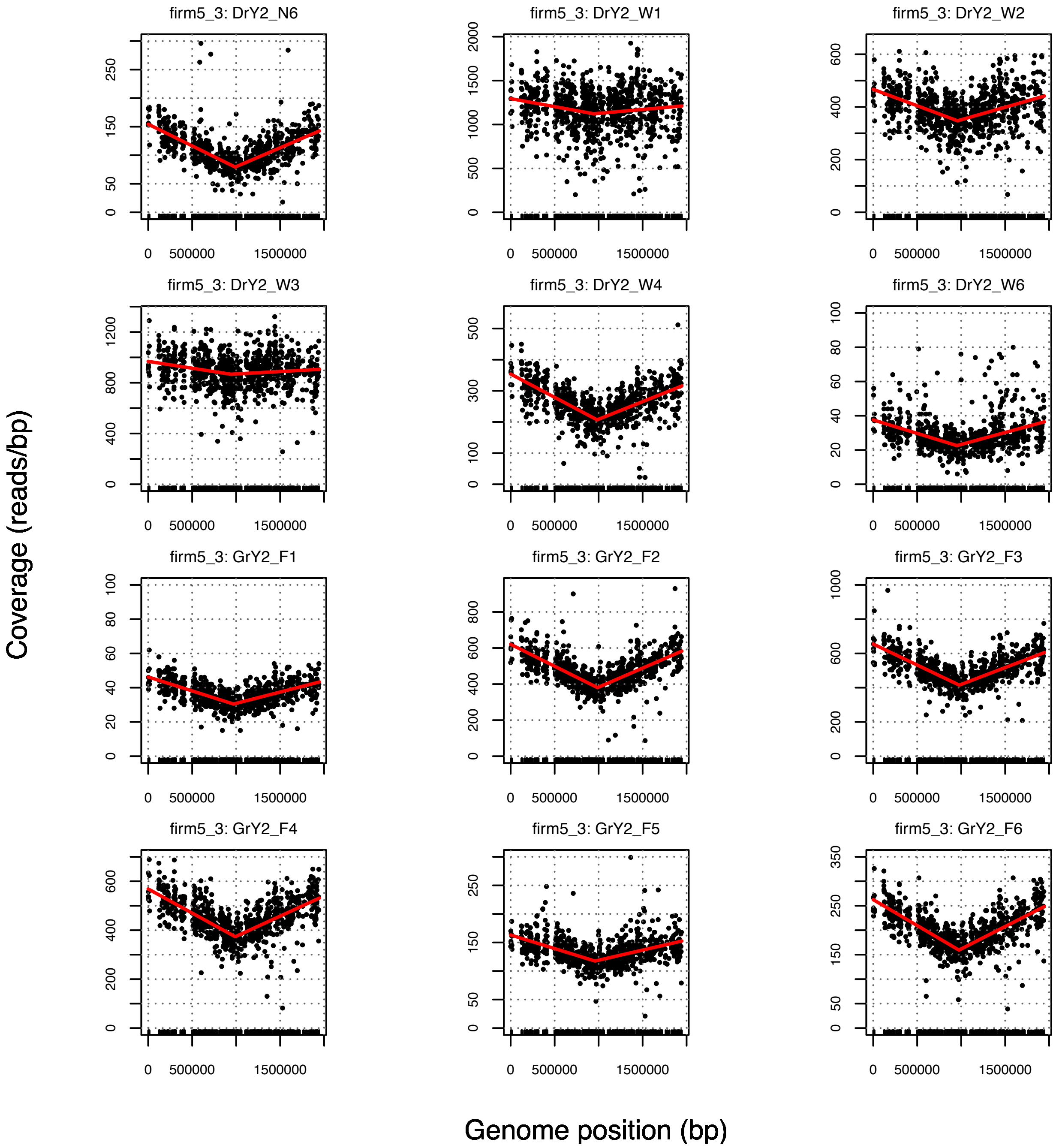

firm5_3: DrY2_N6
firm5_3: DrY2_W1
firm5_3: DrY2_W2
firm5_3: DrY2_W3
firm5_3: DrY2_W4
firm5_3: DrY2_W6
firm5_3: GrY2_F1
firm5_3: GrY2_F2
firm5_3: GrY2_F3
firm5_3: GrY2_F4
firm5_3: GrY2_F5
firm5_3: GrY2_F6
Coverage (reads/bp)
Genome position (bp)

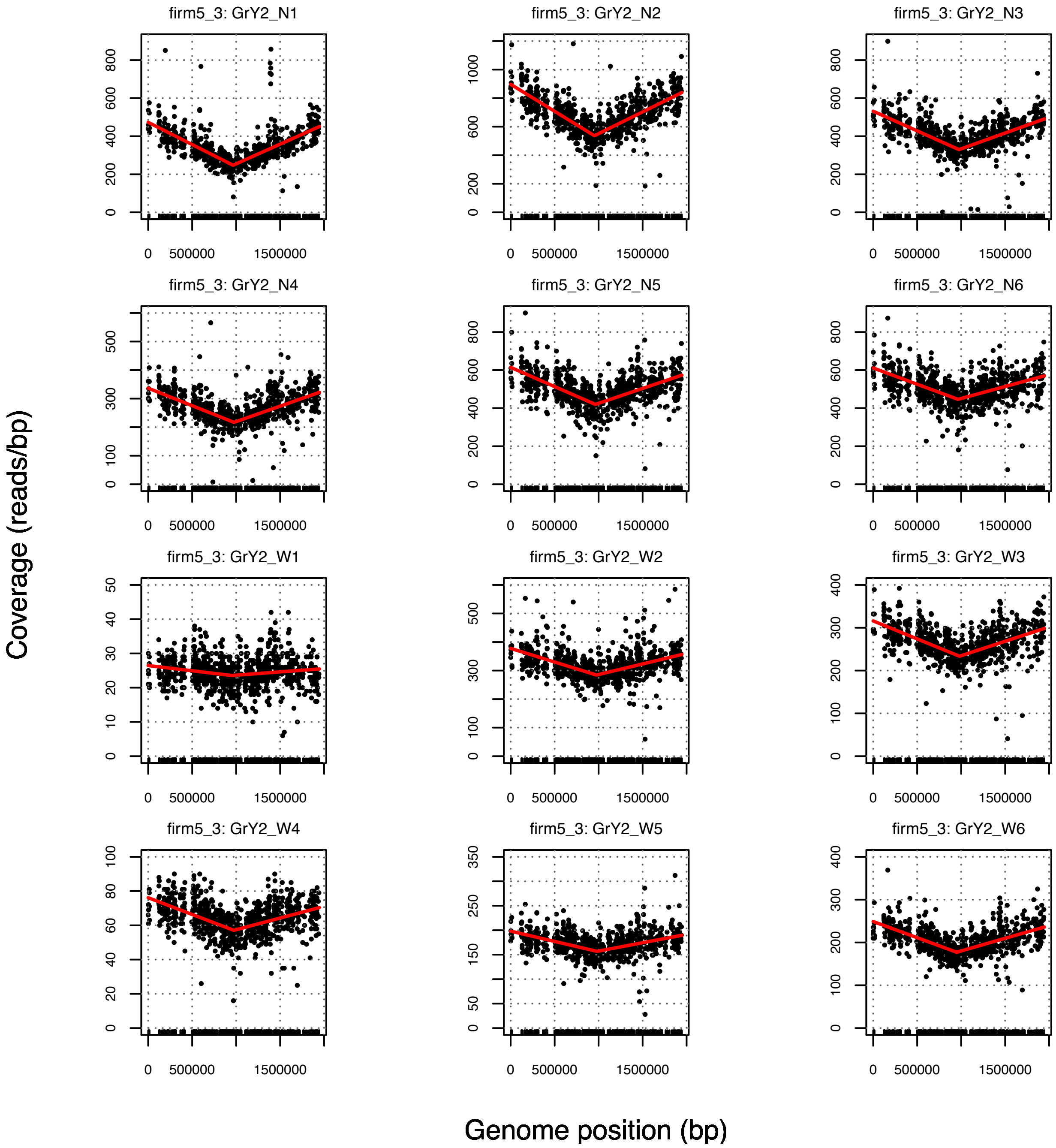
firm5_3: GrY2_N1
firm5_3: GrY2_N2
firm5_3: GrY2_N3
firm5_3: GrY2_N4
firm5_3: GrY2_N5
firm5_3: GrY2_N6
firm5_3: GrY2_W1
firm5_3: GrY2_W2
firm5_3: GrY2_W3
firm5_3: GrY2_W4
firm5_3: GrY2_W5
firm5_3: GrY2_W6
Coverage (reads/bp)
Genome position (bp)

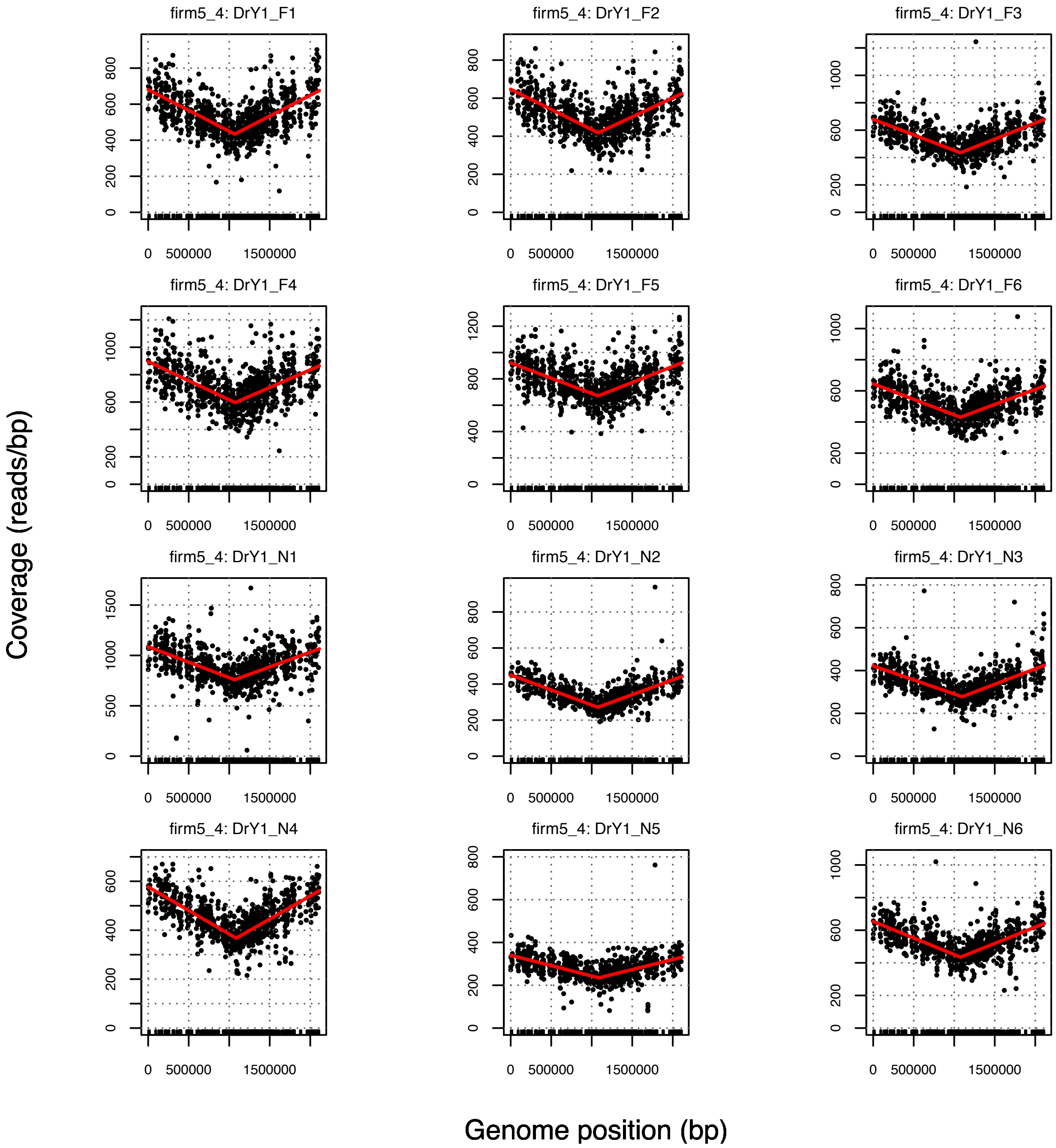
firm5_4: DrY1_F1
firm5_4: DrY1_F2
firm5_4: DrY1_F3
firm5_4: DrY1_F4
firm5_4: DrY1_F5
firm5_4: DrY1_F6
firm5_4: DrY1_N1
firm5_4: DrY1_N2
firm5_4: DrY1_N3
firm5_4: DrY1_N4
firm5_4: DrY1_N5
firm5_4: DrY1_N6
0 500000 1500000
Coverage (reads/bp)
Genome position (bp)

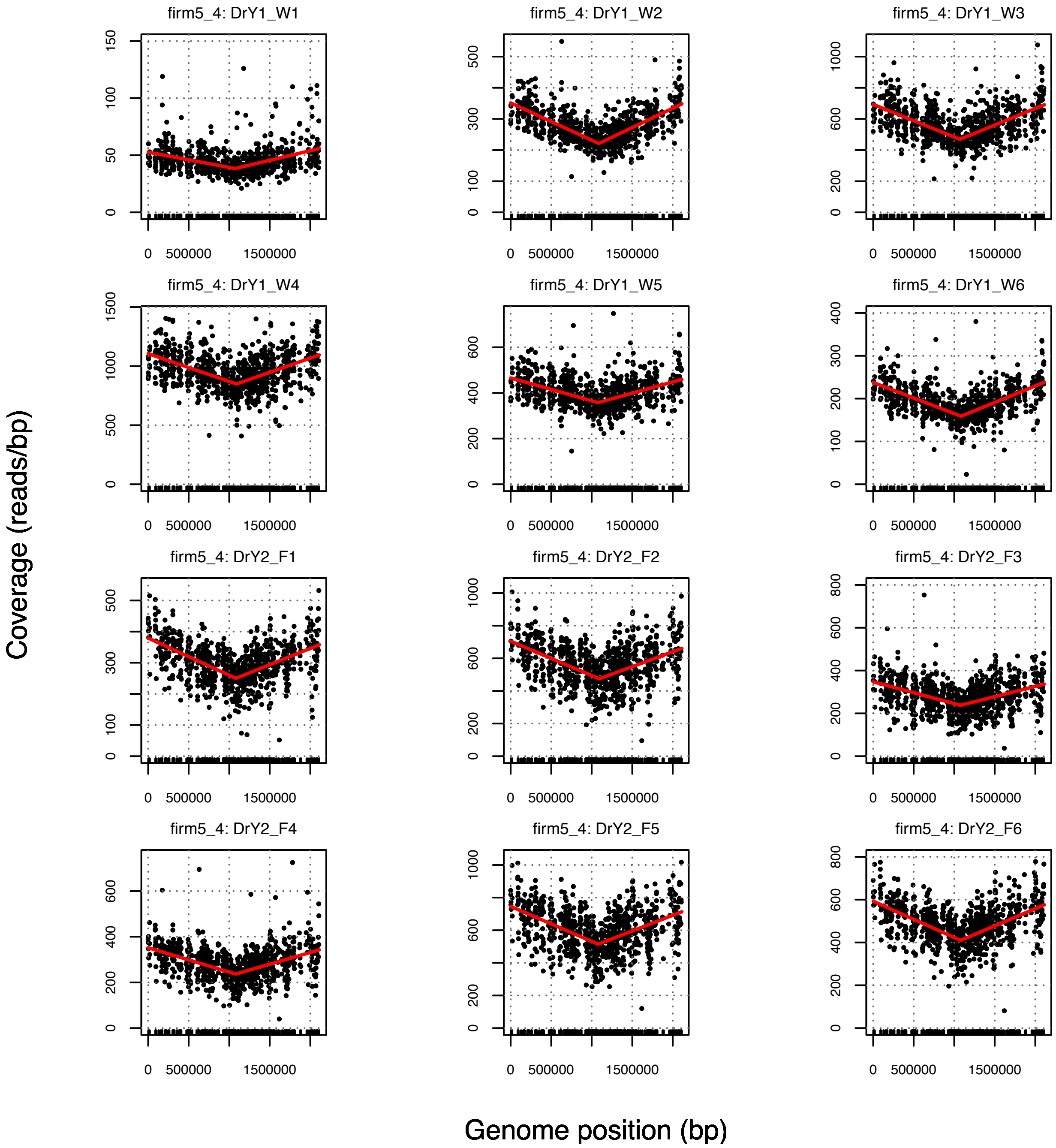

firm5_4: DrY1_W1
firm5_4: DrY1_W2
firm5_4: DrY1_W3
firm5_4: DrY1_W4
firm5_4: DrY1_W5
firm5_4: DrY1_W6
firm5_4: DrY2_F1
firm5_4: DrY2_F2
firm5_4: DrY2_F3
firm5_4: DrY2_F4
firm5_4: DrY2_F5
firm5_4: DrY2_F6
Coverage (reads/bp)
Genome position (bp)
0
500000
1500000

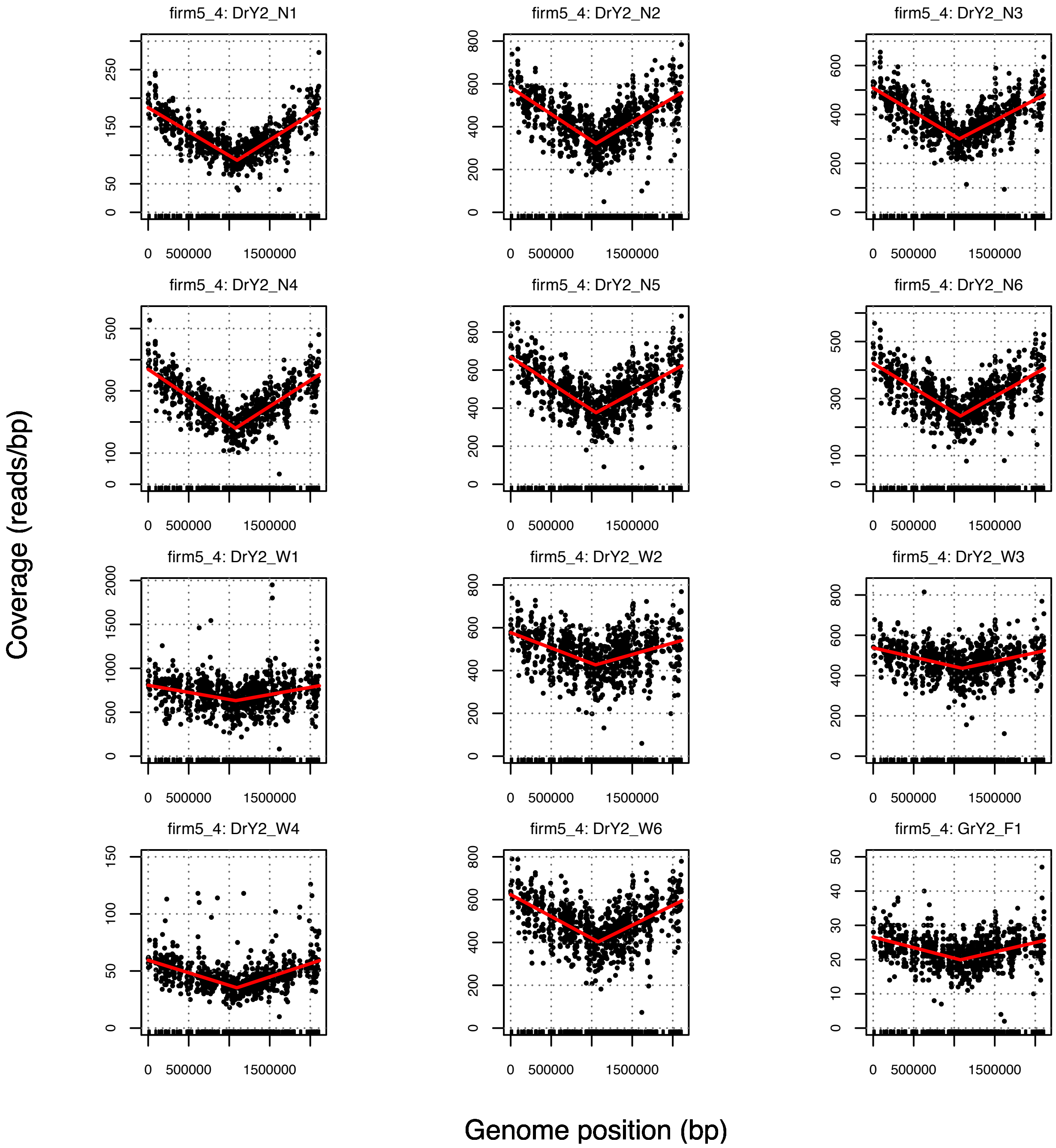
firm5_4: DrY2_N1
firm5_4: DrY2_N2
firm5_4: DrY2_N3
firm5_4: DrY2_N4
firm5_4: DrY2_N5
firm5_4: DrY2_N6
firm5_4: DrY2_W1
firm5_4: DrY2_W2
firm5_4: DrY2_W3
firm5_4: DrY2_W4
firm5_4: DrY2_W6
firm5_4: GrY2_F1
Coverage (reads/bp)
Genome position (bp)
0 500000 1500000

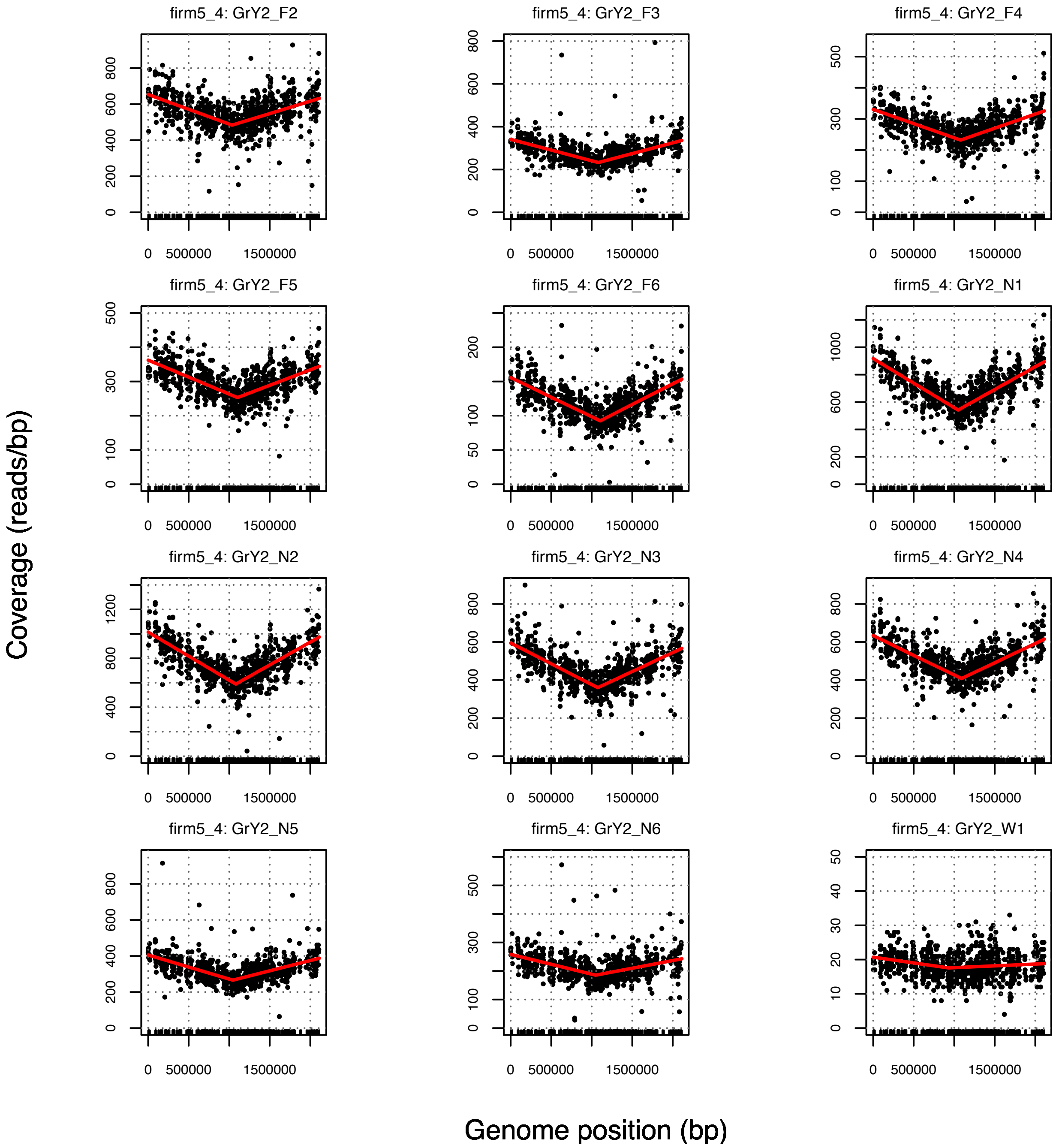
firm5_4: GrY2_F2
firm5_4: GrY2_F3
firm5_4: GrY2_F4
firm5_4: GrY2_F5
firm5_4: GrY2_F6
firm5_4: GrY2_N1
firm5_4: GrY2_N2
firm5_4: GrY2_N3
firm5_4: GrY2_N4
firm5_4: GrY2_N5
firm5_4: GrY2_N6
firm5_4: GrY2_W1
0 500000 1500000
Coverage (reads/bp)
Genome position (bp)

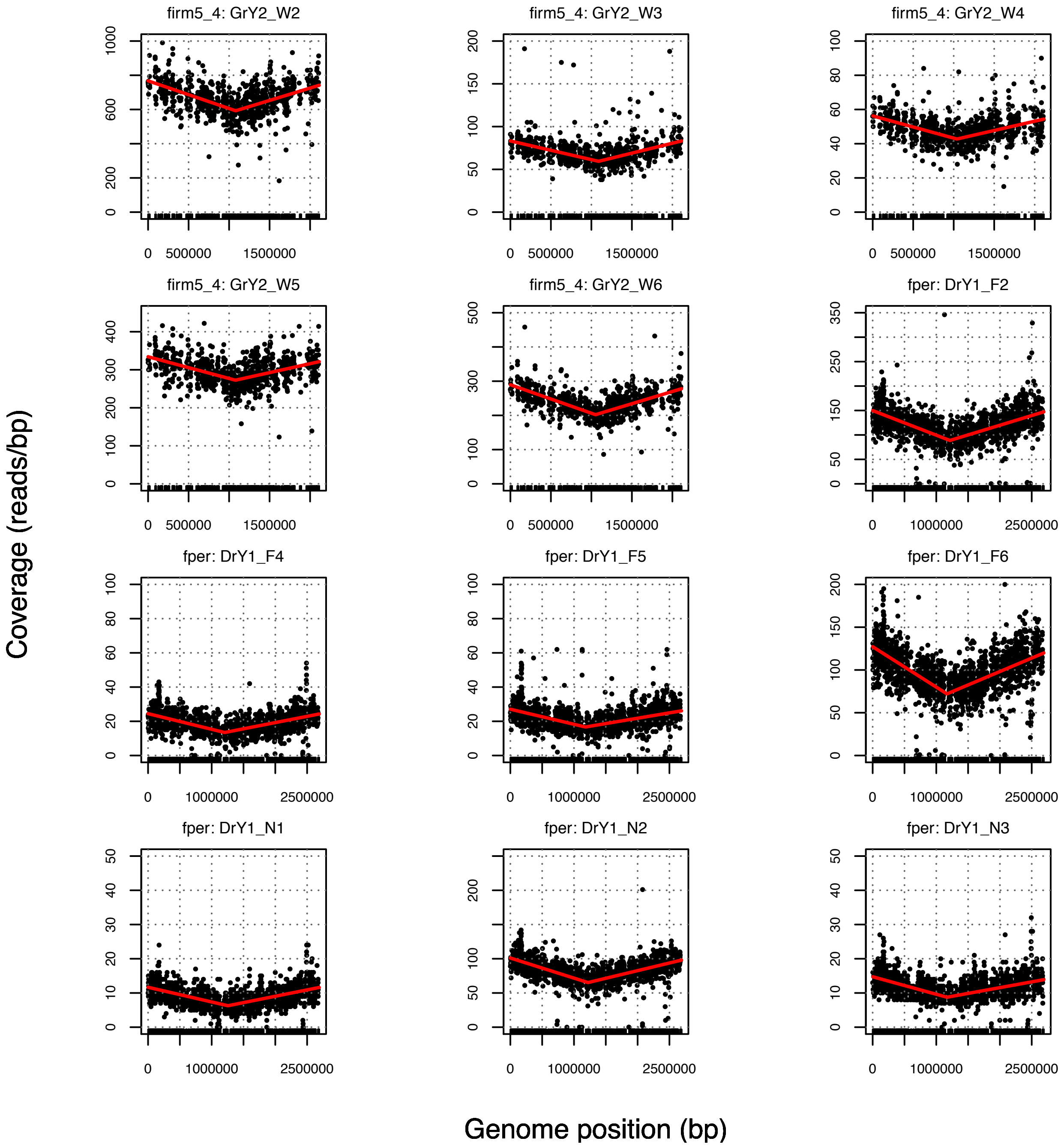
firm5_4: GrY2_W2
firm5_4: GrY2_W3
firm5_4: GrY2_W4
firm5_4: GrY2_W5
firm5_4: GrY2_W6
fper: DrY1_F2
fper: DrY1_F4
fper: DrY1_F5
fper: DrY1_F6
fper: DrY1_N1
fper: DrY1_N2
fper: DrY1_N3
Coverage (reads/bp)
Genome position (bp)

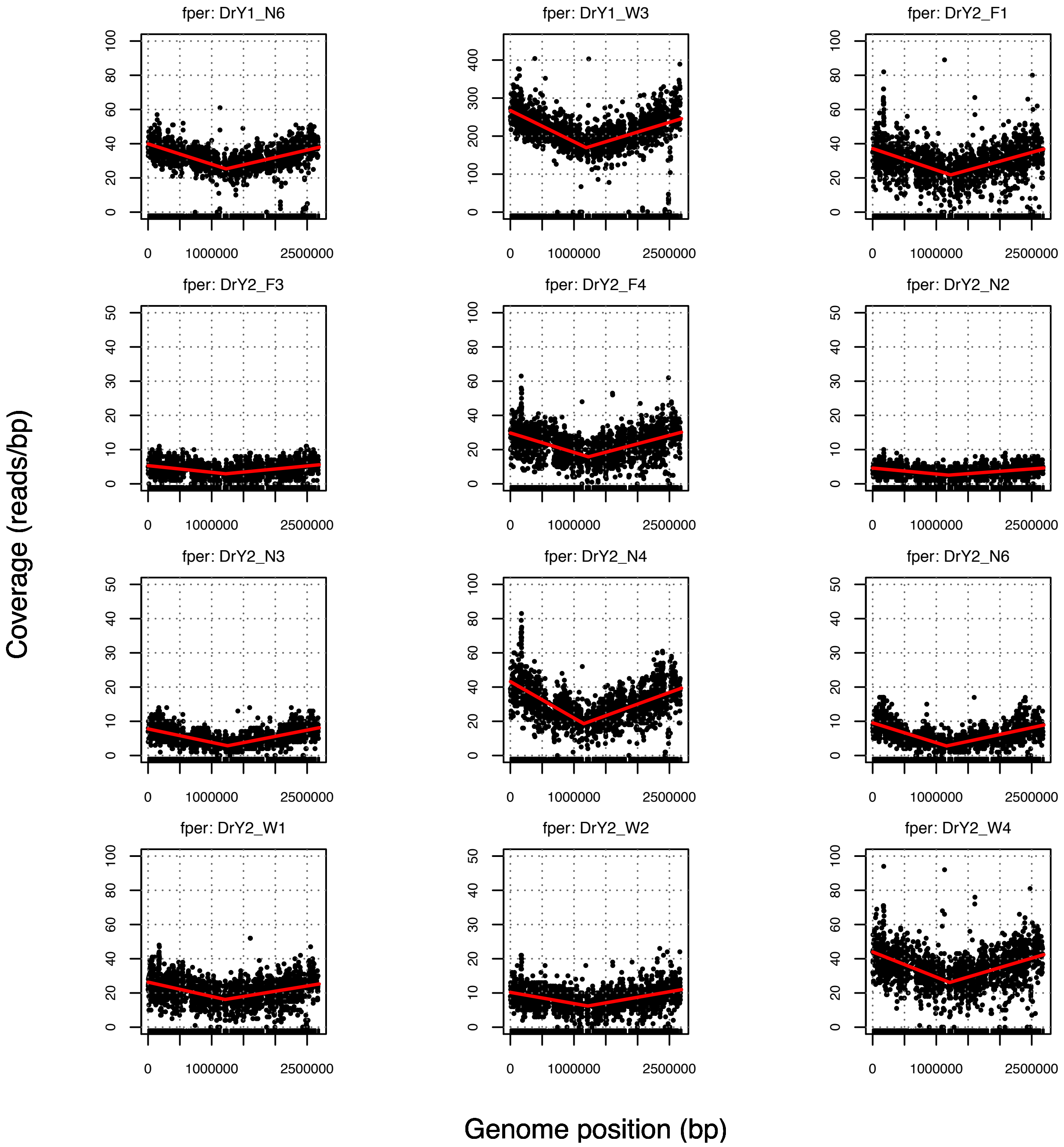
fper: DrY1_N6
fper: DrY1_W3
fper: DrY2_F1
fper: DrY2_F3
fper: DrY2_F4
fper: DrY2_N2
fper: DrY2_N3
fper: DrY2_N4
fper: DrY2_N6
fper: DrY2_W1
fper: DrY2_W2
fper: DrY2_W4
0
1000000
2500000
Coverage (reads/bp)
Genome position (bp)

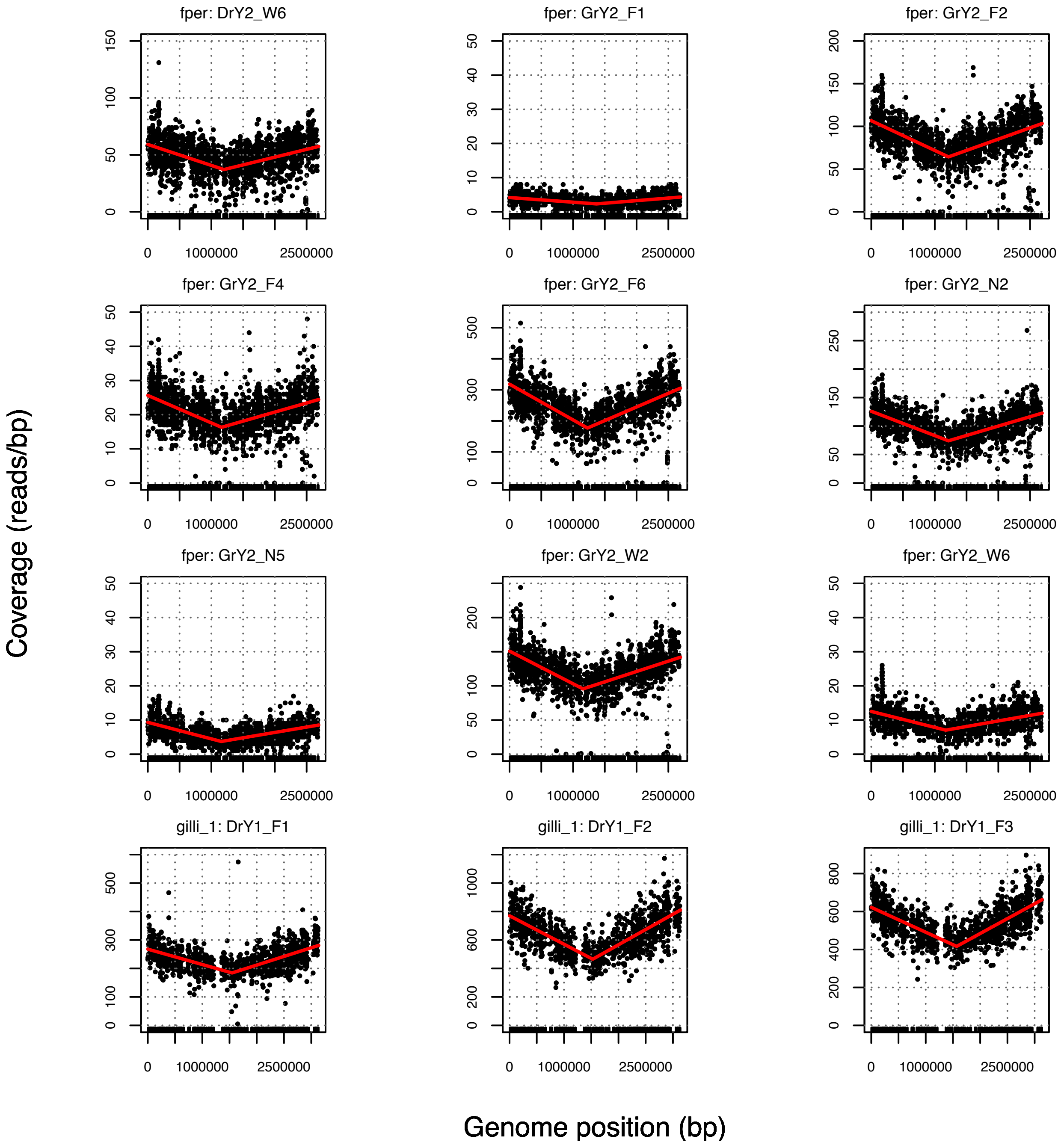

fper: DrY2_W6
fper: GrY2_F1
fper: GrY2_F2
fper: GrY2_F4
fper: GrY2_F6
fper: GrY2_N2
fper: GrY2_N5
fper: GrY2_W2
fper: GrY2_W6
gilli_1: DrY1_F1
gilli_1: DrY1_F2
gilli_1: DrY1_F3
Coverage (reads/bp)
Genome position (bp)

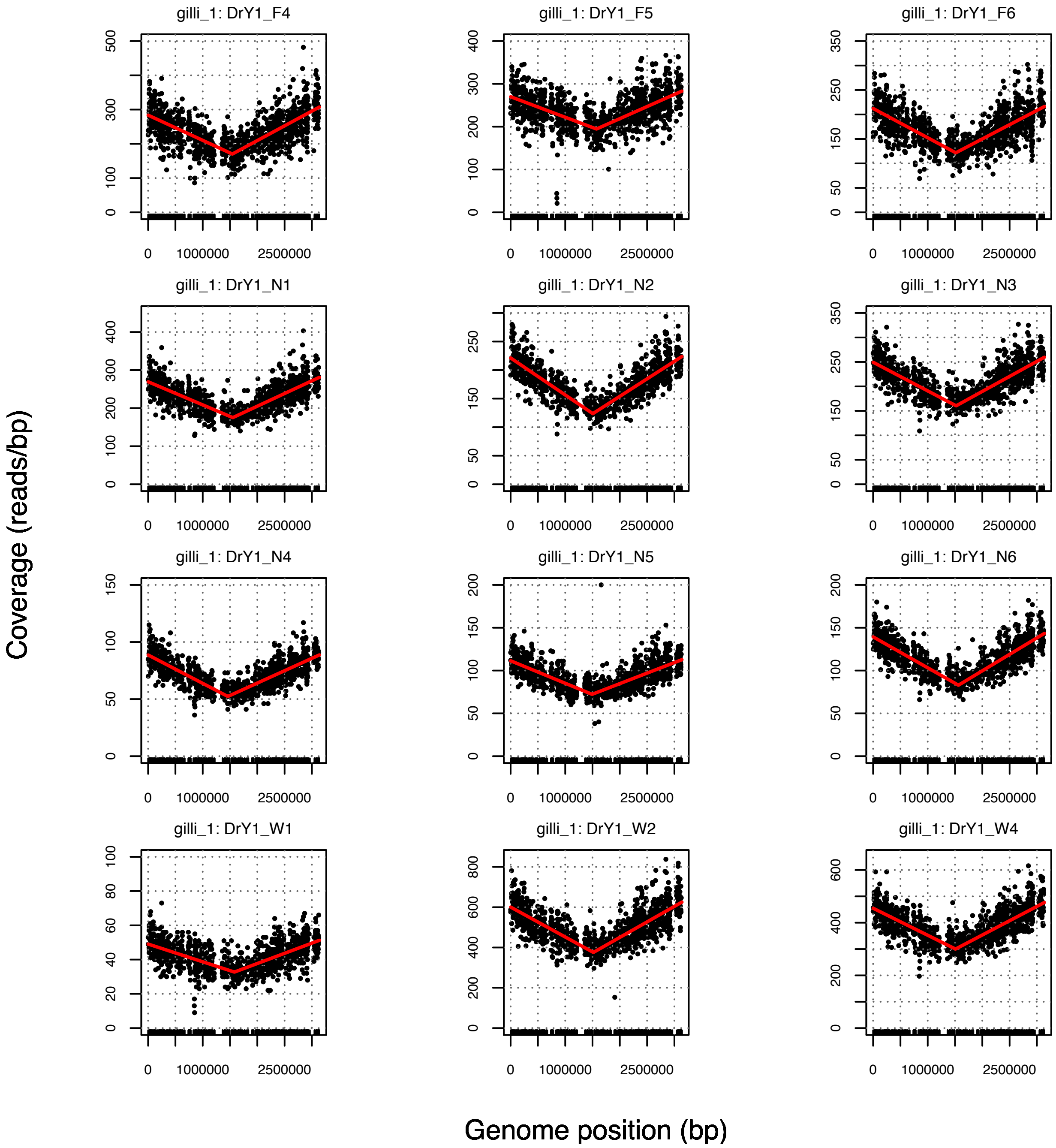
gilli_1: DrY1_F4
gilli_1: DrY1_F5
gilli_1: DrY1_F6
gilli_1: DrY1_N1
gilli_1: DrY1_N2
gilli_1: DrY1_N3
gilli_1: DrY1_N4
gilli_1: DrY1_N5
gilli_1: DrY1_N6
gilli_1: DrY1_W1
gilli_1: DrY1_W2
gilli_1: DrY1_W4
Coverage (reads/bp)
Genome position (bp)

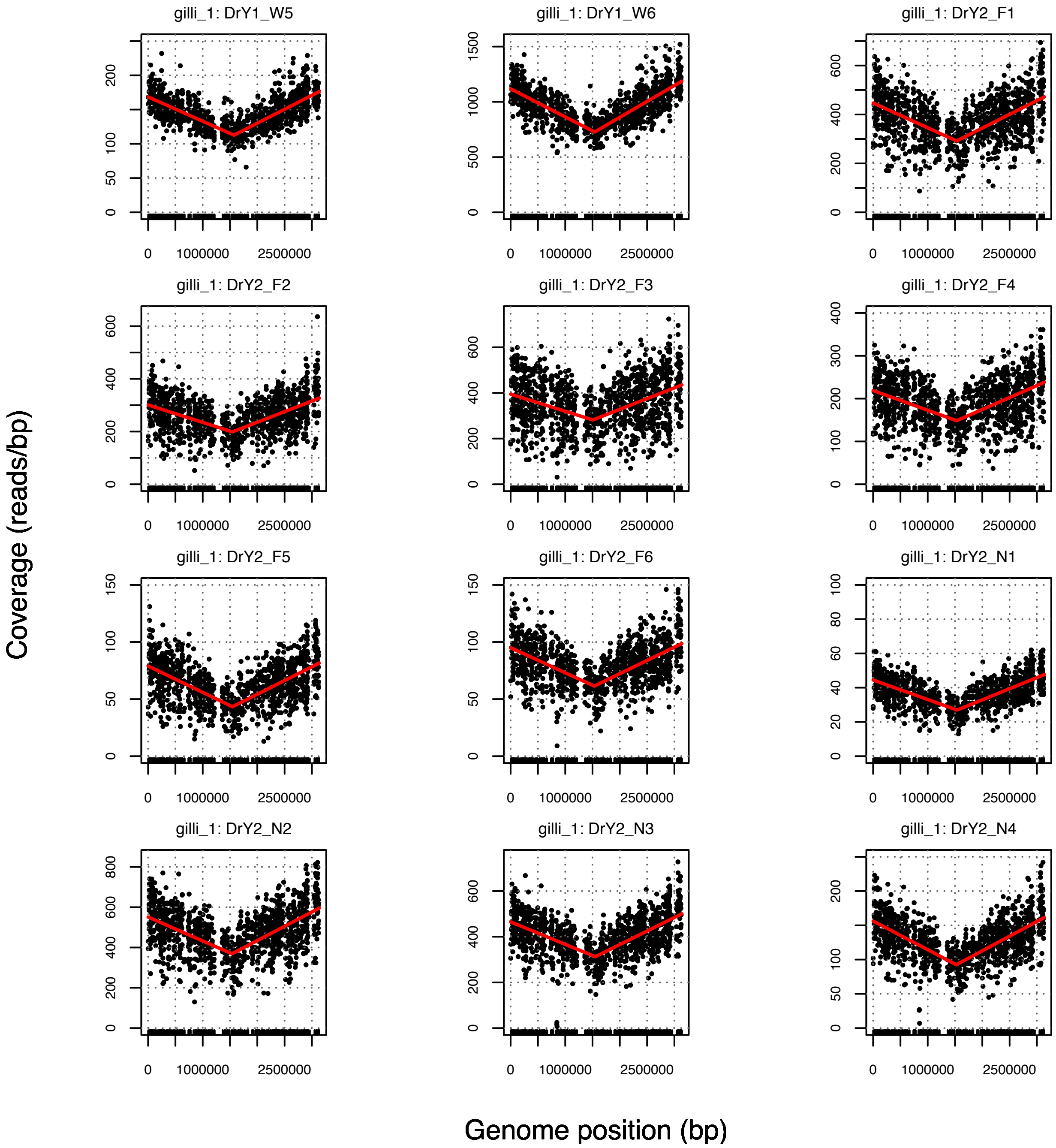

gilli_1: DrY1_W5
gilli_1: DrY1_W6
gilli_1: DrY2_F1
gilli_1: DrY2_F2
gilli_1: DrY2_F3
gilli_1: DrY2_F4
gilli_1: DrY2_F5
gilli_1: DrY2_F6
gilli_1: DrY2_N1
gilli_1: DrY2_N2
gilli_1: DrY2_N3
gilli_1: DrY2_N4
0
1000000
2500000
Coverage (reads/bp)
Genome position (bp)

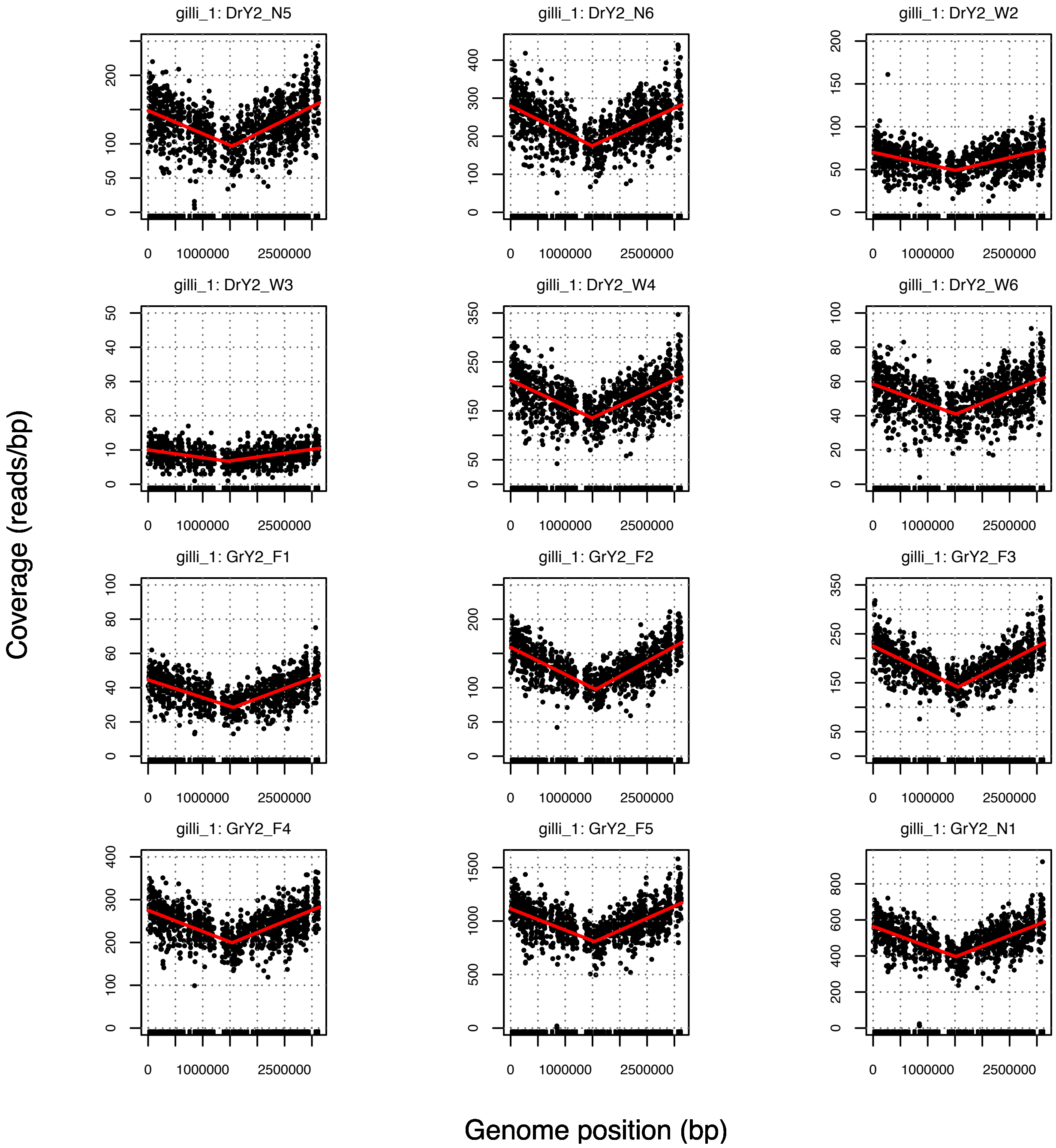

gilli_1: DrY2_N5
gilli_1: DrY2_N6
gilli_1: DrY2_W2
gilli_1: DrY2_W3
gilli_1: DrY2_W4
gilli_1: DrY2_W6
gilli_1: GrY2_F1
gilli_1: GrY2_F2
gilli_1: GrY2_F3
gilli_1: GrY2_F4
gilli_1: GrY2_F5
gilli_1: GrY2_N1
0
1000000
2500000
Coverage (reads/bp)
Genome position (bp)

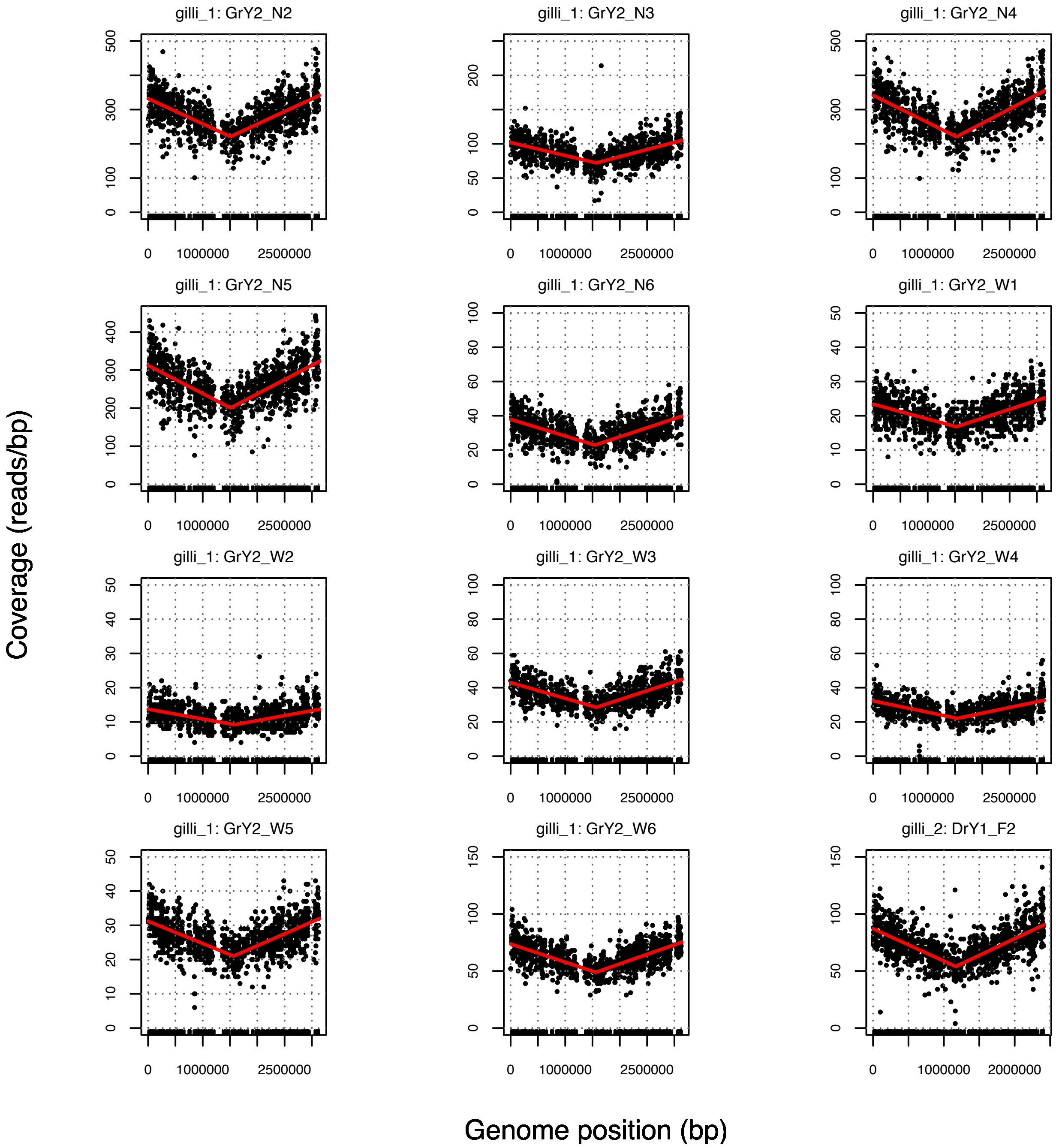
gilli_1: GrY2_N2
gilli_1: GrY2_N3
gilli_1: GrY2_N4
gilli_1: GrY2_N5
gilli_1: GrY2_N6
gilli_1: GrY2_W1
gilli_1: GrY2_W2
gilli_1: GrY2_W3
gilli_1: GrY2_W4
gilli_1: GrY2_W5
gilli_1: GrY2_W6
gilli_2: DrY1_F2
Coverage (reads/bp)
Genome position (bp)

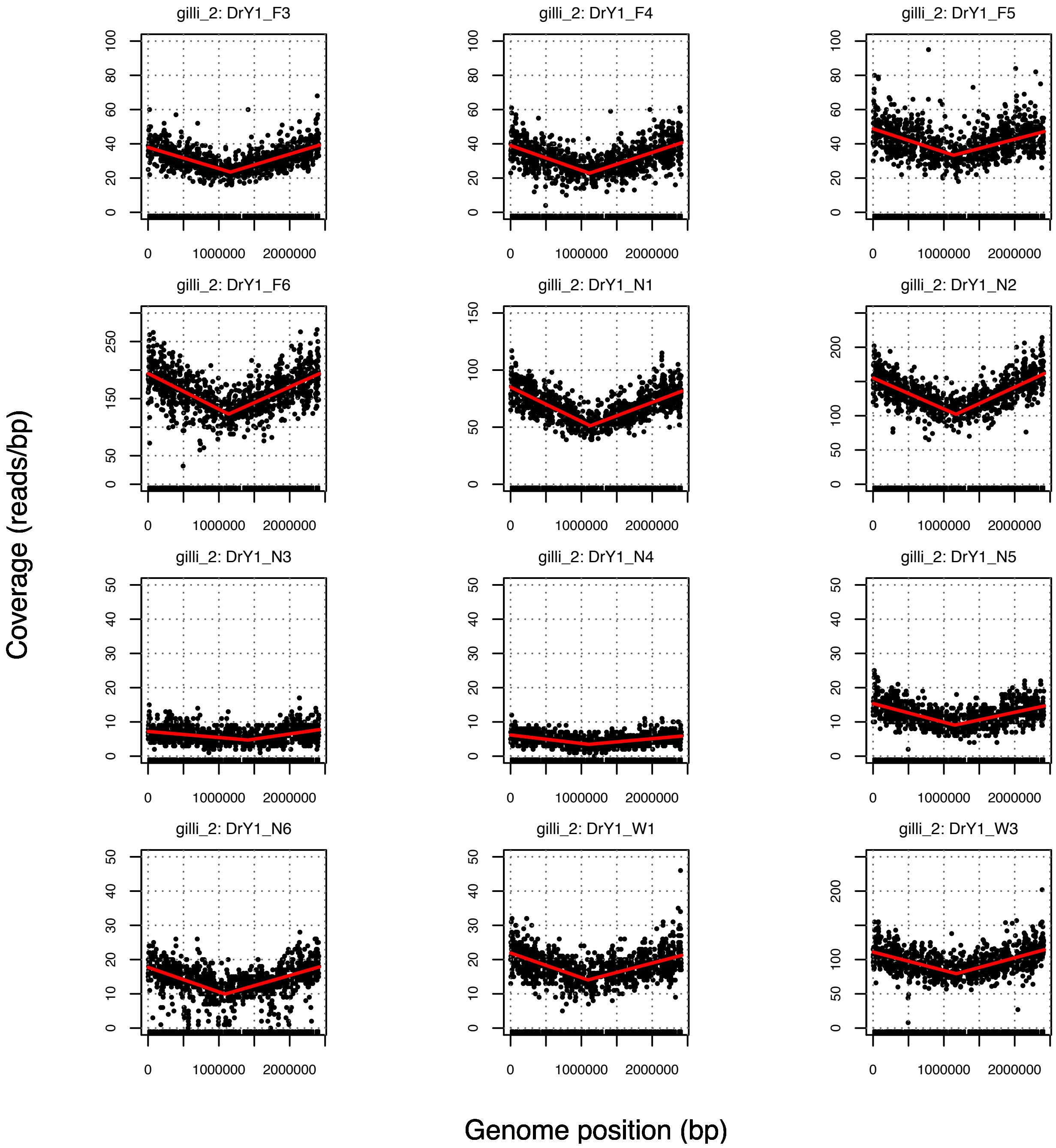

gilli_2: DrY1_F3
gilli_2: DrY1_F4
gilli_2: DrY1_F5
gilli_2: DrY1_F6
gilli_2: DrY1_N1
gilli_2: DrY1_N2
gilli_2: DrY1_N3
gilli_2: DrY1_N4
gilli_2: DrY1_N5
gilli_2: DrY1_N6
gilli_2: DrY1_W1
gilli_2: DrY1_W3
Coverage (reads/bp)
Genome position (bp)

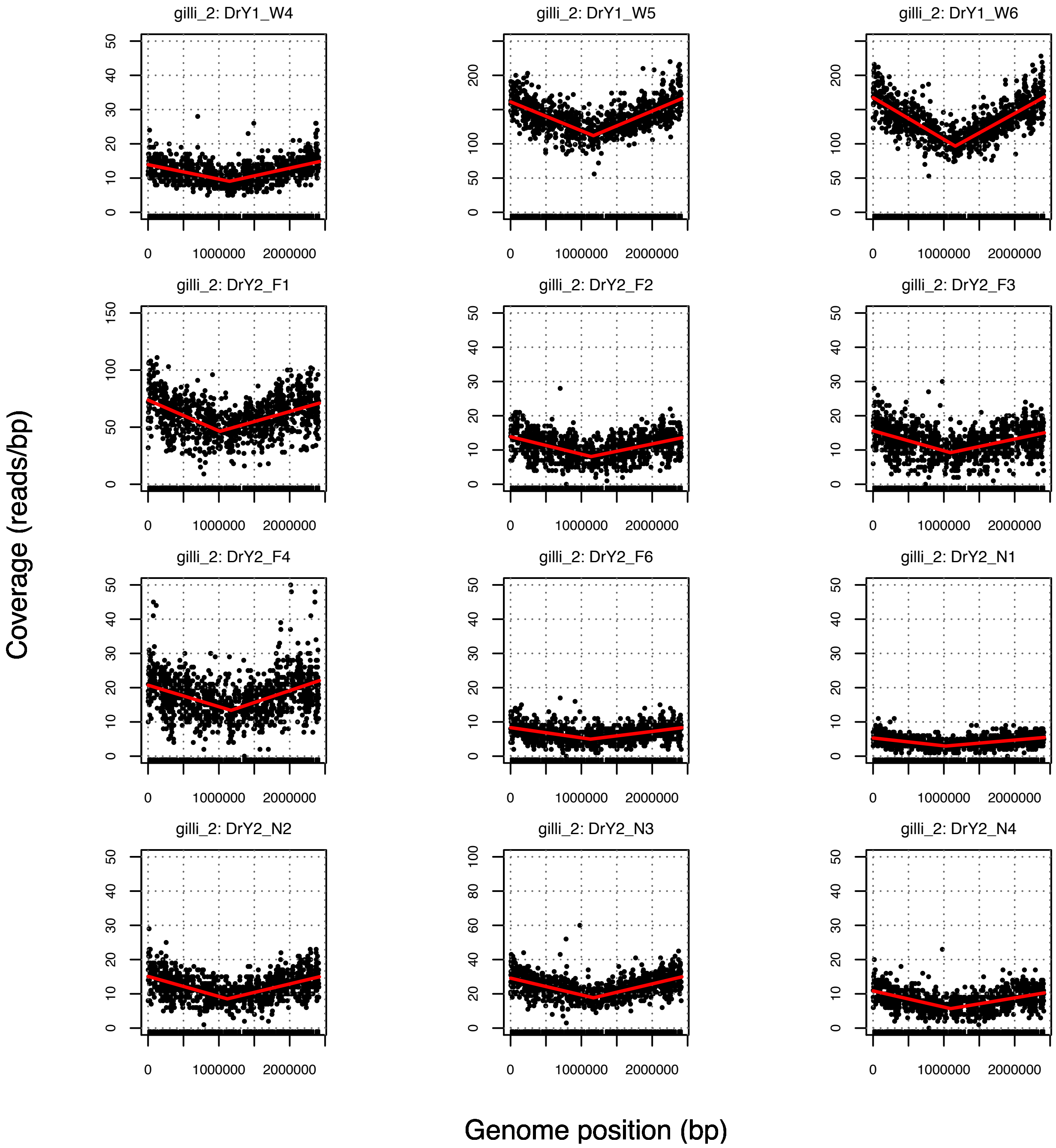
gilli_2: DrY1_W4
gilli_2: DrY1_W5
gilli_2: DrY1_W6
gilli_2: DrY2_F1
gilli_2: DrY2_F2
gilli_2: DrY2_F3
gilli_2: DrY2_F4
gilli_2: DrY2_F6
gilli_2: DrY2_N1
gilli_2: DrY2_N2
gilli_2: DrY2_N3
gilli_2: DrY2_N4
Coverage (reads/bp)
Genome position (bp)
0
1000000
2000000

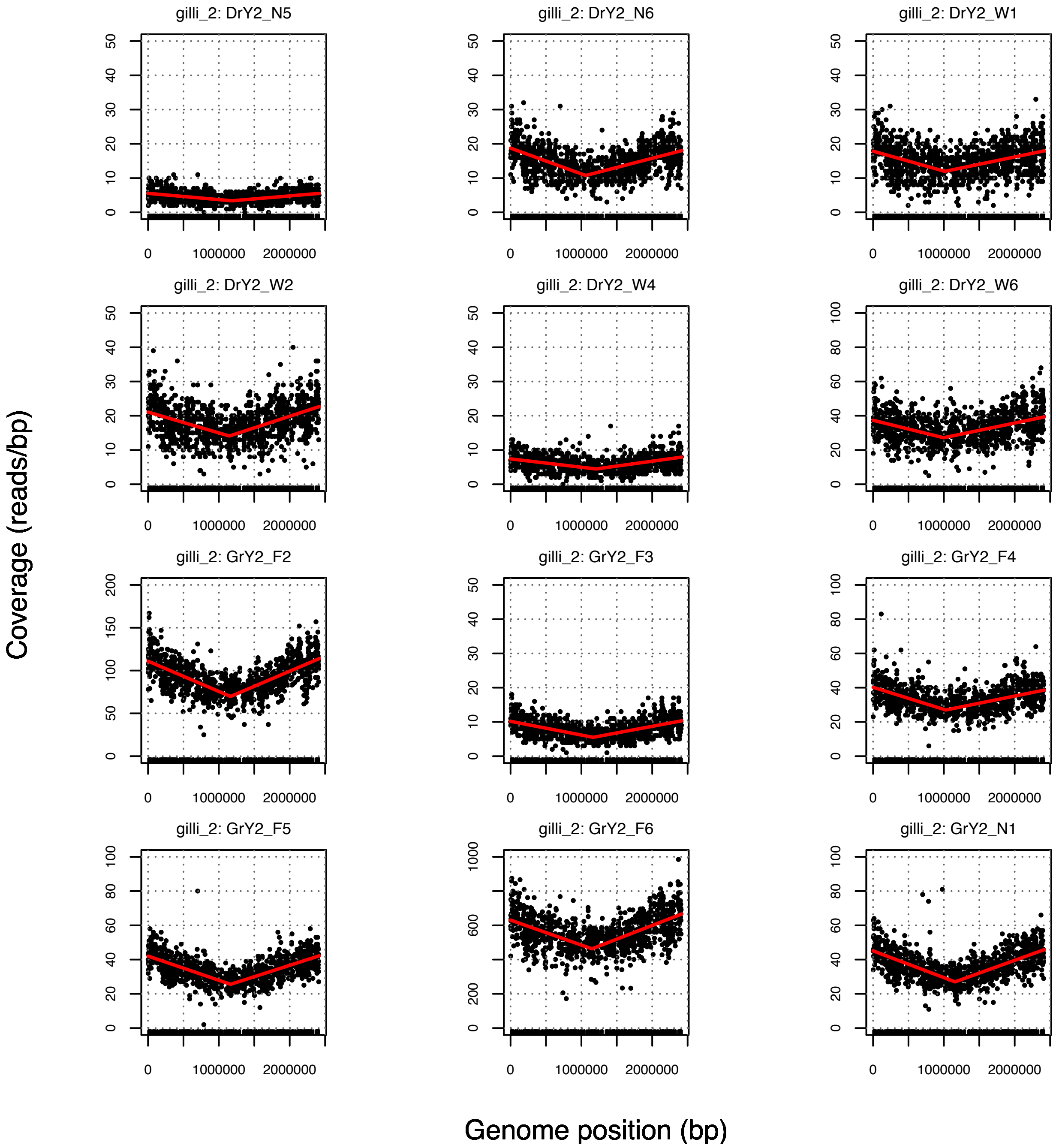
gilli_2: DrY2_N5
gilli_2: DrY2_N6
gilli_2: DrY2_W1
gilli_2: DrY2_W2
gilli_2: DrY2_W4
gilli_2: DrY2_W6
gilli_2: GrY2_F2
gilli_2: GrY2_F3
gilli_2: GrY2_F4
gilli_2: GrY2_F5
gilli_2: GrY2_F6
gilli_2: GrY2_N1
0
1000000
2000000
Coverage (reads/bp)
Genome position (bp)

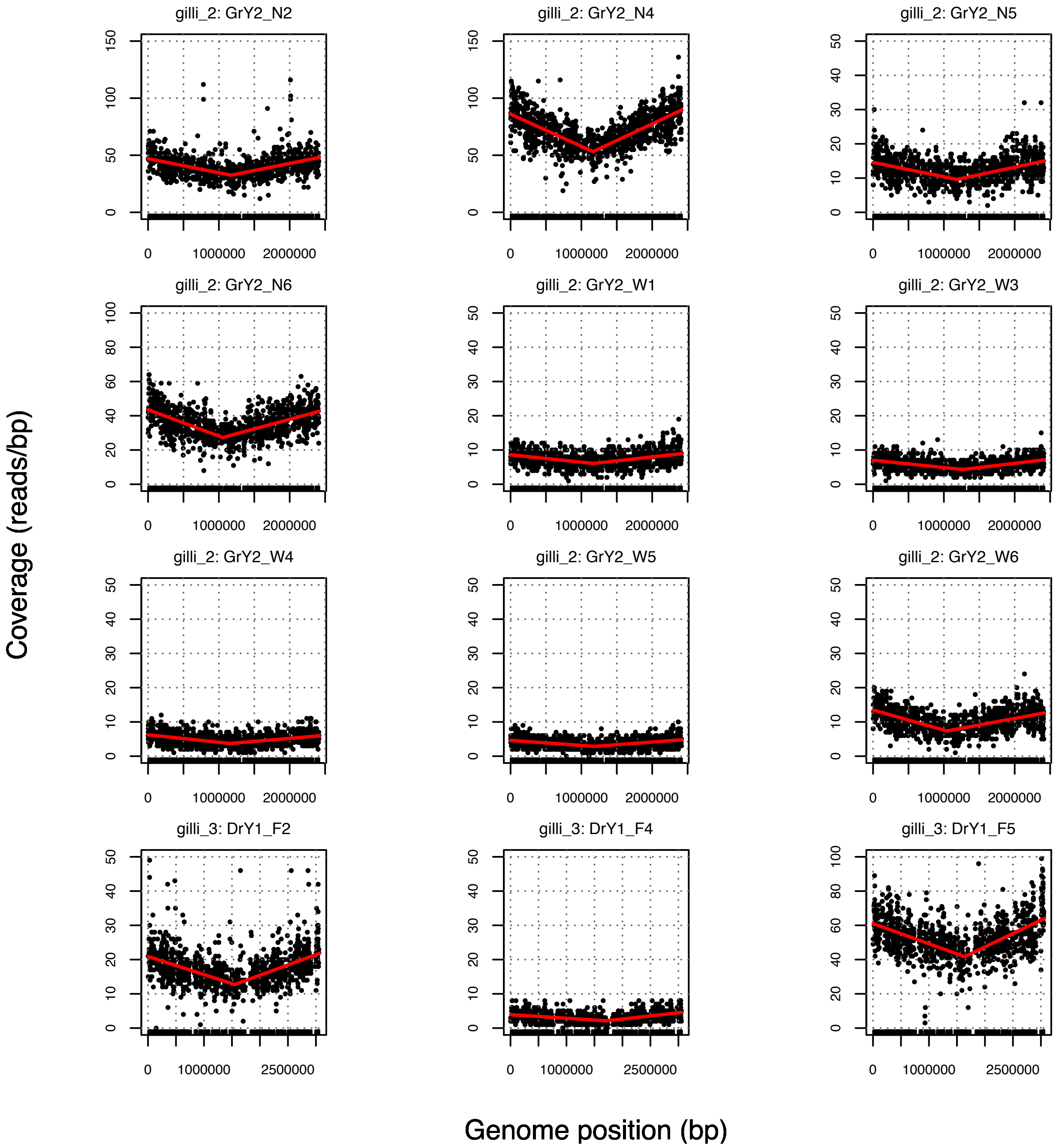
gilli_2: GrY2_N2
gilli_2: GrY2_N4
gilli_2: GrY2_N5
gilli_2: GrY2_N6
gilli_2: GrY2_W1
gilli_2: GrY2_W3
gilli_2: GrY2_W4
gilli_2: GrY2_W5
gilli_2: GrY2_W6
gilli_3: DrY1_F2
gilli_3: DrY1_F4
gilli_3: DrY1_F5
Coverage (reads/bp)
Genome position (bp)

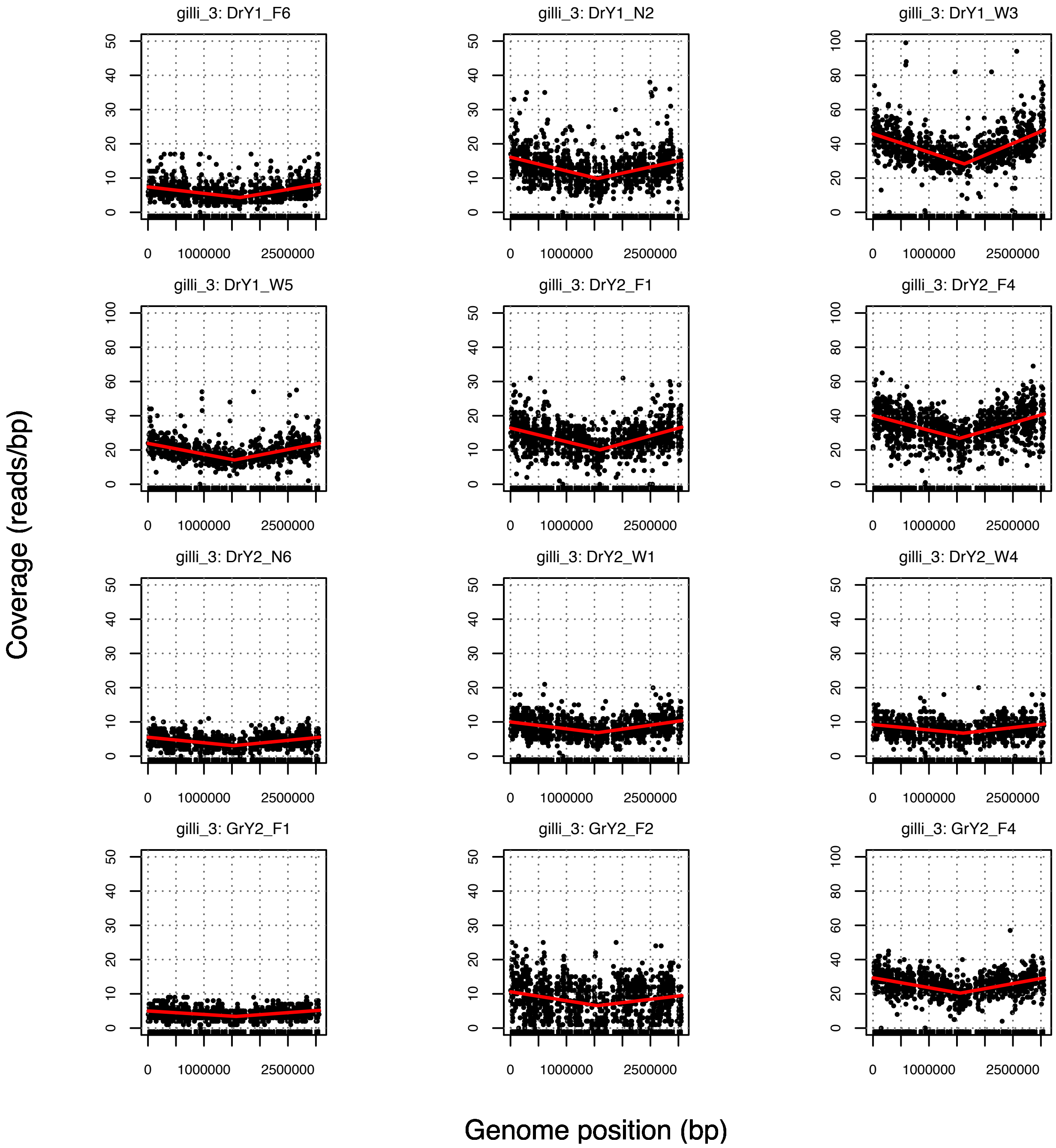
gilli_3: DrY1_F6
gilli_3: DrY1_N2
gilli_3: DrY1_W3
gilli_3: DrY1_W5
gilli_3: DrY2_F1
gilli_3: DrY2_F4
gilli_3: DrY2_N6
gilli_3: DrY2_W1
gilli_3: DrY2_W4
gilli_3: GrY2_F1
gilli_3: GrY2_F2
gilli_3: GrY2_F4
Coverage (reads/bp)
Genome position (bp)
0 1000000 2500000

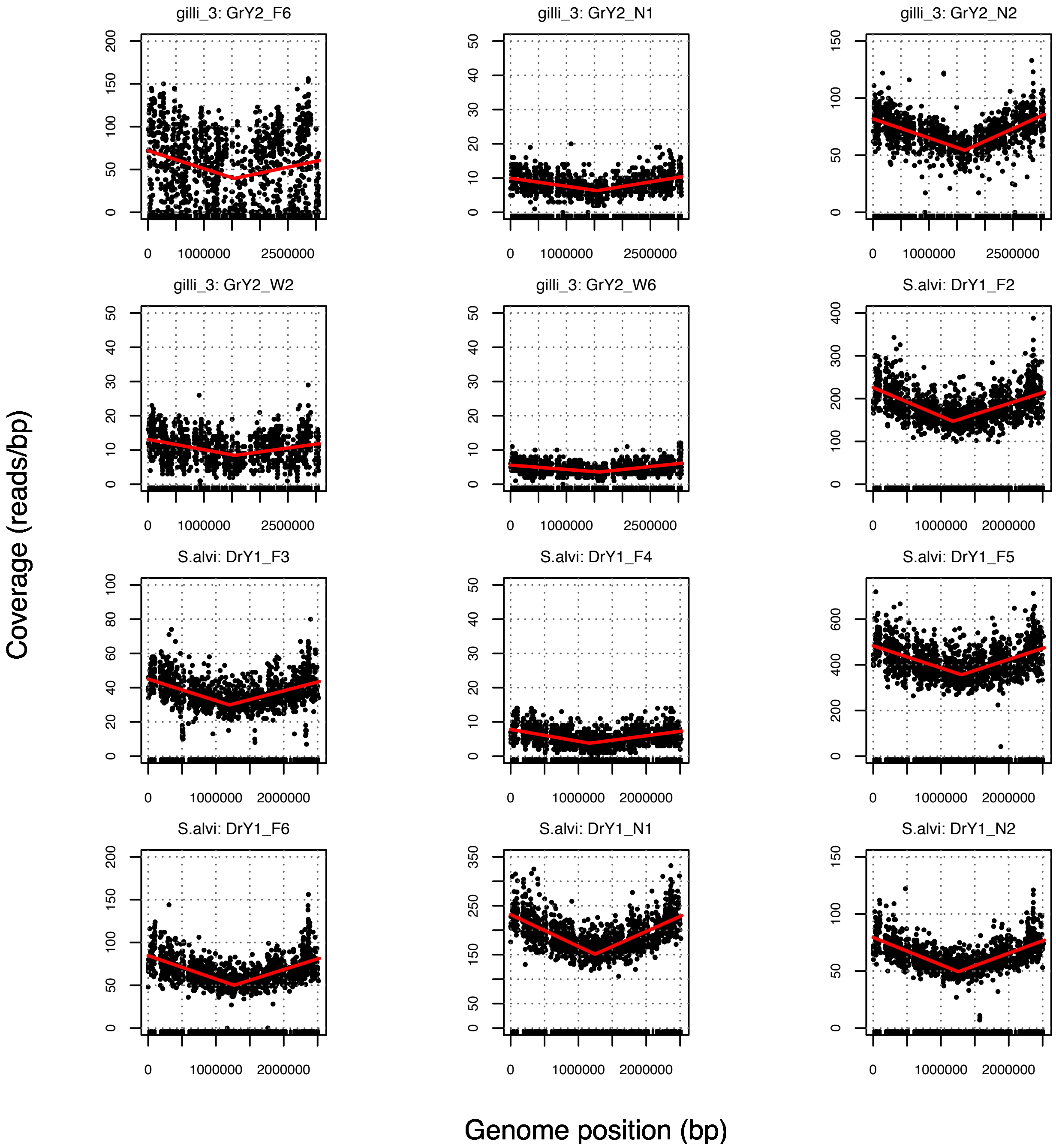
gilli_3: GrY2_F6
gilli_3: GrY2_N1
gilli_3: GrY2_N2
gilli_3: GrY2_W2
gilli_3: GrY2_W6
S.alvi: DrY1_F2
S.alvi: DrY1_F3
S.alvi: DrY1_F4
S.alvi: DrY1_F5
S.alvi: DrY1_F6
S.alvi: DrY1_N1
S.alvi: DrY1_N2
Coverage (reads/bp)
Genome position (bp)

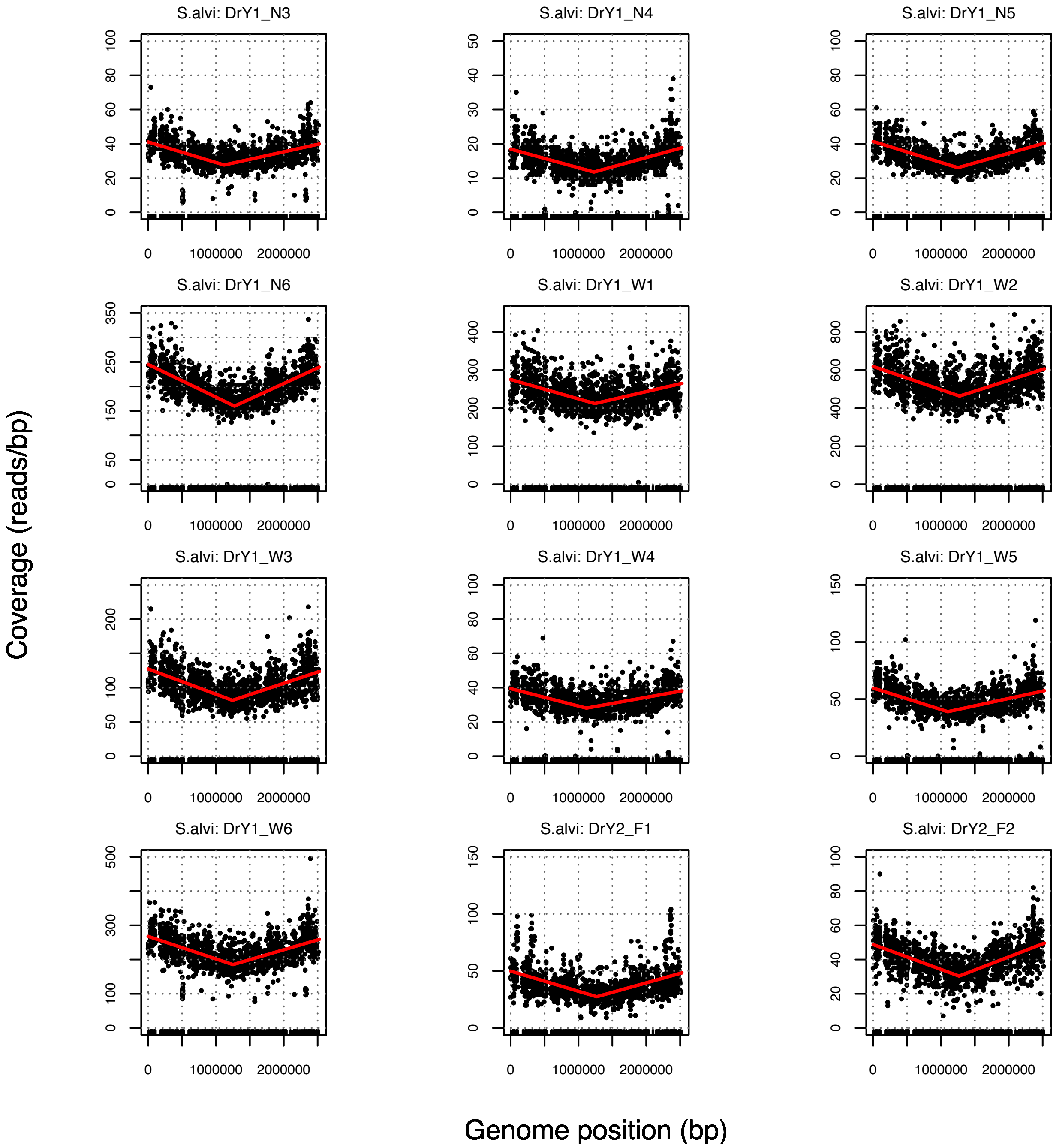
S.alvi: DrY1_N3
S.alvi: DrY1_N4
S.alvi: DrY1_N5
S.alvi: DrY1_N6
S.alvi: DrY1_W1
S.alvi: DrY1_W2
S.alvi: DrY1_W3
S.alvi: DrY1_W4
S.alvi: DrY1_W5
S.alvi: DrY1_W6
S.alvi: DrY2_F1
S.alvi: DrY2_F2
Coverage (reads/bp)
Genome position (bp)
0
1000000
2000000

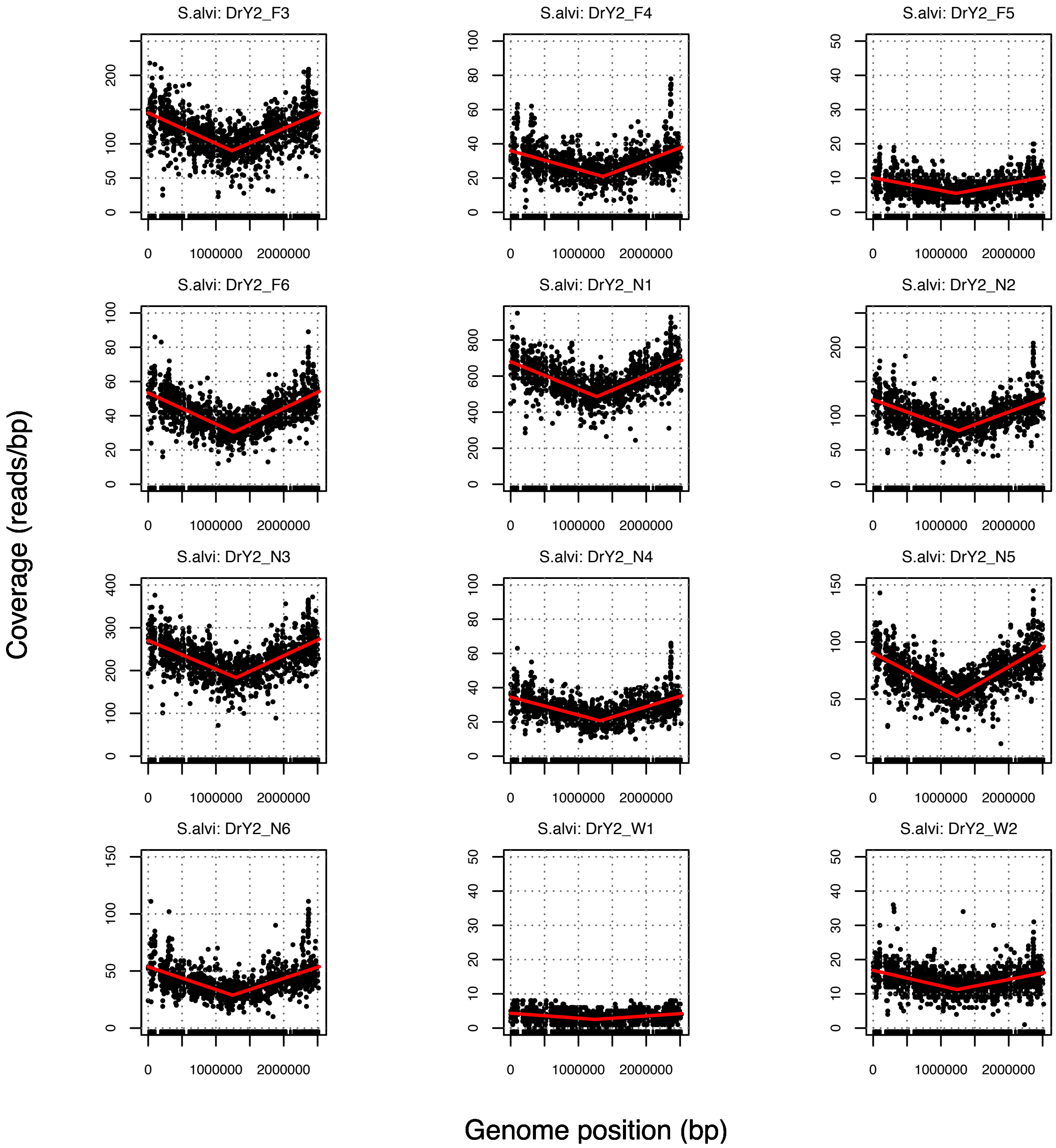
S.alvi: DrY2_F3
S.alvi: DrY2_F4
S.alvi: DrY2_F5
S.alvi: DrY2_F6
S.alvi: DrY2_N1
S.alvi: DrY2_N2
S.alvi: DrY2_N3
S.alvi: DrY2_N4
S.alvi: DrY2_N5
S.alvi: DrY2_N6
S.alvi: DrY2_W1
S.alvi: DrY2_W2
0
1000000
2000000
Coverage (reads/bp)
Genome position (bp)

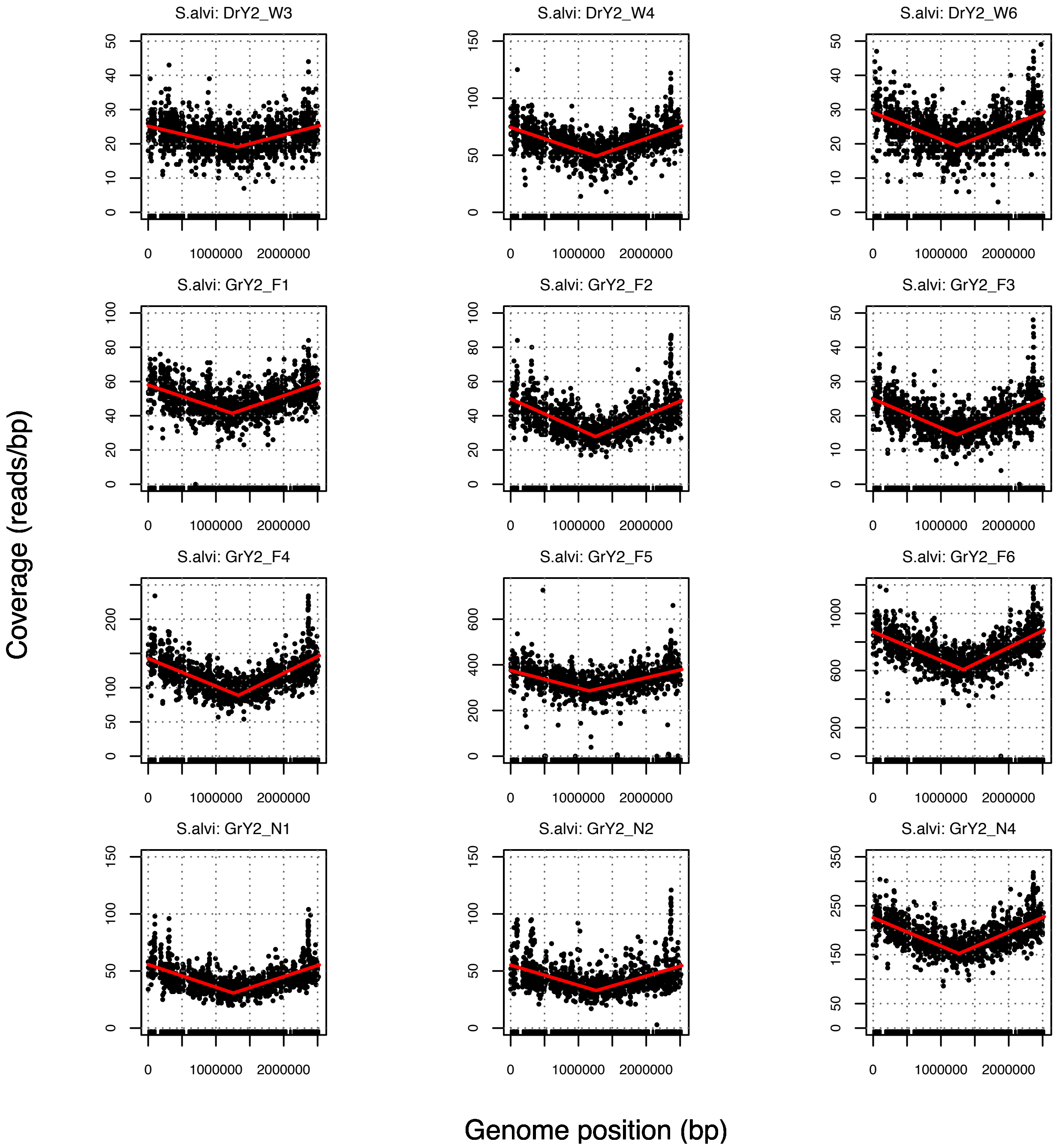
S.alvi: DrY2_W3
S.alvi: DrY2_W4
S.alvi: DrY2_W6
S.alvi: GrY2_F1
S.alvi: GrY2_F2
S.alvi: GrY2_F3
S.alvi: GrY2_F4
S.alvi: GrY2_F5
S.alvi: GrY2_F6
S.alvi: GrY2_N1
S.alvi: GrY2_N2
S.alvi: GrY2_N4
Coverage (reads/bp)
Genome position (bp)
0
1000000
2000000

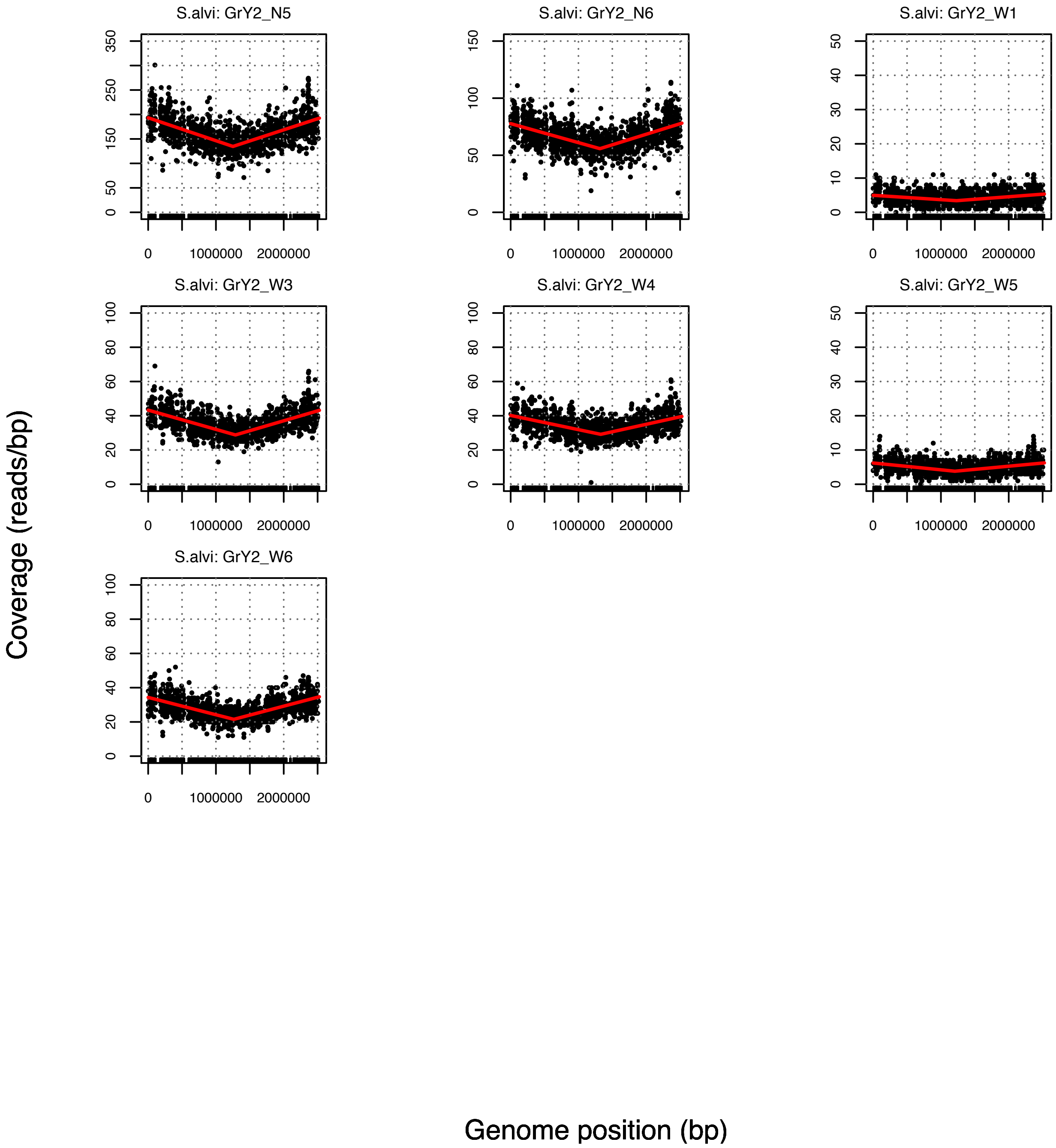
S.alvi: GrY2_N5
S.alvi: GrY2_N6
S.alvi: GrY2_W1
S.alvi: GrY2_W3
S.alvi: GrY2_W4
S.alvi: GrY2_W5
S.alvi: GrY2_W6
Coverage (reads/bp)
Genome position (bp)

Supplementary Figure 8. Coverage plots for all SDPs/phylotypes within all samples. For each SDP (or phylotype, in the absence of confirmed SDPs), each dot represents the summed mapped read coverage of a core gene family, plotted relative the location within a reference genome. Note that *Commensalibacter* sp. is currently only represented by a single un-ordered draft genome, and is therefore not included.

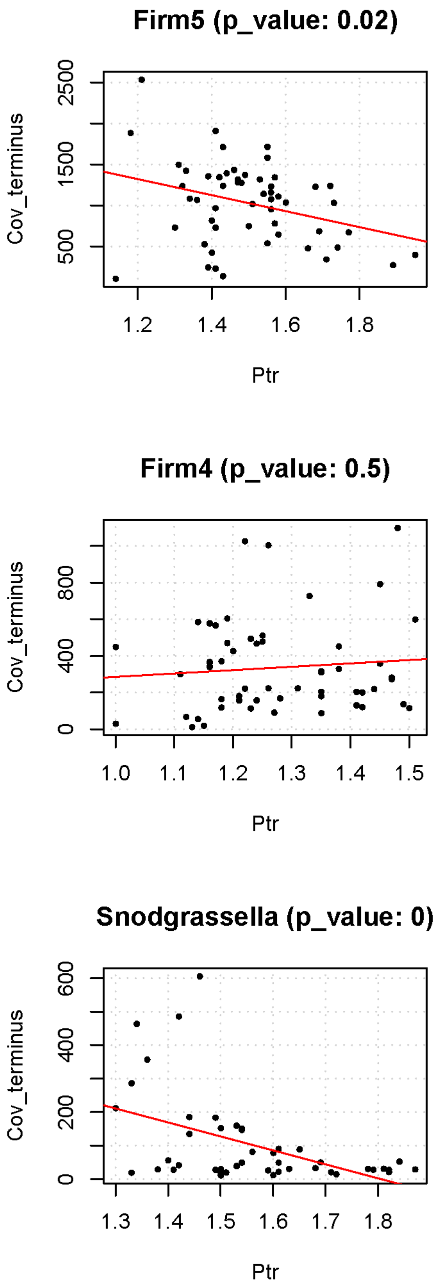

Supplementary Figure 9. Correlation plots of PTR and terminus coverage. For each core phylotype, data points within the plot display the PTR (x-axis) and terminus coverage (y-axis) of the metagenomic samples. The p-values given in the title of each plot were derived from the correlation test function in R ("cor.test"). Although some test results were significant, (suggesting a negative correlation), the spread within the plots indicate that the association is weak.

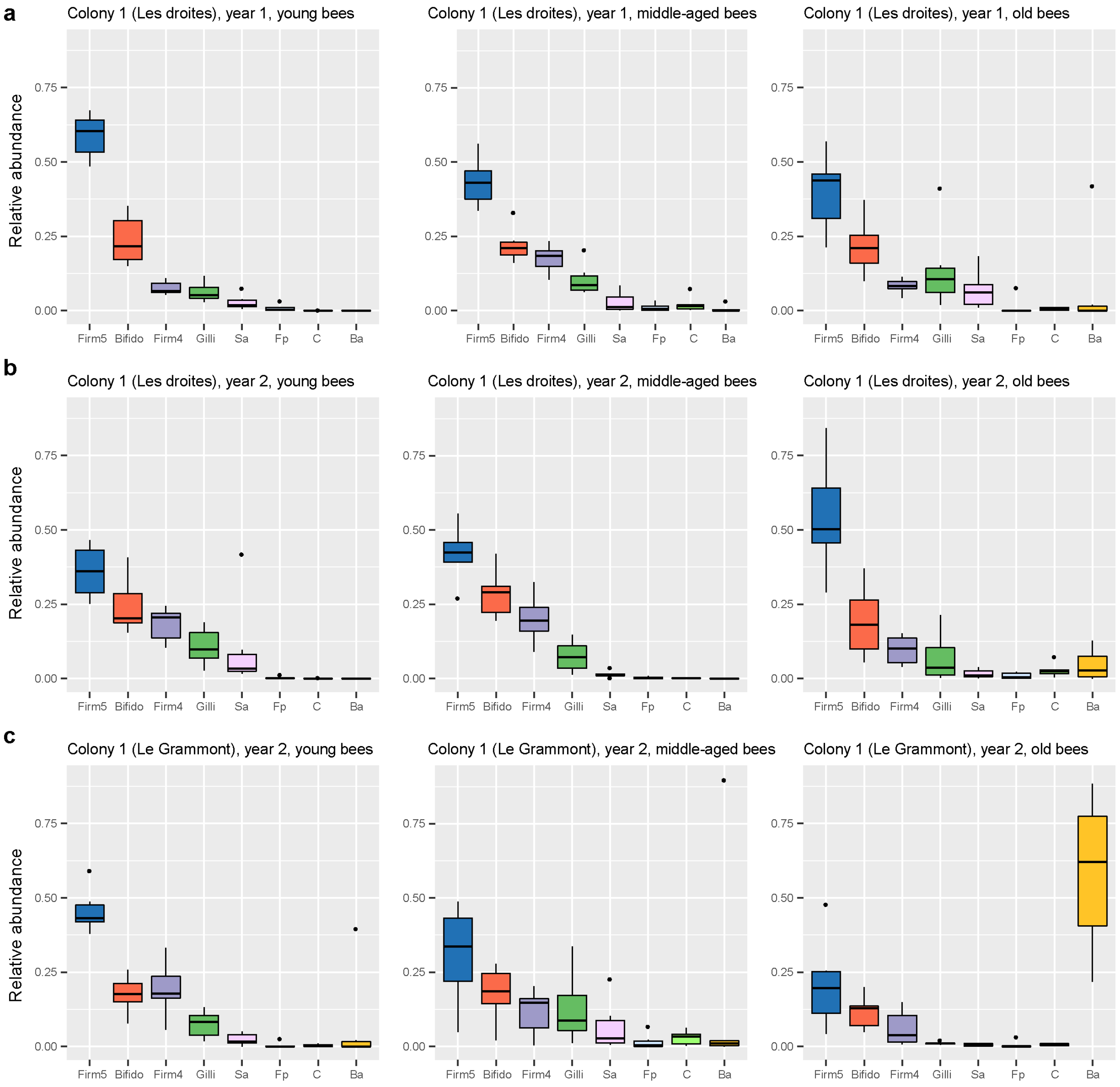

Supplementary Figure 10. Relative abundances of phylotypes for each sampling. Each panel represents a sampling time-point per colony. Colony 1 was sampled in two consecutive years (**a-b**). Colony 2 was sampled only in the second year (**c**)**.** For the boxplots, the black centre line displays the median, the boxes correspond to the 25th and 75th percentiles, and whiskers extend to the most extreme data points that are within the 1.5 interquartile range of the box. For each sample, the relative abundance of the five core phylotypes and three non-core phylotypes was calculated. Phylotype abbreviations: Gilli: Gilliamella, Sa: *Snodgrassella alvi*, Fp: *Frischella perrara*, C: *Commensalibacter* sp, Ba: *Bartonella apis*.

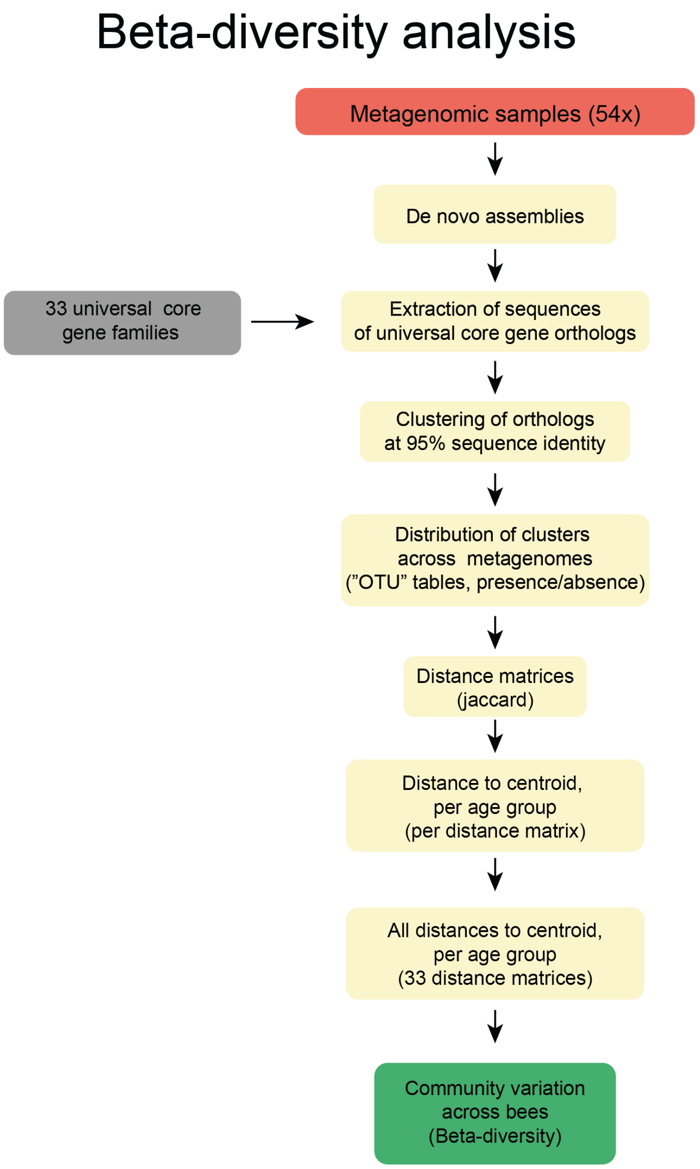

Supplementary Figure 11. Flowchart of the analysis of beta-diversity relative to age, including non-core members. Orthologs of 33 universal core gene families were extracted from the *de novo* assembly of each metagenomic sample. For each gene family, the sequences were clustered at 95% nucleotide identity. The occurrence of each cluster was inferred based on the cluster members (samples for which the corresponding "OTU" was present are expected to have a member in the cluster), and used to generate an "OTU" table with presence/absence data. Finally, the OTU tables were converted to jaccard distances, based on which the distance to the estimated centroid for each age group was calculated. To obtain a single estimate from all 33 gene-families, the "distance-to-centroid" values for each gene-family was pooled, and significant differences between groups was tested with one-way ANOVA. The flowchart colors are the same as for Supplementary Figure 1.

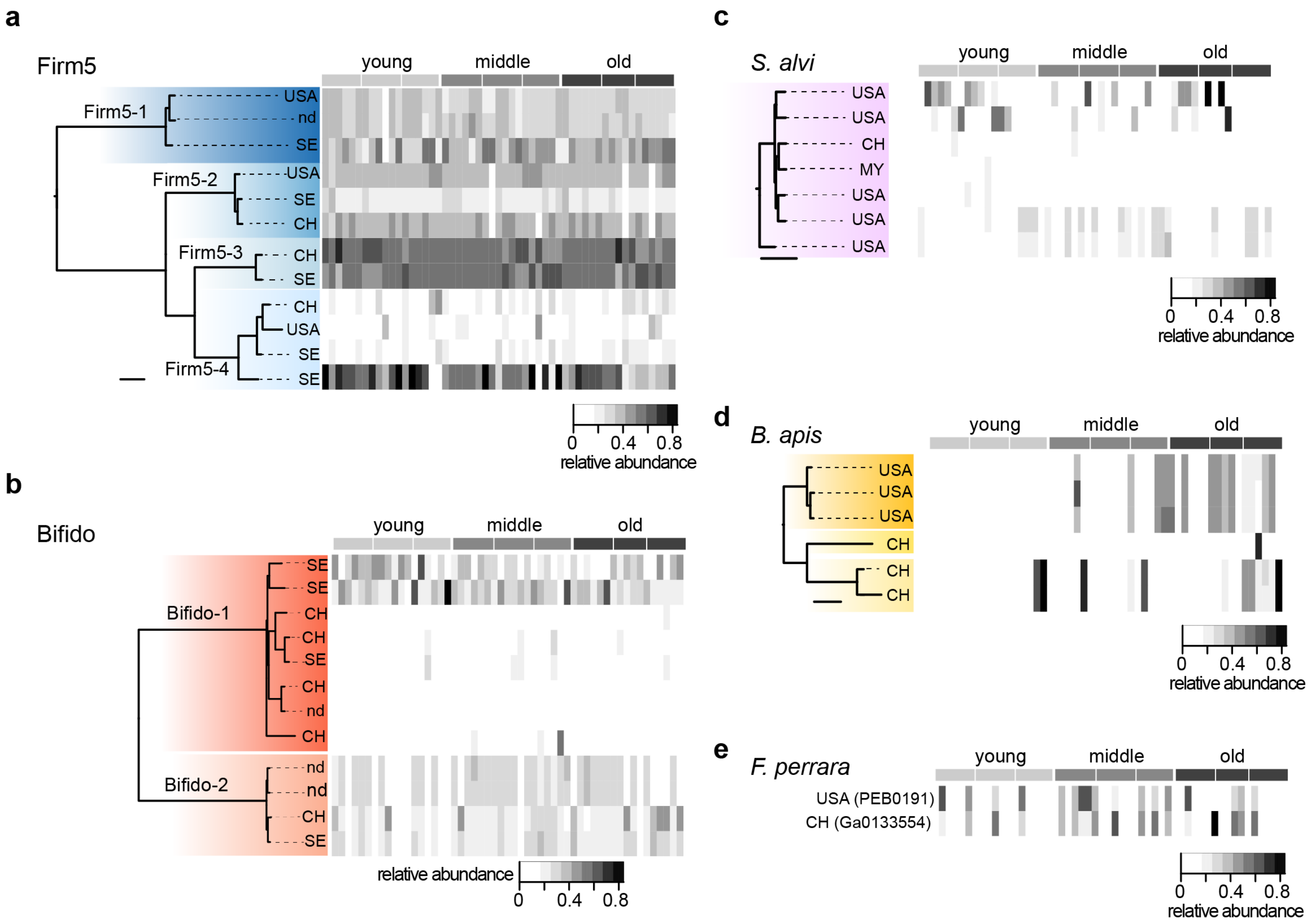

Supplementary Figure 12. Heatmap of read recruitment on genomes in the reference database. Proportion of metagenomic reads mapping to core genes in reference database genomes within each SDP (or phylotype, in the absence of confirmed SDPs). Read recruitment heatmap for Gilliamella is shown in Figure 5a. Each vertical bar in the heatmap corresponds to a single sample. The phylogenetic tree given to the left of the SDP clusters is the same as in Figure 2. Node labels indicate geographic origin of genomes (CH: Switzerland, USA: United States of America, NO: Norway, SE: Sweden, MY: Malaysia, nd: not determined).

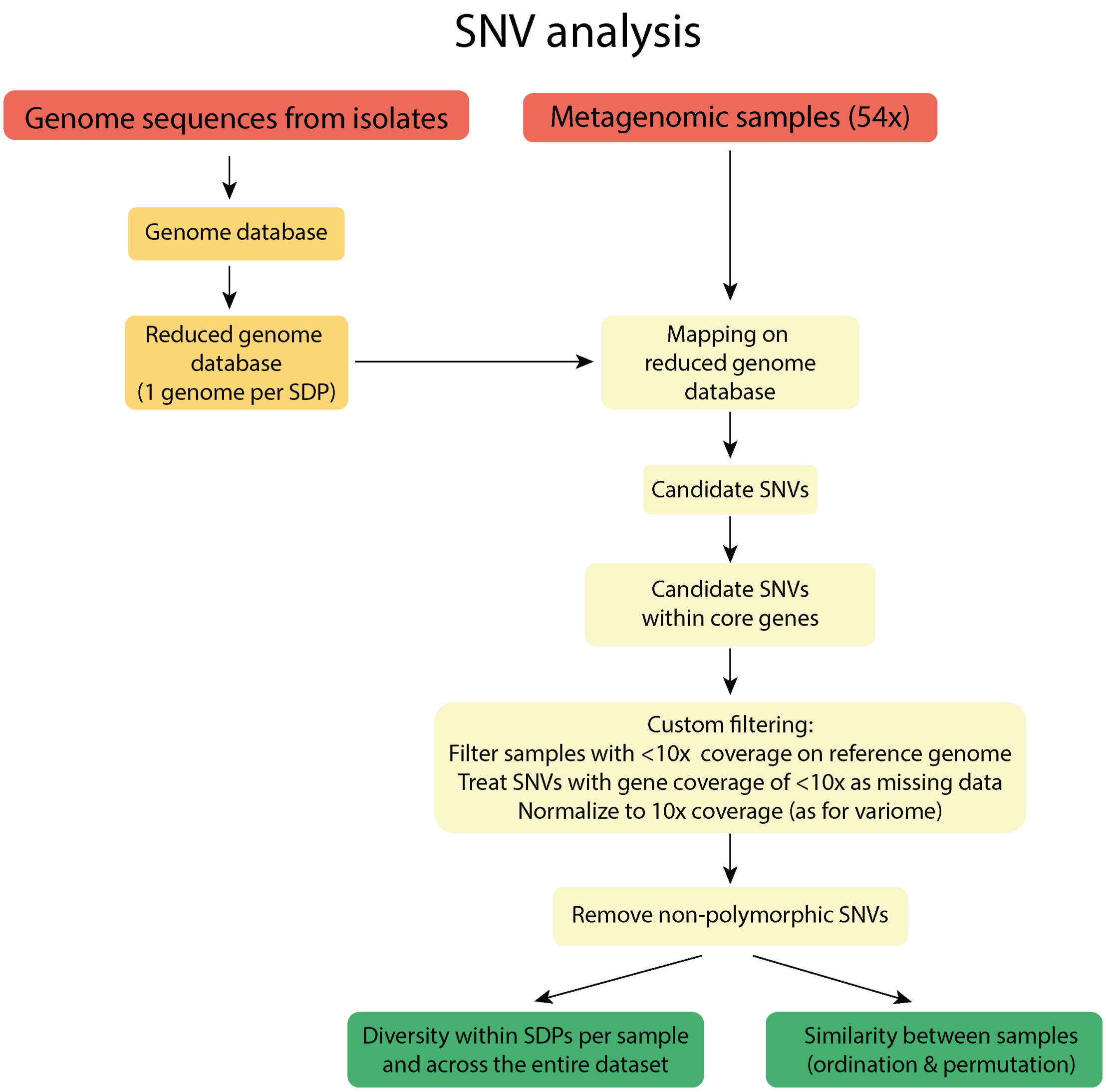

Supplementary Figure 13. Flowchart of the single nucleotide variant (SNV) analysis to determine strain diversity within SDPs. Metagenomic reads of each sample were mapped onto a reduced version of the reference genome database comprising only one genome per SDP. Mapping all reads from a given SDP onto the same reference genome facilitated downstream SNV identification and quantification. Candidate SNVs were identified with metaSNV[50] using default settings. From the total set of candidates, SNVs occurring within core genes were extracted (i.e. single copy genes present in each genome of a phylotype in the original reference database). Next, a second filtering was applied according to the depicted criteria. The normalization allowed us to compare strain-level diversity within SDPs between individual samples and the entire dataset and to test for similarity in strain-level diversity between bees of the same age and from the same colony. The flowchart colors are the same as for Supplementary Figure 1.

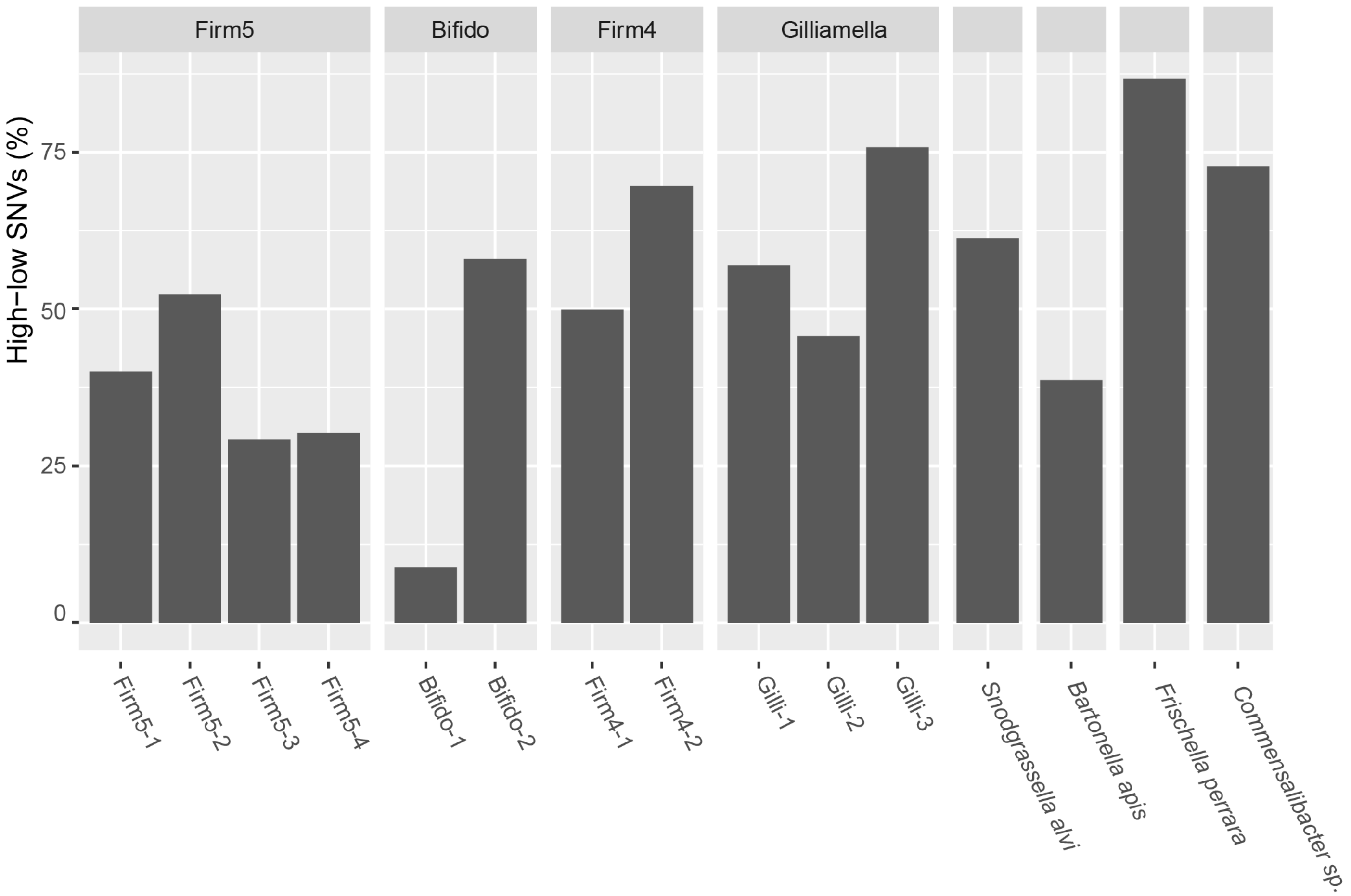

Supplementary Figure 14. Fractions of SNVs occurring at both high and low relative intra-sample abundances across the study. SNVs were classified as "high-low" SNVs if they occurred with an intra-sample relative abundance of 100% in at least one sample, and less than 10% in at least one other sample. The barplot displays the fractions of SNVs falling within this category, for each SDP/phylotype.

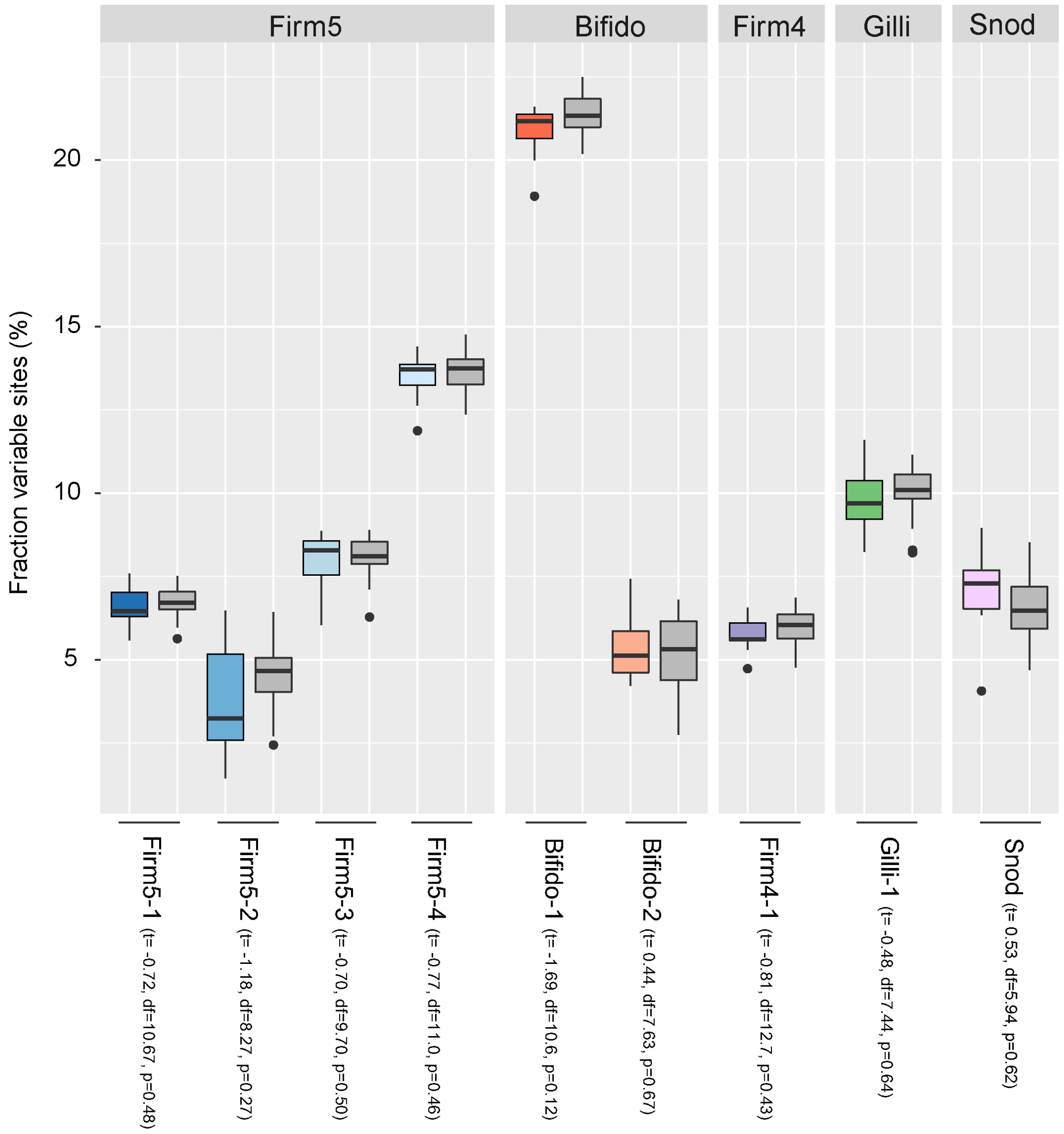

Supplementary Figure 15. Fraction variable sites in samplings versus random subsets of six bees. The boxplots display the total fraction of variable sites, calculated for each of the nine samplings (6 bees per sample, see Figure 1b), and for 27 random combinations of six bees. Numbers for real samplings are displayed with colored boxes, while numbers corresponding to random subsets are shown with grey boxes. For each boxplot, the centre line displays the median, the boxes correspond to the 25th and 75th percentiles, and whiskers extend to the most extreme data-points that are within the 1.5 interquartile range of the box. Note, the analysis was restricted to SDPs/phylotypes that were confidently detected (at least 10X terminus coverage) in at least five bees, for at least six samplings. Results of Welch's t-test are shown in parenthesis on the x-axis labels.

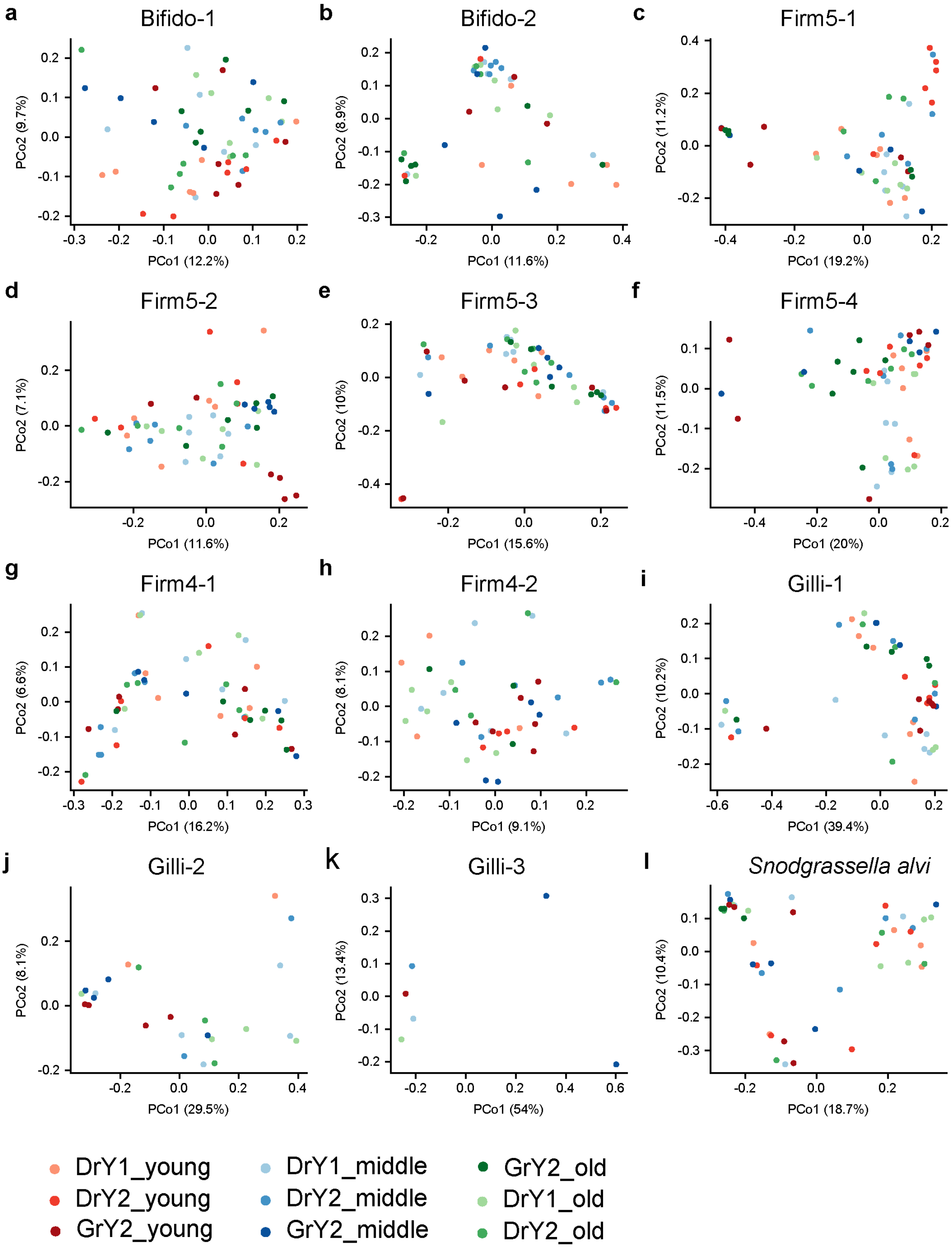

Supplementary Figure 16. Principal coordinate analysis on the Jaccard distances calculated based on shared SNVs. Different colors indicate age, colony origin, and sampling year. The number of samples included in the analysis varies between SDPs, because only samples with a reference genome terminus coverage of at least 10x were included in the analysis.

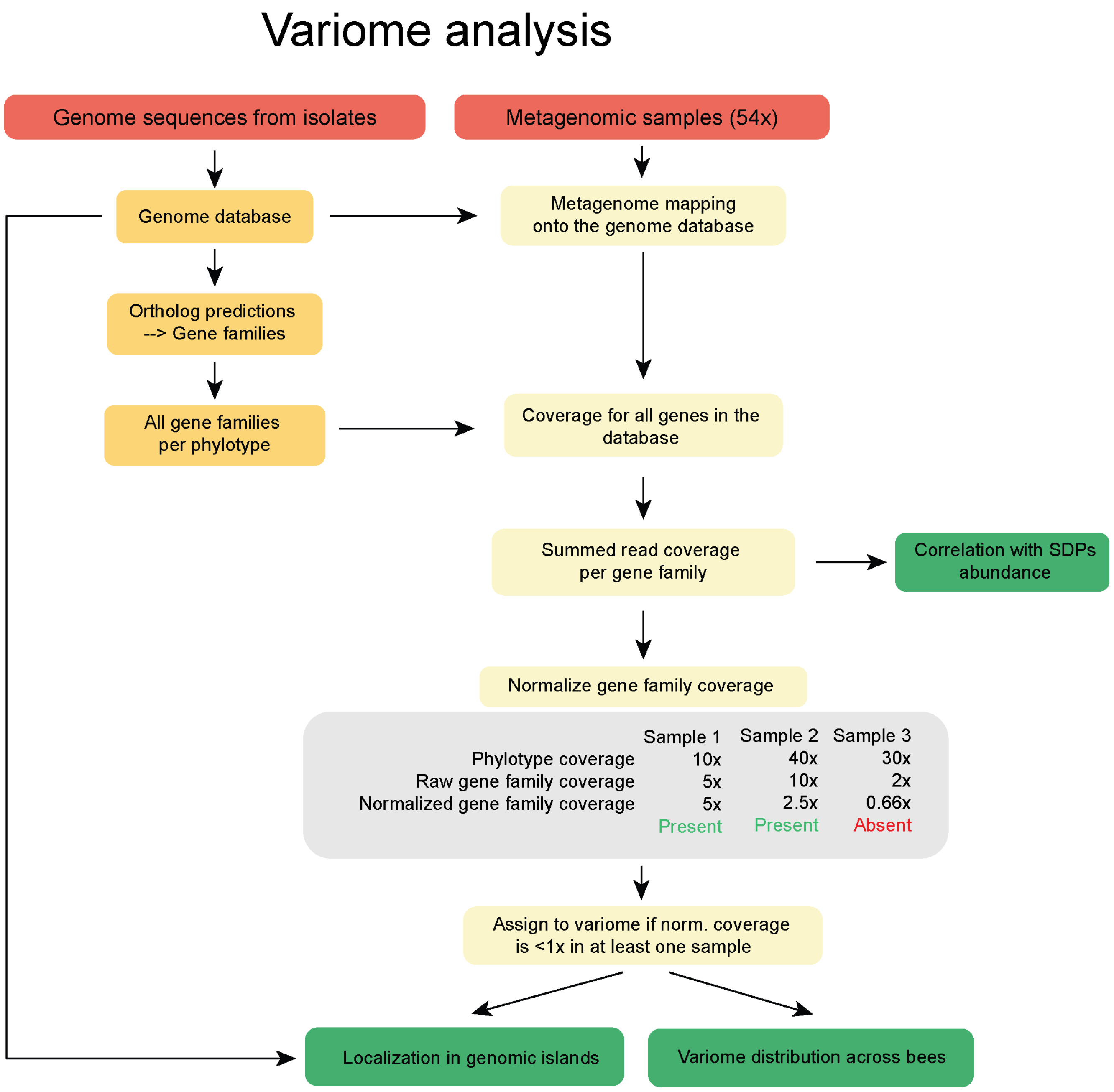

Supplementary Figure 17. Flowchart of the variome analysis to identify gene families variably associated with phylotypes, and their distribution across individual bees. Metagenomic reads were mapped to the reference database and the read coverage extracted for all genes. Read coverages of homologous genes are summed over all gene family members per phylotype, and subsequently normalized to 10x phylotype coverage as explained in the figure (grey box). A gene family was assigned to the variome, if the normalized read coverage was <1x in at least one metagenomic sample. The flowchart colors are the same as for Supplementary Figure 1.

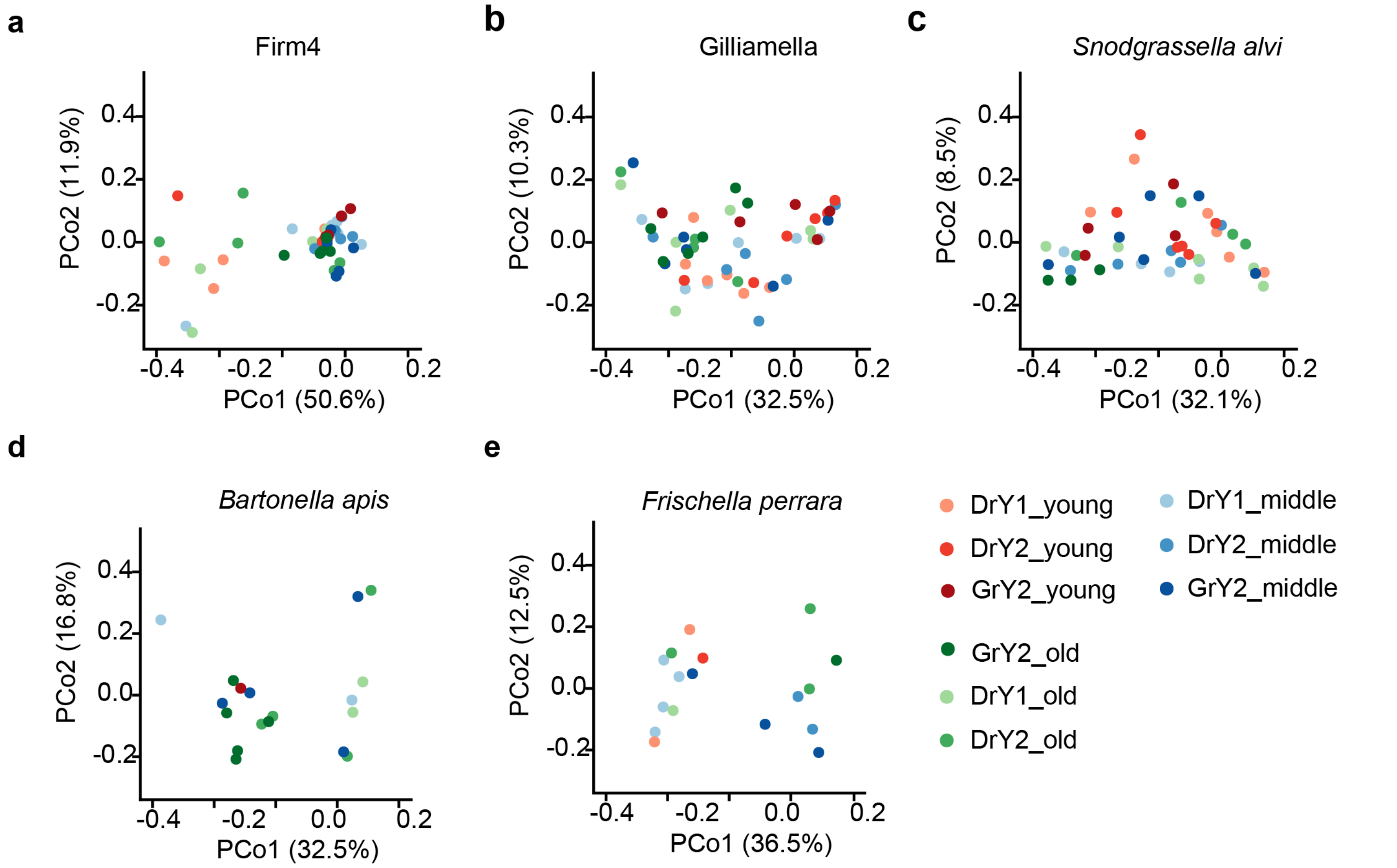

Supplementary Figure 18. Principal coordinate analysis on Jaccard distances, calculated based on shared variome gene content. Results are shown for the five phylotypes that are not shown in Figure 6**.** Different shades of each color indicate colony origin and sampling year combination.

Supplementary Tables

Supplementary Table 1. Community compositional changes at the phylotype-level analyzed with generalized linear models.

Phylotype
Model: data ~ lifestage*hive, family=negative binomial

Multivariate test:

	Res.Df	Df.diff	Dev	Pr(>Dev)	
(Intercept)	52				
full_data$Lifestage	50	2	44.39	0.003	**
full_data$Hive	49	1	40.21	0.001	***
full_data$Lifestage:full_data$Hive	47	2	55.96	0.001	***
			140.56		

Univariate tests, Dev	Firm5	Bifido	Firm4	Gilliamella	S. alvi	B. apis	F. perrara	Commensalibacter sp.
full_data$Lifestage	2.384	2.593	14.175	2.238	1.14	4.403	0.675	16.783
full_data$Hive	8.346	11.157	0.36	1.69	0.566	17.929	0.105	0.059
full_data$Lifestage:full_data$Hive	7.712	1.244	5.916	15.866	12.085	3.732	1.419	7.988

Univariate tests, p-value	Firm5	Bifido	Firm4	Gilliamella	S. alvi	B. apis	F. perrara	Commensalibacter sp.
full_data$Lifestage	0.817	0.817	0.011	0.817	0.854	0.522	0.854	0.007
full_data$Hive	0.056	0.015	0.936	0.703	0.936	0.002	0.937	0.937
full_data$Lifestage:full_data$Hive	0.175	0.827	0.314	0.011	0.047	0.581	0.827	0.171

Compositional changes in relative abundances of phylotypes and SDPs were tested with generalized linear models (ManyGLM), using the R package "mvabund"[3], and the negative binomial distribution. The total parameter deviance for each model is summed for the "Dev" column, and the contribution to the total is shown for each member in the sections "Univariate tests, Dev". Significant changes (p<0.01) as estimated by re-sampling of the test-statistic are highlighted in color.

Supplementary Table 2. Community compositional changes at the SDP-level analyzed with generalized linear models.

SDP
Model: data ~ lifestage*hive, family=negative binomial

Multivariate test:	Res.Df	Df.diff	Dev	Pr(>Dev)	
(Intercept)	53				
full_data$Lifestage	51	2	126.2	0.001	***
full_data$Hive	50	1	54.2	0.001	***
full_data$Lifestage:full_data$Hive	47	2	82.1	0.001	***
			262.5		

Univariate tests, Dev	Firm5_1	Firm5_2	Firm5_3	Firm5_4	Bifido_1	Bifido_2	Firm4_1	Firm4_2	Gilli_1	Gilli_2	Gilli_3
full_data$Lifestage	30.295	9.263	1.132	0.473	11.436	19.674	11.11	15.94	1.382	2.602	0.002
full_data$Hive	3.731	0.184	8.395	4.174	6.082	5.814	0.526	1.322	2.689	0.041	1.329
full_data$Lifestage:full_data$Hive	3.565	0.548	2.455	5.184	0.175	8.352	5.423	6.104	12.757	8.254	3.936

Univariate tests, p-value	Firm5_1	Firm5_2	Firm5_3	Firm5_4	Bifido_1	Bifido_2	Firm4_1	Firm4_2	Gilli_1	Gilli_2	Gilli_3
full_data$Lifestage	0.001	0.132	0.992	0.992	0.066	0.003	0.07	0.007	0.992	0.921	0.992
full_data$Hive	0.485	0.987	0.075	0.437	0.21	0.23	0.979	0.895	0.645	0.987	0.895
full_data$Lifestage:full_data$Hive	0.753	0.946	0.801	0.582	0.946	0.288	0.581	0.492	0.056	0.288	0.753

Univariate tests, Dev	*S. alvi*	*B. apis*	*F. perrara*	*Commensalibacter sp.*
full_data$Lifestage	1.07	4.372	0.675	16.783
full_data$Hive	0.575	19.172	0.105	0.059
full_data$Lifestage:full_data$Hive	12.138	3.803	1.419	7.988

Univariate tests, p-value	*S. alvi*	*B. apis*	*F. perrara*	*Commensalibacter sp.*
full_data$Lifestage	0.992	0.635	0.992	0.007
full_data$Hive	0.979	0.001	0.987	0.987
full_data$Lifestage:full_data$Hive	0.076	0.753	0.907	0.288

Compositional changes in relative abundances of phylotypes and SDPs were tested with generalized linear models (ManyGLM), using the R package "mvabund"[3], and the negative binomial distribution. The total parameter deviance for each model is summed for the "Dev" column, and the contribution to the total is shown for each member in the sections "Univariate tests, Dev". Significant changes (p<0.01) as estimated by re-sampling of the test-statistic are highlighted in color.

Supplementary References

1 Engel, P., Martinson, V. G. & Moran, N. A. Functional diversity within the simple gut microbiota of the honey bee. *Proc Natl Acad Sci U S A* **109**, 11002-11007, doi:10.1073/pnas.1202970109 (2012).

2 Costea, P. I. *et al.* metaSNV: A tool for metagenomic strain level analysis. *PLoS One* **12**, e0182392, doi:10.1371/journal.pone.0182392 (2017).

3 Wang, Y., Naumann, U., Wright, S. T. & Warton, D. I. mvabund- an R package for model-based analysis of multivariate abundance data. *Methods Ecol Evol* **3**, 471-474, doi:10.1111/j.2041-210X.2012.00190.x (2012).
