# Supplementary material for: Genomic diversity landscape of the honey bee gut microbiota
Source: Nat Commun. 2019 Jan 25;10:446. doi: 10.1038/s41467-019-08303-0 (PMC6347622; doi:10.1038/s41467-019-08303-0)
Supplement: Supplementary file 3 — Description of Additional Supplementary Files [file 41467_2019_8303_MOESM3_ESM.pdf]

Description of Additional Supplementary Files

File Name: Supplementary Data 1
Description: Metadata for the genomes included in the current reference database. Genomes highlighted in bold were included in the reduced database used for SNV profiling.

File Name: Supplementary Data 2
Description: Summary of universally conserved metagenomic gene families without a close hit to the reference database. Sequences of 33 universally conserved gene families were extracted from the assemblies of all samples, and blasted against the current reference database. The table displays summary statistics on the subset of the sequences without a close hit (< 95% BLAST nucleotide alignment identity). For each sample, "Tot_orthologs" contains the total number of universal orthologs without a close hit to the reference database, and "Median_orthologs_per_family" contains the corresponding median number per family. All sequences without a close hit to the database were blasted against the NBCI non-redundant protein database, and the taxonomic placement of the most frequent blast hits is given in the last two columns. "Hit frequency" correspond to the number of sequences supporting the taxonomic placement.

File Name: Supplementary Data 3
Description: Variome gene families, and their correlation coefficients with SDPs. Data for each gene family assigned to the variome, per phylotype. SDP_core_db: gene families corresponding to SDP core gene families in the current reference database (shared among all genomes of a given SDP, and absent from other SDPs of the same phylotype), cor_coeff: pearson correlation coefficients calculated between the abundance of SDPs/phylotypes and the gene family, COG_cats: COG categories assigned to genes contained within each gene family (based on eggNOG annotations), Genefam_annot: non-redundant list of annotations assigned to genes contained within each gene family (based on eggNOG annotations).

File Name: Supplementary Data 4
Description: Genomic islands identified in reference genomes based on the gene families assigned to the variome. Sample_freq: the fraction of samples where at least 80% of the gene families contained in the island were present, Main_cogs: the three most frequent COG annotations on gene families contained in the island, Fraction_NA/S_cog: the fraction of gene families contained in the island without COG annotation, or annotated as COG S, Fraction_phage_NA_annot: the fraction of gene families contained in the island annotated as phage-related, hypothetical or without annotation by eggNOG. Gene ids for all gene-families can be found in Supplementary Data 3, from which all strains included in the gene-families can be inferred.
