# Supplementary material for: Genomic diversity landscape of the honey bee gut microbiota
Source: Nat Commun. 2019 Jan 25;10:446. doi: 10.1038/s41467-019-08303-0 (PMC6347622; doi:10.1038/s41467-019-08303-0)
Supplement: Supplementary file 8 — Reporting Summary [file 41467_2019_8303_MOESM8_ESM.pdf]

nature research

Corresponding author(s): Kirsten Ellegaard, Philipp Engel

Reporting Summary

Nature Research wishes to improve the reproducibility of the work that we publish. This form provides structure for consistency and transparency in reporting. For further information on Nature Research policies, see Authors & Referees and the Editorial Policy Checklist.

Statistical parameters

When statistical analyses are reported, confirm that the following items are present in the relevant location (e.g. figure legend, table legend, main text, or Methods section).

n/a	Confirmed	
☐	☒	The exact sample size (*n*) for each experimental group/condition, given as a discrete number and unit of measurement
☐	☒	An indication of whether measurements were taken from distinct samples or whether the same sample was measured repeatedly
☐	☒	The statistical test(s) used AND whether they are one- or two-sided<br>*Only common tests should be described solely by name; describe more complex techniques in the Methods section.*
☐	☒	A description of all covariates tested
☐	☒	A description of any assumptions or corrections, such as tests of normality and adjustment for multiple comparisons
☐	☒	A full description of the statistics including central tendency (e.g. means) or other basic estimates (e.g. regression coefficient) AND variation (e.g. standard deviation) or associated estimates of uncertainty (e.g. confidence intervals)
☐	☒	For null hypothesis testing, the test statistic (e.g. *F*, *t*, *r*) with confidence intervals, effect sizes, degrees of freedom and *P* value noted<br>*Give P values as exact values whenever suitable.*
☒	☐	For Bayesian analysis, information on the choice of priors and Markov chain Monte Carlo settings
☒	☐	For hierarchical and complex designs, identification of the appropriate level for tests and full reporting of outcomes
☒	☐	Estimates of effect sizes (e.g. Cohen's *d*, Pearson's *r*), indicating how they were calculated
☒	☐	Clearly defined error bars<br>*State explicitly what error bars represent (e.g. SD, SE, CI)*

Our web collection on statistics for biologists may be useful.

Software and code

Policy information about availability of computer code

Data collection	2x100bp paired-end sequencing was done on an Illumina HiSeq 2500 instrument. A genomic reference database was constructed using genomes publicly available on Genbank, as specified in methods section ("construction of a genomic database")
Data analysis	The following open source software was employed: bwa (0.7.15), samtools (1.2), picard tools, SPAdes (3.9.0), eggNOG mapper (1.0.3), fastQC (0.11.4), Trimmomatic (0.35), OrthoFinder (0.3.0), mafft (7.221), RAxML (8.1.24), metaSNV. Custom scripts were written in perl (5.18.2), R (3.5.1) and bash, all of which have been made available on Zenodo (doi: 10.5281/zenodo.1479668)

For manuscripts utilizing custom algorithms or software that are central to the research but not yet described in published literature, software must be made available to editors/reviewers upon request. We strongly encourage code deposition in a community repository (e.g. GitHub). See the Nature Research guidelines for submitting code & software for further information.

Data

Policy information about availability of data

All manuscripts must include a data availability statement. This statement should provide the following information, where applicable:

- Accession codes, unique identifiers, or web links for publicly available datasets
- A list of figures that have associated raw data
- A description of any restrictions on data availability

Code and data availability: The raw data has been deposited in the sequence read archive (SRA), with the accession nb: SRP150166. Documentations of the workflow, including all scripts, databases and result files, are available on Zenodo, with doi: 10.5281/zenodo.1479668. All scripts were written in perl, bash or R. Statistics and plots were likewise done in R (using packages: ggplot2, gridExtra, reshape, gplots, RColorBrewer).

Field-specific reporting

Please select the best fit for your research. If you are not sure, read the appropriate sections before making your selection.

☒ Life sciences ☐ Behavioural & social sciences ☐ Ecological, evolutionary & environmental sciences

For a reference copy of the document with all sections, see nature.com/authors/policies/ReportingSummary-flat.pdf

Life sciences study design

All studies must disclose on these points even when the disclosure is negative.

Sample size	Strain-level variability in gut microbiota composition among honey bees of the same colony was unknown prior to the current study. The sample size (nb of individuals per timepoint/colony) therefore represent a best guess, based on the data from the only available previous metagenomic study (which consisted of 150 pooled individuals)
Data exclusions	Sample "DrY2_W5" was excluded from analysis related to core community compositional changes, due to the abnormal amount of unmapped reads. This is stated clearly in the results, and the unusual profile of this sample is shown in Supplementary Figure 2, together with the other samples
Replication	No replication was done.
Randomization	Not applicable.
Blinding	Not applicable.

Reporting for specific materials, systems and methods

Materials & experimental systems

n/a	Involved in the study
☒	☐ Unique biological materials
☒	☐ Antibodies
☒	☐ Eukaryotic cell lines
☒	☐ Palaeontology
☐	☒ Animals and other organisms
☒	☐ Human research participants

Methods

n/a	Involved in the study
☒	☐ ChIP-seq
☒	☐ Flow cytometry
☒	☐ MRI-based neuroimaging

Animals and other organisms

Policy information about studies involving animals; ARRIVE guidelines recommended for reporting animal research

Laboratory animals	Not applicable
Wild animals	Age-controlled honey bees (Apis mellifera carnica) were collected from the Engel lab apiary, as specified in methods section (sample collection)

Field-collected samples | Not applicable
